# Understanding the Adaptive Growth Strategy of *Lactobacillus plantarum* by *In Silico* Optimisation

**DOI:** 10.1371/journal.pcbi.1000410

**Published:** 2009-06-12

**Authors:** Bas Teusink, Anne Wiersma, Leo Jacobs, Richard A. Notebaart, Eddy J. Smid

**Affiliations:** 1 Top Institute Food and Nutrition (WCFS), Wageningen, The Netherlands; 2 NIZO Food Research BV, Ede, The Netherlands; 3 Center for Molecular and Biomolecular Informatics (NCMLS), Radboud University Nijmegen Medical Center, Nijmegen, The Netherlands; 4 Kluyver Centre for Genomics of Industrial Fermentation, Delft, The Netherlands; University of Virginia, United States of America

## Abstract

In the study of metabolic networks, optimization techniques are often used to predict flux distributions, and hence, metabolic phenotype. Flux balance analysis in particular has been successful in predicting metabolic phenotypes. However, an inherent limitation of a stoichiometric approach such as flux balance analysis is that it can predict only flux distributions that result in maximal yields. Hence, previous attempts to use FBA to predict metabolic fluxes in *Lactobacillus plantarum* failed, as this lactic acid bacterium produces lactate, even under glucose-limited chemostat conditions, where FBA predicted mixed acid fermentation as an alternative pathway leading to a higher yield. In this study we tested, however, whether long-term adaptation on an unusual and poor carbon source (for this bacterium) would select for mutants with optimal biomass yields. We have therefore adapted *Lactobacillus plantarum* to grow well on glycerol as its main growth substrate. After prolonged serial dilutions, the growth yield and corresponding fluxes were compared to *in silico* predictions. Surprisingly, the organism still produced mainly lactate, which was corroborated by FBA to indeed be optimal. To understand these results, constraint-based elementary flux mode analysis was developed that predicted 3 out of 2669 possible flux modes to be optimal under the experimental conditions. These optimal pathways corresponded very closely to the experimentally observed fluxes and explained lactate formation as the result of competition for oxygen by the other flux modes. Hence, these results provide thorough understanding of adaptive evolution, allowing *in silico* predictions of the resulting flux states, provided that the selective growth conditions favor yield optimization as the winning strategy.

## Author Summary

Being able to predict the metabolic fluxes and growth rate of a microorganism is an important topic in microbial systems biology. One approach, constraint-based modeling, uses a reconstructed metabolic network and optimization techniques to make such predictions. Although widely used, the success of this approach depends on a number of important assumptions. First, it assumes that evolutionary forces have shaped the metabolism towards optimality of, in most cases, growth rate. Second, through the nature of the modeling approach, it assumes that microorganisms maximize the growth rate through optimizing the yield on the growth substrate. Despite successes of the approach in model organisms such as *Saccharomyces cerevisiae* and *Escherichia coli*, we have previously observed that the approach fails in *Lactobacillus plantarum*, a lactic acid bacterium that clearly does not optimize its yield on glucose but “wastes” glucose by producing lactic acid. In the current study we provide evidence that *L. plantarum* does optimize its yield when grown under a poor carbon condition, i.e., when grown on glycerol as its main carbon source. The study provides new insight in when the application of *in silico* optimization techniques can be expected to be predictive.

## Introduction

The role of mathematical modeling in the study of microbial physiology has increased considerably by the development of genome-scale metabolic models [1],[2]. For an increasing number of microorganisms such a genome-scale metabolic model is available (for review see [1]). These models can be used for a number of purposes, and a large set of different methods, so-called constraint-based modeling techniques, have been developed in the past years to accommodate these goals [3]. Successful use of genome-scale metabolic models have ranged from exploration of gene lethality [4], definition of metabolic context for integrative bioinformatics [5] and the study of pathway evolution [6], and for guidance in metabolic engineering [7] as well as prediction of adaptive evolution outcomes [8].

In many of these studies flux balance analysis (FBA) was used. FBA uses optimization of an objective function to find a subset of optimal states in the large solution space of possible states that is shaped by mass balance and capacity constraints [3],[9]. In a recent study, different objective functions were tested to the extent that they could predict actual flux states under different conditions [10]. This study demonstrated that different objective functions were needed to describe the flux states under different conditions. Notably, under energy limitation, optimization of biomass yield appeared to be the best objective function. This is in line with earlier studies in which biomass formation was taken as objective to predict functional states [11].

However, we have recently demonstrated that *Lactobacillus plantarum*, a lactic acid bacterium that is found in nutrient-rich niches such as decomposed plant material, does not conform to this consensus [12]. Rather, even under energy-limited chemostat conditions at relatively low growth rates (20% of maximal specific growth rate), homolactic fermentation was still observed. Although L. plantarum and most other lactic acid bacteria do not have a functional TCA cycle and do not respire, they have an alternative pathway that yields more ATP per glucose: mixed acid fermentation (with acetate, ethanol and formate as main products). FBA invariably predicted the mixed acid fermentation pathway rather than homolactic fermentation, and hence predicted growth rates were too high. Note that these are the conditions where Schütz *et al* found good predictions with FBA for *E. coli* [10]. Clearly, this lactic acid bacterium is tuned to use glucose inefficiently, which we attributed to the evolutionary history of this bacterium, being adapted to rich nutritional environments [12]. Moreover, many other microorganisms display inefficient (overflow) metabolism, especially under high-glucose conditions, but even in glucose-limited chemostats above a certain critical dilution rate, such as ethanol fermentation in *Saccharomyces cerevisiae* [13], or acetate formation in *Escherichia coli* [14]. This behavior cannot be predicted a priori by FBA; it can only be described when including additional capacity constraints, e.g. on the oxidative phosphorylation pathways in the corresponding metabolic networks [15],[16].

To fully appreciate these results, it is crucial to understand very precisely what FBA assumes and what it predicts. Under many conditions, especially in the laboratory and under adaptive evolution protocols, growth rate is a good proxy for fitness. Thus, an optimization with respect to growth rate seem an appropriate modeling strategy, and that is what FBA does: it most often optimizes the growth rate ([9], see *Methods*). However, genome-scale metabolic models are stoichiometric models that can only relate input rates and output rates [17]:
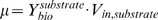
(1)


**Figure 1:**
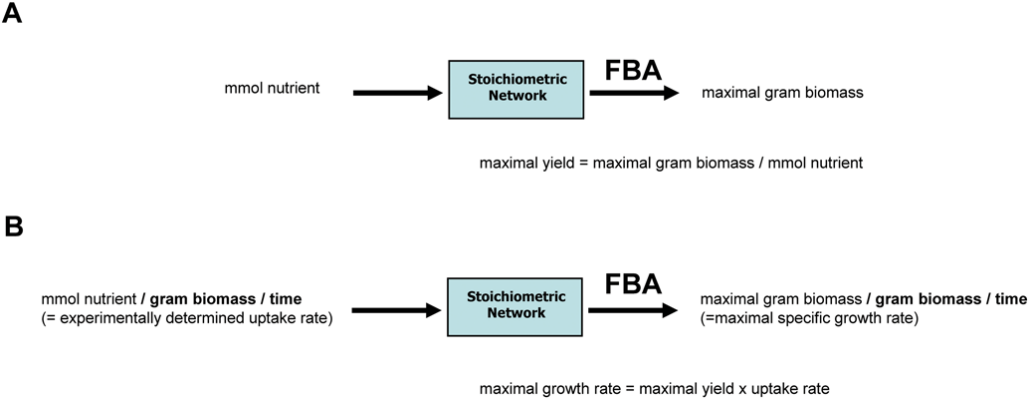
Principle of stoichiometric modeling and Flux Balance Analysis. (A) A stoichiometric network can be used, with FBA, for optimization of maximal yield of biomass on a certain nutrient. (B) by providing an experimentally measured input rate (capacity constraint in constraint-based modeling terms), FBA predicts a specific growth rate. The two situations are, however, exactly the same except for some scaling factor (indicated in bold). In both cases, a flux distribution through the stoichiometric network will be found that maximized the yield of biomass on the nutrient.

Where *μ* is the specific growth rate (units h^−1^), *V_in,substrate_* is the uptake rate of the growth substrate (units mmol h^−1^ gDW^−1^), and *Y* is the yield of biomass with respect to the substrate (units gDW mmol^−1^). If in Eq 1 we want to predict the growth rate, we have to specify the input rate. FBA simply finds the highest yield *Y* such that the growth rate is maximal at the specified input rate. So, even though a *rate* is maximized in FBA (*μ* in Eq. 1), it is through the *yield* *Y* that this is achieved. Indeed, maximizing yield or rate using stoichiometric models is essentially the same, as illustrated in Figure 1. This should make clear that, when applying growth rate optimization in FBA to predict flux distributions, in practice the underlying biological assumption is that metabolic efficiency (high yield) is the strategy through which the organism reaches its maximal growth rate.

There are potentially other strategies that lead to enhanced fitness. These include a very fast (but metabolically inefficient) consumption of substrates, closely related to high growth rates [18]. An alternative is the production of toxic substances (toxins or metabolic waste products such as ethanol or weak acids [19]), which are often produced by low-yield (overflow) pathways. Thus, both high growth rate and fast acidification of the medium, may have been combined by lactic acid bacteria to colonize rich-nutrient environments. Therefore, the validity of the assumptions of FBA (with biomass yield as objective function) is strongly organisms and condition specific [10],[20].

In line with the above reasoning, we realized that even though the behavior of *L. plantarum* cannot be predicted by FBA under the usual, sugar-rich conditions, we may be able to force *L. plantarum* to choose a strategy of high yield by adapting it under poor nutrient conditions. If so, FBA should become predictive. By serial dilution of *L. plantarum* on a medium containing glycerol as main carbon source, we selected strains that were able to grow an order of magnitude faster than the wild type on glycerol. FBA analysis proved to be able to predict the metabolic behavior of a selected adapted strain as a function of the oxygen uptake rate. To gain a more thorough insight into the characteristics of the observed metabolic behavior, elementary flux mode analysis was applied and it was shown that the organism was able to select the 3 optimal flux modes out of almost 2700 possible modes to maximize the ATP yield on the given substrates.

These results provide a thorough understanding of the limitations to methods that predict flux states after adaptive evolution based on FBA, but also provides handles (in the forms of specific cultivation conditions) to overcome them.

## Results/Discussion

### Adaptation of *L. plantarum* to growth on glycerol medium

Glycerol is a substrate that is not unknown to lactobacilli, but often is co-fermented with a fermentable carbon source [21] or undefined substrates present in complex media [22]. Growth of *L. plantarum* with glycerol as main carbon source in chemically defined medium has not been described, but our previously developed genome-scale metabolic model predicted that growth on such medium should be possible (using the Minimization of Metabolic Adjustment (MoMA) algorithm. In this algorithm, the state in the new situation (growth on glycerol) is sought that minimizes the distance with the original state (growth on glucose), thereby mimicking the immediate effect of a medium change [23], results not shown). We tested growth under three different conditions: anaerobic growth, aerobic growth, and respiratory growth. Respiratory growth means that beside oxygen, the medium is supplied with heme and menaquinone: this reconstitutes a functional respiratory chain in which electrons donated to NADH dehydrogenase result in proton translocation by a *bd*-cytochrome oxidase [24].

When we tried to cultivate *L. plantarum* on glycerol medium, we observed very slow growth under anaerobic conditions (OD_600_ of 0.1 after 48 h; growth rate too slow to be accurately measured). Under aerobic or respiratory conditions, no growth was observed at all. We attribute the failure to grow initially under aerobic conditions to oxygen-related stress, the cost of which was apparently higher than the potential benefit in terms of ATP yield (we will come back to this later). By applying the widely-used serial dilution protocol [25], we aimed at selecting mutant strains that were able to grow faster and therefore would be able to take over the population. Although we cannot rule out selective pressure on other possible fitness attributes (e.g. during the stationary phase and lag phase), growth rate is expected to be the dominant fitness determinant under these conditions.

**Figure 2:**
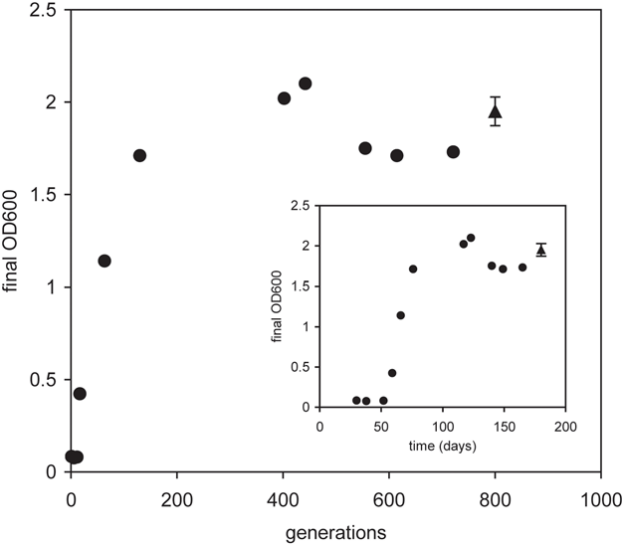
Improvement of growth yield during the course of adaptive evolution, as a function of number of generations (main figure) or time (insert). Closed circles represent the behaviour of the culture, the closed triangle shows the behaviour of the isolated strain NZ1405. There is variability that seems related to the specific batch of medium, which is chemically defined but rather complex. Within one batch of medium, the variability is indicated for strain NZ1405 (closed triangles, standard deviation of four replicates).

After two months and approximately 20 generations, the OD_600_ values increased substantially (from 0.1 at day 0 to 0.4, measured after 48 h). At this point we tried to grow the adapted culture again under respiratory conditions, and growth was now possible and higher OD's were reached (OD_600_ of 0.4 versus 0.8 for anaerobic versus respiratory, respectively). Hence, from this point on the adaptive evolution protocol was continued under respiratory conditions. Figure 2 shows the final OD improvements over time; growth rate increased during this period to a final growth rate of 0.26 h^−1^, which was more than a magnitude higher than the initial growth rate. After 150 days and approximately 500 generations, no further increase in final OD and growth rate was observed. At this point single colonies were picked from the adapted culture, and growth rate was assessed in the individual strains. The variation in growth rate among different strains was not different from that of a single one (data not shown). Moreover, sequencing of the promoter region of the glpKDF-operon (encoding the glycerol catabolic genes necessary for glycerol usage) of six clones identified the same point mutation (results not shown), indicating that the culture was homogeneous. We have therefore picked one of these six colonies (designated NZ1405) for further detailed physiological characterization. In Figure 2, the final OD of the isolated strain NZ1405 is also plotted to show that it behaved in line with the adapted culture.

### Characterization of adapted strain NZ1405

We characterized growth and product formation in the original shake-flask that was used for the adaptive evolution (Table 1). However, to measure oxygen uptake rates accurately, NZ1405 was also grown under controlled batch fermentation conditions. In the shake flasks growth rates were slightly higher (0.26±0.01 h^−1^ and 0.23±0.04 h^−1^ for shake flask and fermentor, respectively). Based on the fact that (i) the amounts of substrates consumed and products formed per unit of biomass were very similar in both settings, and (ii) the dissolved oxygen during the measurements in the fermentor was above 20%, which is generally more than enough for oxygen *concentration* not to be limiting for the oxygen consumption *rate*, the oxygen uptake rate in the fermentor and the shake flask should be very similar (see Table 1). Surprisingly, despite aerobic conditions, lactate was still the main product formed (90% of glycerol uptake), while acetate and minor amounts of acetoin were also detected. Ethanol that was present in the medium as solvent for menaquinone (see Methods) turned out to be used as additional substrate, as was the citrate that is also present in CDM. Amino acid uptake is detailed in Dataset S1.

**Table 1:** Amount of substrates consumed and products formed per gram biomass during mid-exponential growth in shake flask or in controlled fermentor setting.

Uptake or production (mmol gDW^−1^)	Shake flask			Fermentor			Model constraints robustness analysis	
	Average	±	Stdev	Average	±	Stdev	LB	UB
Growth rate (h^−1^)	0.26	−	0.01	0.23	−	0.04	0	∞
Glycerol	−40.0	±	2.41	−47.1	±	2.11	−42.1^a^	−38.0
citric acid	−1.91	±	0.25	−0.40	±	0.09	−2.1	−1.7
Lactate	33.5	±	1.40	30.7	±	0.89	0	∞
Pyruvate	0.15	±	0.06	0.01	±	0.06	0	∞
Formate	0	±	0	0	±	0	0	∞
Acetate	5.83	±	0.51	4.76	±	0.78	−∞	∞
Ethanol	−4.27	±	0.53	−5.35	±	1.22	−∞	∞
Acetoin	0.74	±	0.02	1.14	±	0.10	0	∞
Succinate	0	±	0	0	±	0	0	∞
Oxygen	ND			−22.3	±	3.14	Robustness parameter	

**Figure 3:**
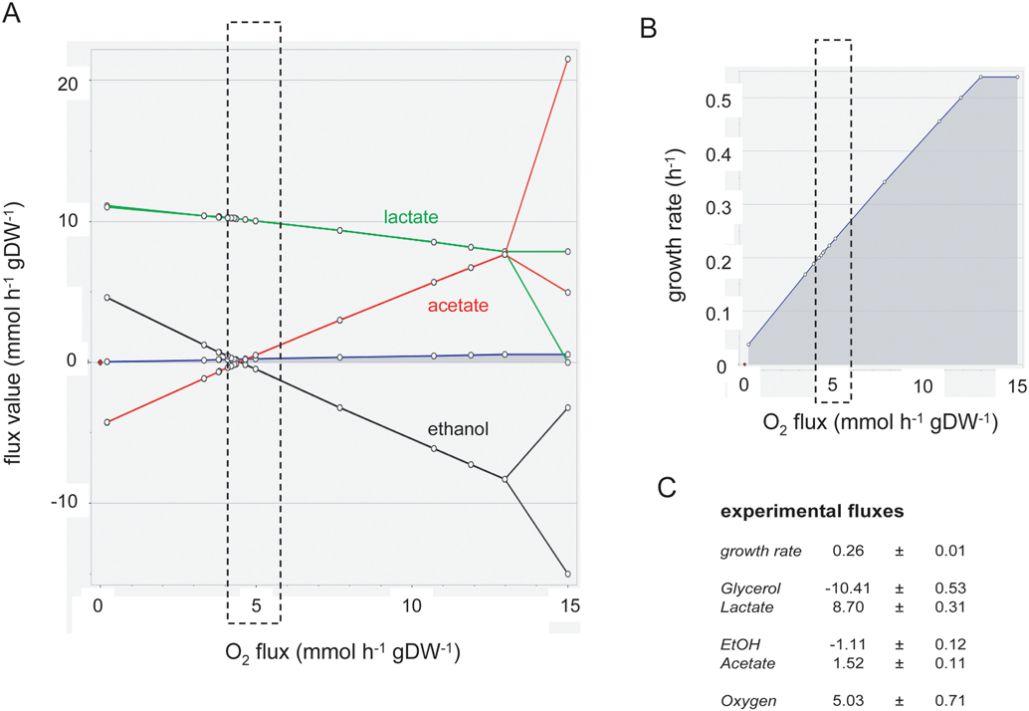
Robustness analysis of *in silico* growth on glycerol as a function of the oxygen uptake rate and comparison with experimental data (calculated from the data presented inTable 1). (A) Impact of oxygen uptake on optimal lactate (green), acetate (red) and ethanol (black) fluxes. Dashed box indicate the oxygen consumption rate measured experimentally. Above and uptake rate of 13 mmol h^−1^ gDW^−1^ growth is no longer energy limited, resulting in variability in fluxes: the diverging lines indicate the maximum and minimum flux value at each oxygen uptake rate. (B) Impact of oxygen uptake on the growth rate. (C) Experimentally derived fluxes are included for comparison.

Data presented are yield data averaged over 2–4 independent experiments during growth between an OD600 of 0.2 and 0.7. Yield data are in mmol gDW^−1^. Negative values indicate uptake of the compound. The last two columns indicate the constraints used for the robustness analysis of Figure 3. Other constraints can be found in Dataset S1.

ND not determined; LB lower bound; UB upper bound.

a Presented unit is in mmol gDW^−1^ for comparison to measured fluxes. To get to flux constraints with unit mmol h^−1^ gDW^−1^ (as presented in Dataset S1) multiply with the growth rate (0.26 h^−1^).

### Comparing experimental results with flux balance analysis predictions

The endpoint of adaptive evolution as characterized above was compared to optimal behavior predicted by the genome-scale model under respiratory conditions. During the very first simulations, however, we noticed that very high biomass yields could be obtained, with concomitant production of only CO_2_ and water as final products. The simulations implicated phosphoketolase (PKL) in the reverse direction than usual, the usual direction being cleavage of the xylulose 5-P as phosphoketolase is involved in pentose catabolism in many lactic acid bacteria [26]. In a detailed analysis provided in Text S1, it was shown *in silico* that aerobically, glycerol can be completely converted to CO_2_ and water, yielding as much as 3 moles of ATP per mole of glycerol by substrate level phosphorylation alone. Clearly, the experimental results do not comply with an active phosphoketolase cycle. The observed behavior was therefore dismissed as an interesting future target for perhaps synthetic biological improvements, and the model was adjusted to prevent the phosphoketolase cycle from running (see Table S1 for details of the genome-scale model version 3.0 used in this study).

The complex medium that was used, created another problem that needed to be solved: How to set reasonable constraints on the many medium components present in the medium? When all measured fluxes, including the amino acid fluxes, were set as constraints and biomass yield was optimized, a growth rate of 0.324 h^−1^ was found, which fits reasonably well with, but is clearly higher than, the rate of 0.26 h^−1^ found experimentally. There are two possible reasons for the discrepancy. First, through the adaptation, the (stoichiometric) efficiency of some metabolic processes may have improved that have not been incorporated in the model. These relate to possible changes in efficiency of transport systems, to efficiencies in proton leakage and/or ATPase stoichiometry (number of protons pumped per ATP molecule), or to the assembly of biomass precursors into new cells (the growth-related ATP coefficient). Improvements in these processes, however, would lead to lower *in silico* growth rate predictions. The second possibility is that the ATP requirement for maintenance has changed. Based on the observation that respiratory growth on glycerol was not possible for the wild type (see above), we reasoned that the ATP maintenance under respiratory conditions could be higher than the maintenance measured previously [12] under anaerobic growth on glucose. An ATP maintenance of 3.94 mmol h^−1^ gDW^−1^ was required to obtain the observed growth rate of 0.26 h^−1^ under experimentally determined flux constraints. This maintenance coefficient is in the same order as that measured for respiratory organisms, such as 5.87 mmol h^−1^ gDW^−1^ for *E. coli* [27]. With the higher maintenance coefficient, we indeed predict with MoMA that wild type (unadapted) *L. plantarum* cannot grow on glycerol (maximal growth rate is zero, compared to 0.03 h^−1^ at the original, lower, maintenance coefficient).

Reassuring as this result may be, the main question is, whether we can predict fluxes. Since we have so many input fluxes, the issue is what the minimum set of input fluxes is that needs to be fixed by experimental observations in order to prevent the system from becoming unbounded. This issue has not been specifically addressed before, as most studies have been performed on organisms that grow on minimal salts media with one carbon source [8],[20]. In our case, however, 18 amino acids, oxygen, glycerol, acetate, ethanol and citrate were present as substrates, potentially influencing each other's impact on optimal growth.

To tackle this problem, we first dismissed a major impact of amino acid metabolism in the light of the smaller fluxes compared to primary metabolism (Table 1 and Dataset S1, see also [12]). Hence, the maximum uptake rate of amino acids was set by the experimental data. To understand the impact and interdependencies of the organic substrates and oxygen, we performed elementary flux mode (EFM) analysis [28]. EFM analysis calculates all possible pathways between defined sources and sinks (called external metabolites) that are elementary, i.e. in which each step in the pathway is essential [28]. EFM analysis was performed for primary metabolism only, under the assumption that ATP production by primary metabolism is determining growth yield under these conditions. Thus, we assume that the improvement in growth performance is caused by improvements in ATP generation. This is by no means a generally valid assumption, but reasonable in this particular case, as many biomass components are taken up from the medium, and most of the organic substrates are available for ATP generation rather than biomass components.

We found 2669 EFMs and 531 different overall EFM stoichiometries with net ATP production (see Text S2 for a detailed analysis). Most modes required oxygen as a substrate, which demonstrated the importance of oxygen uptake in ATP production. However, there were also anaerobic modes that, if left unconstraint, could yield unlimited amounts of ATP. Anaerobic modes with either citrate or glycerol as sole substrates were found, but not with ethanol or acetate as the only substrate. We therefore concluded that it would be essential to constrain the input of glycerol, citrate and oxygen in order to have bound the fluxes through the metabolic network. The acetate and ethanol uptake rates should be bounded by the constraints on these three fluxes.

Since most EFM's were dependent on variable amounts of oxygen we decided to fix the uptake rates of citrate and glycerol at the measured values, leave the acetate and ethanol capacity constraints unrestricted, and predict the growth behavior as a function of the oxygen uptake rate (Figure 3). Flux variability analysis was used to test the uniqueness of the product formation predictions for each oxygen uptake rate. Product formation was almost completely determined by the oxygen availability (i.e. no flux variability was allowed in the optimal solution). At unrestricted oxygen uptake rates, acetate would be the only end product (result not shown).

Figure 3 shows that at the measured oxygen uptake rate of 5 mmol h^−1^ gDW^−1^, a good agreement between experimental fluxes and model prediction was observed. Specifically, growth rate, lactate, acetate and ethanol fluxes were close to the predicted fluxes under maximization of the growth rate of FBA. It therefore appears that the conditions under which *L. plantarum* was adapted, promoted metabolic strategies with optimal yield, rendering FBA predictive.

Initially, however, it was a surprise that FBA predicted lactate formation as an optimal strategy, and not acetate (which at first sight would yield more ATP). To understand this result from the robustness analysis, we went back to the elementary flux modes. We applied a constraint-based optimization on all 2669 EFMs to ask which combination of EFMs would lead to the highest ATP production yield, given the measured fluxes of oxygen, glycerol and citrate as constraints (see methods). The analysis identified three EFMs:
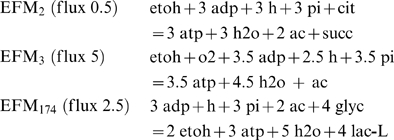



Thus, EFMs 2, 3 and 174 (numbered according to the table in the Text S2 that provides all EFMs) are optimal for ATP yield. Minimization and maximization of the flux through each EFM at the maximal ATP production rate yielded “EFM variability” for three more EFMs (EFMs 177, 178 and 179). The overall stoichiometry of these modes, however, turned out to be linear combinations of the modes 2, 3 and 174, thus providing no different end products to produce maximal ATP production rates. EFM_2_ uses the citrate, EFM_3_ the oxygen and EFM_174_ consumes the glycerol. This analysis provides an explanation for the lactate production being optimal under these conditions (found by FBA and experimentally observed). When 1 mole of oxygen were available, anaerobic conversion of glycerol into lactate (EFM_174_), and conversion of the ethanol into acetate using the oxygen (EFM_3_), yields more ATP than using the oxygen for the most efficient glycerol conversion into acetate (EFM_190_: 1.5 o2+5 adp+4 h+5 pi+glyc = 5 atp+7 h2o+co2+ac). Please note that the fact that lactate is predicted by FBA under respiratory growth is not in contradiction with the inability of FBA to predict lactate under anaerobic growth on glucose: the conditions are completely different.

The anaerobic conversion of citrate and ethanol into acetate and succinate (EFM_2_) was unexpected, since no succinate is being formed experimentally. Rather, acetoin was formed, a well known product from citrate metabolism implied in pH homeostasis [29]. There are, however, two anaerobic modes (EFM_2258_ and EFM_2383_) that convert citrate into acetoin and acetate with a slightly lower yield (2 atp per citrate compared to 3 atp for citrate plus ethanol in EFM 2). Since in the optimization EFM_2_ contributes only 6% of the total ATP yield, the small difference between EFM_2_ and EFM_2258_ or EFM_2383_ may have provided too small a selection pressure to allow selection of a strain which reprogrammed the network from acetoin to succinate production.

In summary, the constraint-based EFM analysis provided valuable additional mechanistic insight in the many options that the metabolic network had (as a function of the oxygen consumption rate), and strikingly, the adapted strain selected out of the 2669 possibilities the 3 EFMs that were (almost) optimal for ATP production under oxygen uptake limitation. The use of elementary flux modes to decipher the optimal use of multiple nutrients is a new application of the EFM concept, underpinning the optimality of the solution used by the adapted strain. Under these relatively poor conditions, realizing an optimal yield appears the best strategy to win the battle of fitness. However, this is provided a restricted uptake of oxygen, and so one may argue that yield maximization is still not predictive in our case, the optimum being full oxidation to acetate. Indeed, the situation is analogous to the suboptimal acetate production by *E. coli* grown on glucose being predicted through a capacity constraint on oxygen uptake [16]. For *L. plantarum* with predominantly anaerobic habitats, and given the potential harm of oxygen metabolism, a limit in the oxygen uptake rate should perhaps be expected. We feel therefore that the match between optimality prediction (under this oxygen limitation) and observed fluxes is striking and does strongly suggest yield optimality under these conditions. In any case, yield optimality was useful to interpret and predict the observed fluxes.

This study shows how rational design of the selection conditions can be used to steer the strategy for fitness of an organism into a desired direction. It appears, at first glance, that this conclusion was reached before, especially by the studies of the Palsson group on *E. coli* [8],[20],[30],[31]. These studies demonstrated, however, that even though in their experiments growth rate is always selected for, *in silico* predicted maximal growth rates and corresponding optimal consumption rates (in the form of the line of optimality, LO) do not always fit the data. Although the authors discuss the observations, they do not, however, provide an explanation when to expect a deviation from optimality. The case of *L. plantarum* described in ref [12] and in the current paper provides such an explanation by clearly illustrating the essential difference between the objective of the cell (fitness, here growth rate) and the strategy to reach it (one of which is to maximize yield, the somewhat hidden assumption in FBA).

A clear understanding of the difference will be crucial in appreciating the usefulness and limitations of optimization techniques in systems and synthetic biology. For example, the recently developed OptKnock strategy took the idea of being able to predict adaptive evolution one step further by identifying knockout targets that would result in the alignment of growth rate and byproduct formation as optimization objective, thereby forcing cells to increase fitness by increasing product formation [32]. From the current study it is clear that this and other FBA-based approaches will only be successful when maximizing growth rate through optimal yield is the strategy used by the organisms in the first place. Hence, in low-substrate fed-batch or chemostat conditions this is more likely to work than in high-substrate batch conditions (see also [10]); it is also more likely to work for poor carbon sources such as glycerol or lactate, than it will be for sugars such as glucose or maltose.

## Methods

### Strain and growth conditions

The bacterial strain used in this study was *Lactobacillus plantarum* WCFS1. Cells were grown on Chemically Defined Medium (CDM) as described previously [33], supplemented with 5 g/l of the desired carbon source (glucose or glycerol). During the first two months of this study the strain was grown under anaerobic conditions at 37°C on CDM supplemented with 0.5 (m/v) % glycerol. Full grown cultures were used to re-inoculate fresh media (dilution 1∶50). For respiratory growth, the culture was grown in 125 ml Erlenmeyer flasks containing 25 ml of CDM supplemented with heme (3.07 µM, Sigma) and vitamin K12 (18 µM, Sigma, dissolved in 10 µl ethanol) and incubated under aeration (150 rpm) at 37°C. As the yield and growth rate increased the amount of inoculum was changed to a dilution of 1∶100. During the adaptation process, cultures were regularly collected and stored in 15% glycerol at −80°C. For preparation of Figure 2, the glycerol stocks were revived on MRS medium, washed with CDM containing glycerol, and cultured on CDM with glycerol. The resultant overnight culture was used as inoculum to estimate growth improvement (as OD_600_ reached in 24–48 hours).

At the end of the adaptation process, judged by no further growth improvements, 96 single colony isolates were isolated from a CDM agarose plate (CDM medium containing 1–1.5% Agar, LABM Limited, Bury, UK) and grown overnight in a KC Junior, micro-titer reader (Bio-Tek, Vermont) shaken with intermediate intensity at 37°C. OD_600_ and growth rates were compared. Subsequently, one isolate (NZ1405) was chosen for further characterization.

### Physiological characterization of NZ1405

Strain NZ1405 was cultivated in Erlenmeyer shake flasks as described above (4 independent experiments). At mid-log phase (OD_600_ of 0.7), samples were collected for analysis of amino acids, organic compounds and dry weight, as described previously [12]. To determine the oxygen consumption rate, NZ1405 was cultivated in duplicate in a temperature - and pH-controlled batch fermentor at 37°C, pH 5.5 (0.5 L volume in a 1 L fermentor, Applikon, Schiedam, The Netherlands). The medium was flushed with air (257 ml/min) at a stirring speed of 150 rpm. The oxygen transfer rate (K_L_a) in this setting was determined directly before inoculation of the fermentor by a switch to N_2_ gas and back to air and recording the change in dissolved oxygen. The determined value (11.4±0.4 h^−1^) was used to calculate the oxygen diffusion rate (which equals the oxygen consumption rate by the cells at steady state) during growth:
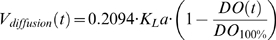
(2)


The average oxygen consumption rate during mid-exponential growth (OD_600_ 0.2–1) was used.

### In silico computations

The previously developed genome-scale metabolic model of *L. plantarum* WCFS1 was used, with minor modifications. Details of the current model, version 3.0, is described in supporting Table S1. All calculations were done with Simpheny software (Genomatica Inc, San Diego, USA), or with the COBRA Toolbox described in [34]. For flux balance analysis, the optimization problem is formulated as:
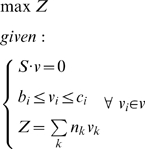
(3)with *Z* being the biomass equation, modeled as a sink of biomass components fluxes *v_k_* with stoichiometric coefficients *n_k_*. Since these stoichiometric coefficients have unit mmol gDW^−1^, and all fluxes *v* have unit mmol h^−1^ gDW^−1^, *Z* has unit h^−1^ and represent the specific growth rate. *S* represents the stoichiometry matrix and *v* is the flux vector. The scalars *b_i_* and *c_i_* are the lower and upper capacity constraints, respectively, for each flux *v_i_*.

The robustness analysis presented in Figure 2 was carried out by varying the oxygen consumption rate between 0 and 15 mmol h^−1^ gDW^−1^, while fixing the amino acid, citrate and glycerol consumption rates at their measured value (average±standard deviation, see also Table 1). Protons, water and CO_2_, as well as acetate and ethanol were allowed to exchange freely, whereas all other exchange fluxes were set at (b = 0, c = 99999), i.e. the corresponding compounds could only be produced. At each oxygen consumption rate, the growth rate was maximized according to Eq 3. At the resultant maximal growth rate, the exchange fluxes for lactate, glycerol, ethanol, acetate and citrate were maximized and minimized to test for alternative optimal solutions [35].

Elementary flux modes were calculated using Metatool 4.9 [36]. Primary metabolism, including respiration and proton-mediated transport, was cut out of the large-scale metabolic network and the proper internal and external metabolites were set as indicated in the Metatool input file provided in Text S2. The constraint-based EFM analysis was formulated as:
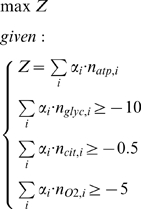



Here, *α_i_* is the flux going through EFM_i_, *n_atp,i_* the yield of ATP of EFM_i_ (*n_glyc,i_*, *n_cit,i_* and *n_O2,i_* are defined accordingly for glycerol, citrate and oxygen, respectively). The optimization procedure thus provides the set of α's that maximize the total ATP yield given a set of capacity constraints on input fluxes (the summations). The optimizations were performed using glpk (http://www.gnu.org/software/glpk/).

## Supporting Information

Dataset S1 Model capacity constraints. This file contains amino acid uptake rates and corresponding flux constraints used in the model, and more details on model results.
(0.24 MB DOC)

Table S1 Model details. This file contains the abbreviations, reactions and the gene-protein-reaction associations of *L. plantarum* model v3 used in this study.
(0.28 MB XLS)

Text S1 The phosphoketolase cycle. This file contains more information on the phosphoketolase cycle that was discovered in the network of *L. plantarum*.
(1.60 MB DOC)

Text S2 Elementary Flux Mode analysis. This file contains the Metatool input file used for elementary flux mode analysis, and the resulting EFMs.
(0.66 MB DOC)
