# Supplementary material for: Understanding the Adaptive Growth Strategy of Lactobacillus plantarum by In Silico Optimisation
Source: PLoS Comput Biol. 2009 Jun 12;5(6):e1000410. doi: 10.1371/journal.pcbi.1000410 (PMC2690837; doi:10.1371/journal.pcbi.1000410)
Supplement: Dataset S1 — Model capacity constraints. This file contains amino acid uptake rates and corresponding flux constraints used in the model, and more details on model results. (0.24 MB DOC) [file pcbi.1000410.s001.doc]

# Supporting Dataset S1: amino acid metabolism, flux constraints used in the model and model results.

In this section, we provide details on the amino acid fluxes that were measured in the shake-flask experiments corresponding to Table 1 in the main text. The data are provided in Table I-1. Based on all these experimental data combined, a set of flux constraints was formulated for the simulations as described in the paper (**Table I-2**). Using these constraints in the genome-scale metabolic model of *L. plantarum* version 3.0 and minimizing for growth yield resulted in a maximal growth rate of 0.31 h^-1^ (see main text). For the robustness analysis presented in Figure 2, flux constraints were adjusted from Table I-2 as specified in **Table I-3**. In the robustness analysis the oxygen uptake rate was varied between 0 and -15 mmol h^-1^ gDW^-1^ and FBA was used to find the maximal growth yield. In **Table I-4**, a flux variability was performed using the setting of the robustness analysis, but at the experimentally determined oxygen uptake rate of -5 mmol h^-1^ gDW^-1^, to see what the flexibility in the networks is. It is observed that amino acid metabolism contributes quite significantly to the growth rate, as indicated by fluxes hitting their maximal uptake rate with a span of zero (**Table I-4**).

| **amino acid** | **Uptake** | | | | **Flux** | | | | |
| --- | --- | --- | --- | --- | --- | --- | --- | --- | --- |
|  | *mmol gDW^-1^* | | | | *mmol h^-1^ gDW^-1^* | | | | |
|  |  |  | |  | |  |  | |  |
| Alanine | -0.460 | ± | 0.135 | | | -0.120 | ± | 0.035 | |
| Arginine | -0.149 | ± | 0.039 | | | -0.039 | ± | 0.010 | |
| Aspartic acid | -0.552 | ± | 0.214 | | | -0.144 | ± | 0.056 | |
| Cystine | -0.192 | ± | 0.047 | | | -0.050 | ± | 0.012 | |
| Glutamic acid | -0.571 | ± | 0.138 | | | -0.148 | ± | 0.036 | |
| Glycine | -0.276 | ± | 0.069 | | | -0.072 | ± | 0.018 | |
| Histidine | -0.181 | ± | 0.057 | | | -0.047 | ± | 0.015 | |
| Isoleucine | -0.298 | ± | 0.039 | | | -0.077 | ± | 0.010 | |
| Leucine | -0.485 | ± | 0.093 | | | -0.126 | ± | 0.024 | |
| Lysine | -0.301 | ± | 0.035 | | | -0.078 | ± | 0.009 | |
| Methionine | -0.929 | ± | 0.029 | | | -0.242 | ± | 0.008 | |
| Phenylalanine | -0.229 | ± | 0.030 | | | -0.060 | ± | 0.008 | |
| Proline | -0.224 | ± | 0.121 | | | -0.058 | ± | 0.031 | |
| Serine | -0.463 | ± | 0.203 | | | -0.120 | ± | 0.053 | |
| Threonine | 0.174 | ± | 0.130 | | | 0.045 | ± | 0.034 | |
| Tryptophane | -0.061 | ± | 0.006 | | | -0.016 | ± | 0.002 | |
| Tyrosine | 0.268 | ± | 0.361 | | | 0.070 | ± | 0.094 | |
| Valine | -0.323 | ± | 0.033 | | | -0.084 | ± | 0.009 | |

**Table I-1**. Amino acid consumption rates during mid-exponential growth of NZ1405 on CDM with glycerol, heme and vitamin K2. CDM contains all amino acids except glutamine and asparagine (Teusi*nk et* al, 2005). Fluxes are in mmol h^-1^ gDW^-1^.

| **Category** | **ABBREVIATION** | **OFFICIAL NAME** | **LOWER BOUND** | **UPPER BOUND** |
| --- | --- | --- | --- | --- |
|  |  |  |  |  |
| vitamins and nucleobases | *exchange flux constraints* | |  |  |
|  | EX_pnto-R(e) | (R)-Pantothenate exchange | -0.00099897 | 0 |
|  | EX_btn(e) | Biotin exchange | -0.003196292 | 0 |
|  | EX_fol(e) | Folate exchange | -0.000704489 | 0 |
|  | EX_nac(e) | Nicotinate exchange | -0.001344933 | 0 |
|  | EX_4abz(e) | 4-Aminobenzoate exchange | -0.022844089 | 0 |
|  | EX_pydam(e) | Pyridoxamine exchange | -0.00659617 | 0 |
|  | EX_pydxn(e) | Pyridoxine exchange | -0.003038431 | 0 |
|  | EX_ribflv(e) | Riboflavin exchange | -0.00066243 | 0 |
|  | EX_thm(e) | Thiamin exchange | -0.001033876 | 0 |
|  | EX_ins(e) | Inosine exchange | -0.005796261 | 0 |
|  | EX_orot(e) | Orotate exchange | -0.010021923 | 0 |
|  | EX_thymd(e) | Thymidine exchange | -0.006419 | 0 |
|  | EX_ade(e) | Adenine exchange | -0.023013305 | 0 |
|  | EX_gua(e) | Guanine exchange | -0.020574809 | 0 |
|  | EX_ura(e) | Uracil exchange | -0.027739251 | 0 |
|  | EX_xan(e) | Xanthine exchange | -0.020439448 | 0 |
|  |  |  |  |  |
| growth substrates | EX_ac(e) | Acetate exchange | 1.404742722 | 1.627214724 |
|  | EX_cit(e) | Citrate exchange | -0.551749789 | -0.4437038 |
|  | EX_glyc(e) | Glycerol exchange | -10.93797962 | -9.879191436 |
|  | EX_etoh(e) | Ethanol exchange | -1.227750732 | -0.995087127 |
|  |  |  |  |  |
| amino acids | EX_ala-L(e) | L-Alanine exchange | -0.154598643 | -0.084599755 |
|  | EX_arg-L(e) | L-Arginine exchange | -0.04889622 | 0 |
|  | EX_asp-L(e) | L-Aspartate exchange | -0.199127146 | -0.087964335 |
|  | EX_cys-L(e) | L-Cysteine exchange | -0.062108734 | -0.037564568 |
|  | EX_glu-L(e) | L-Glutamate exchange | -0.184201959 | -0.112536941 |
|  | EX_gly(e) | Glycine exchange | -0.089873994 | -0.053806634 |
|  | EX_his-L(e) | L-Histidine exchange | -0.061744505 | -0.03220564 |
|  | EX_ile-L(e) | L-Isoleucine exchange | -0.08771451 | -0.067227696 |
|  | EX_leu-L(e) | L-Leucine exchange | -0.150256188 | -0.102135228 |
|  | EX_lys-L(e) | L-Lysine exchange | -0.0874543 | 0 |
|  | EX_met-L(e) | L-Methionine exchange | -0.249107321 | -0.234021167 |
|  | EX_phe-L(e) | L-Phenylalanine exchange | -0.067486805 | -0.051653905 |
|  | EX_pro-L(e) | L-Proline exchange | -0.089605816 | -0.026874055 |
|  | EX_ser-L(e) | Exchange for Serine | -0.173330011 | -0.067511878 |
|  | EX_thr-L(e) | L-Threonine exchange | 0.011446936 | 0.078866938 |
|  | EX_trp-L(e) | L-Tryptophan exchange | -0.017333039 | -0.01429291 |
|  | EX_tyr-L(e) | L-Tyrosine exchange | -0.024113376 | 0.163538184 |
|  | EX_val-L(e) | L-Valine exchange | -0.092461266 | -0.075239005 |
|  | EX_asn-L(e) | L-Asparagine exchange | 0 | 999999 |
|  | EX_gln-L(e) | L-Glutamine exchange | 0 | 999999 |
|  |  |  |  |  |
| inorganic compounds | EX_co2(e) | CO2 exchange | -999999 | 999999 |
|  | EX_h(e) | H+ exchange | -999999 | 999999 |
|  | EX_h2o(e) | H2O exchange | -999999 | 999999 |
|  | EX_o2(e) | O2 exchange | -5.742140372 | -4.321480044 |
|  | EX_na1(e) | Sodium exchange | -999999 | 999999 |
|  | EX_mn2(e) | Mn2+ exchange | -999999 | 999999 |
|  | EX_pi(e) | Phosphate exchange | -13.1971172 | 999999 |
|  | EX_so4(e) | Sulfate exchange | 0 | 999999 |
|  | EX_nh4(e) | Ammonium exchange | -2.264061947 | 999999 |
|  |  |  |  |  |
| potential products | EX_lac-L(e) | L-Lactate exchange | 8.396728025 | 9.011731007 |
|  | EX_pyr(e) | Pyruvate exchange | 0.024461483 | 0.051410683 |
|  | EX_for(e) | Formate exchange | 0.0 | 0.0 |
|  | EX_actn-R(e) | (R)-Acetoin exchange | 0.18824018 | 0.195632305 |
|  | EX_succ(e) | Succinate exchange | 0.0 | 0.0 |
|  | EX_acald(e) | Acetaldehyde exchange | 0 | 999999 |
|  | EX_mal-L(e) | L-Malate exchange | 0 | 999999 |
|  | EX_glyclt(e) | Glycolate exchange | 0 | 999999 |
|  | EX_btd-RR(e) | (R,R)-2,3-Butanediol exchange | 0 | 999999 |
|  | EX_diact(e) | Diacetyl exchange | 0 | 999999 |
|  | EX_2aeppn(e) | (2-Aminoethyl)phosphonate exchange | 0 | 999999 |
|  | EX_4abut(e) | 4-Aminobutanoate exchange | 0 | 999999 |
|  |  |  |  |  |
| amino acid-derived | EX_2hxic-L(e) | L-2-hydroxyisocaproate exchange | 0 | 999999 |
| flavor compounds | EX_2mba(e) | 2-methyl butanoic acid exchange | 0 | 999999 |
|  | EX_2mbal(e) | 2-Methylbutanal exchange | 0 | 999999 |
|  | EX_2mbol(e) | 2-methylbutanol exchange | 0 | 999999 |
|  | EX_2mpa(e) | 2-methylpropanoic acid exchange | 0 | 999999 |
|  | EX_2mpal(e) | 2-methylpropanal exchange | 0 | 999999 |
|  | EX_2mpol(e) | 2-methylpropanol exchange | 0 | 999999 |
|  | EX_34hplac(e) | (R)-3-(4-Hydroxyphenyl)lactate exchange | 0 | 999999 |
|  | EX_3mba(e) | 3-methylbutanoic acid exchange | 0 | 999999 |
|  | EX_3mbal(e) | 3-methylbutanal exchange | 0 | 999999 |
|  | EX_3mbol(e) | 3-methylbutanol exchange | 0 | 999999 |
|  | EX_bzal(e) | Benzaldehyde exchange | 0 | 999999 |
|  | EX_imlac(e) | imidazole lactate exchange | 0 | 999999 |
|  | EX_indlac(e) | Indolelactate exchange | 0 | 999999 |
|  | EX_methal(e) | Methional exchange | 0 | 999999 |
|  | EX_pacald(e) | Phenylacetaldehyde exchange | 0 | 999999 |
|  | EX_pea(e) | Phenylethyl alcohol exchange | 0 | 999999 |
|  | EX_phenol(e) | Phenol exchange | 0 | 999999 |
|  | EX_phlac(e) | Phenyl lactate exchange | 0 | 999999 |
|  |  |  |  |  |
|  | *internal flux constraints* |  |  |  |
| non-default settings | ATPM | ATP maintenance requirement | 0.29 | 0.29 |
|  | PKL | phosphoketolase | 0 | 999999 |
|  | PFL | Formate C-acetyltransferase | 0 | 0 |
|  | PDH | Pyruvate dehydrogenase | 0 | 0 |
|  |  |  |  |  |
| respiratory settings | CAT | Catalase | 0 | 999999 |
|  | CYTB_B2 | menaquinol oxidase (7:1 protons) | 0 | 999999 |
|  |  |  |  |  |

**Table I_2.** Measured and non-zero flux constraints used for the model, based on experimental data or default settings where no data was available. Internal flux constraints that deviate from the default settings are also given. PKL was set irreversible to prevent a phosphoketolase cycle as explained in **Supplementary Information II**. PFL is oxygen sensitive and is therefore inactive during aerobic growth. PDH was set to zero as there is no experimental proof that this enzyme is active in *L. plantarum*. ATP maintenance was set based on previous studies on anaerobic growth on glucose (Teusi*nk et* al, 2006). The heme-dependent catalase reaction, as well as the essential component in the respiratory chain, the menaquinol oxidase, were switched on in respiratory mode only. Hence aerobic and respiratory conditions differ by these two reactions.

| **ABBREVIATION** | **OFFICIAL NAME** | **LOWER BOUND** | **UPPER BOUND** |
| --- | --- | --- | --- |
|  |  |  |  |
| *exchange flux constraints* |  |  |  |
| EX_ac(e) | Acetate exchange | -999999 | 999999 |
| EX_cit(e) | Citrate exchange | -0.5 | -0.5 |
| EX_glyc(e) | Glycerol exchange | -10.4 | -10.4 |
| EX_etoh(e) | Ethanol exchange | -999999 | 999999 |
|  |  |  |  |
| EX_o2(e) | O2 exchange | -15 | 0 |
| EX_lac-L(e) | L-Lactate exchange | 0 | 999999 |
| EX_pyr(e) | Pyruvate exchange | 0 | 999999 |
| EX_for(e) | Formate exchange | 0 | 999999 |
| EX_actn-R(e) | (R)-Acetoin exchange | 0 | 999999 |
| EX_succ(e) | Succinate exchange | 0 | 999999 |
|  |  |  |  |
| *internal flux constraints* |  |  |  |
| ATPM | ATP maintenance requirement | 3.94 | 3.94 |

**Table I-3**. Flux constraints that were changed compared to Table I-2 for the robustness analysis of Figure 3 in the main text.

| **Abbreviation** | **LB** | **UB** | **Min** | **Max** | **Span** |
| --- | --- | --- | --- | --- | --- |
| EX_glyc(e) | -10.400 | -10.400 | -10.400 | -10.400 | 0.000 |
| EX_o2(e) | -5.000 | -5.000 | -5.000 | -5.000 | 0.000 |
| EX_etoh(e) | -Infinity | Infinity | -0.811 | -0.811 | 0.000 |
| EX_cit(e) | -0.500 | -0.500 | -0.500 | -0.500 | 0.000 |
| EX_pi(e) | -13.197 | Infinity | -0.299 | -0.299 | 0.000 |
| EX_met-L(e) | -0.249 | -0.234 | -0.249 | -0.234 | 0.015 |
| EX_asp-L(e) | -0.199 | -0.088 | -0.199 | -0.199 | 0.000 |
| EX_glu-L(e) | -0.184 | -0.113 | -0.184 | -0.184 | 0.000 |
| EX_ser-L(e) | -0.173 | -0.068 | -0.173 | -0.173 | 0.000 |
| EX_ala-L(e) | -0.155 | -0.085 | -0.155 | -0.155 | 0.000 |
| EX_leu-L(e) | -0.150 | -0.102 | -0.150 | -0.150 | 0.000 |
| EX_val-L(e) | -0.092 | -0.075 | -0.092 | -0.092 | 0.000 |
| EX_ile-L(e) | -0.088 | -0.067 | -0.088 | -0.088 | 0.000 |
| EX_phe-L(e) | -0.067 | -0.052 | -0.067 | -0.067 | 0.000 |
| EX_his-L(e) | -0.062 | -0.032 | -0.062 | -0.062 | 0.000 |
| EX_gly(e) | -0.090 | -0.054 | -0.055 | -0.055 | 0.000 |
| EX_lys-L(e) | -0.087 | 0.000 | -0.042 | -0.042 | 0.000 |
| EX_cys-L(e) | -0.062 | -0.038 | -0.038 | -0.038 | 0.000 |
| EX_pro-L(e) | -0.090 | -0.027 | -0.027 | -0.027 | 0.000 |
| EX_tyr-L(e) | -0.024 | 1.64E-01 | -0.024 | -0.024 | 0.000 |
| EX_arg-L(e) | -0.049 | 0.000 | -0.023 | -0.023 | 0.000 |
| EX_ura(e) | -0.028 | Infinity | -0.021 | -0.021 | 0.000 |
| EX_gua(e) | -0.021 | Infinity | -0.021 | -0.021 | 0.000 |
| EX_ade(e) | -0.023 | Infinity | -0.020 | -0.020 | 0.000 |
| EX_trp-L(e) | -0.017 | -0.014 | -0.017 | -0.017 | 0.000 |
| EX_orot(e) | -0.010 | 0.000 | -0.010 | -0.010 | 0.000 |
| EX_ins(e) | -0.006 | Infinity | -0.006 | -0.006 | 0.000 |
| EX_thymd(e) | -0.006 | Infinity | -0.004 | -0.004 | 0.000 |
|  |  |  |  |  |  |
| EX_2hxic-L(e) | 0 | Infinity | 0.000 | 0.105 | 0.105 |
| EX_3mbol(e) | 0 | Infinity | 0.000 | 0.105 | 0.105 |
| EX_34hplac(e) | 0 | Infinity | 0.008 | 0.008 | 0.000 |
| EX_thr-L(e) | 0.011 | 0.079 | 0.011 | 0.011 | 0.000 |
| EX_indlac(e) | 0 | Infinity | 0.014 | 0.014 | 0.000 |
| EX_pea(e) | 0 | Infinity | 0.048 | 0.048 | 0.000 |
| EX_imlac(e) | 0 | Infinity | 0.052 | 0.052 | 0.000 |
| EX_2mpol(e) | 0 | Infinity | 0.058 | 0.058 | 0.000 |
| EX_4abut(e) | 0 | Infinity | 0.058 | 0.092 | 0.034 |
| EX_2mbol(e) | 0 | Infinity | 0.063 | 0.063 | 0.000 |
| biomass_LPL6.0 | 0.239 | 0.239 | 0.239 | 0.239 | 0.000 |
| EX_methal(e) | 0 | Infinity | 0.252 | 0.267 | 0.015 |
| EX_co2(e) | -Infinity | Infinity | 0.523 | 0.669 | 0.146 |
| EX_succ(e) | 0 | Infinity | 0.564 | 0.597 | 0.034 |
| EX_nh4(e) | -2.264 | Infinity | 0.588 | 0.629 | 0.041 |
| EX_ac(e) | -Infinity | Infinity | 0.794 | 0.794 | 0.000 |
| EX_h(e) | -Infinity | Infinity | 8.767 | 8.929 | 0.163 |
| EX_lac-L(e) | 0 | Infinity | 9.469 | 9.480 | 0.011 |
| EX_h2o(e) | -Infinity | Infinity | 11.887 | 11.921 | 0.034 |

**Table I-4**. Result of a flux variability analysis at the measured oxygen consumption rate of -5 mmol h^-1^ gDW^-1^, and under the constraints described for the robustness analysis of Figure 2. Data are sorted by flux. **LB**: lower flux bound, **UB** upper flux bound, **Min**: minimal allowed flux, **Max**: maximal allowed flux, **span**: absolute difference between Min and Max.

**References**

Teusink B, Van Enckevort FHJ, Francke C, Wiersma A, Wegkamp A, Smid EJ, Siezen RJ (2005) *In silico* reconstruction of the metabolic pathways of *Lactobacillus plantarum*: comparing predictions of nutrient requirements with those from growth experiments. *Appl Environ Microbiol* **71:** 7253-7262.

Teusink B, Wiersma A, Molenaar D, Francke C, de Vos WM, Siezen RJ, Smid EJ (2006) Analysis of growth of *Lactobacillus plantarum* WCFS1 on a complex medium using a genome-scale metabolic model. *J Biol Chem* **281:** 40041-40048.
