# Supplementary material for: Understanding the Adaptive Growth Strategy of Lactobacillus plantarum by In Silico Optimisation
Source: PLoS Comput Biol. 2009 Jun 12;5(6):e1000410. doi: 10.1371/journal.pcbi.1000410 (PMC2690837; doi:10.1371/journal.pcbi.1000410)
Supplement: Text S1 — The phosphoketolase cycle. This file contains more information on the phosphoketolase cycle that was discovered in the network of L. plantarum. (1.60 MB DOC) [file pcbi.1000410.s003.doc]

# Supporting Text S2: phosphoketolase cycle.

### *A new pathway discovered: the phosphoketolase cycle*

The endpoint of adaptive evolution as characterized above was compared to optimal behavior predicted by performing FBA on our genome-scale model. During the very first simulations, however, we noticed that very high biomass yields could be obtained, with concomitant production of only CO_2_ and water as final products. The simulations implicated phosphoketolase (PKL) in the reverse direction than usual, the usual direction being cleavage of the xylulose 5-P as phosphoketolase is involved in pentose catabolism in many lactic acid bacteria [1]:

pi + xu5p-D <==> actp + g3p + h2o Eq 1

When PKL is allowed to proceed in the reverse reaction, glycerol can be converted completely into CO2 and H2O, according to the classical combustion reaction, yielding 3 ATP:

glyc + 3 o2 + 3 adp + 3 pi => 3 co2 + 3 h2o + 3 atp Eq 2

The explanation for this overall reaction is that phosphoketolase allows acetyl phosphate to reenter glycolysis via the ketolases and aldolase that constitute the non-oxidative part of the pentose phosphate pathway. The overall stoichiometry of the combination of these reactions is:

3 actp + 2 atp => 2 g3p Eq 3

Together with glycolysis a cycle is formed which we have designated the phosphoketolase cycle (**Figure II-1**). In the model, the cycle not only runs on glycerol, but also with glucose (or any other sunstrate that results in glyceraldehyde 3-phosphate, the actual substrate of the cycle). To illustrate this, a flux distribution in the phosphoketolase cycle is shown with glucose as substrate. The result is the classical combustion reaction with 6 moles of ATP per mole of glucose (**Figure II-2**)

Clearly, the experimental results do not comply with an active phosphoketolase cycle. Although there are no apparent thermodynamic reasons why the reaction would not be reversible (the equilibrium reaction is close to 1 based on data from [2]), attempts to measure phosphoketolase activity in the reverse direction, using *in vitro* assays, failed to demonstrate activity [3]. It may be that the enzyme has been kinetically optimized by evolution (within the thermodynamic boundaries of the Haldane relationship between maximum rates and affinities) towards the direction of pentose breakdown, and that 500 generations was not enough to accumulate sufficient mutations in the enzyme to work efficiently in the reverse direction. The observed behavior was therefore dismissed as an interesting future target for perhaps synthetic biological improvements, and the model was adjusted to prevent the phosphoketolase cycle from running (see **Supplementary Information IV** for details of the genome-scale model version 3.0 used in this study).

**References**

1. Posthuma CC, Bader R, Engelmann R, Postma PW, Hengstenberg W, et al. (2002) Expression of the xylulose 5-phosphate phosphoketolase gene, xpkA, from Lactobacillus pentosus MD363 is induced by sugars that are fermented via the phosphoketolase pathway and is repressed by glucose mediated by CcpA and the mannose phosphoenolpyruvate phosphotransferase system. Appl Environ Microbiol 68: 831-837.

2. Kummel A, Panke S, Heinemann M (2006) Putative regulatory sites unraveled by network-embedded thermodynamic analysis of metabolome data. Mol Syst Biol 2: 2006 0034.

3. Heath EC, Hurwitz J, Horecker BL, Ginsburg A (1958) Pentose fermentation by Lactobacillus plantarum. I. The cleavage of xylulose 5-phosphate by phosphoketolase. J Biol Chem 231: 1009-1029.

**Figure II-1**: the phosphoketolase cycle that results in complete “combustion” of glycerol (or glucose, see Figure II-2) into co2 and h2o. Abbreviations: PKL phosphoketolase; TAL transaldolase; TKT transketolase; PYROX pyruvate oxidase; GLYK glycerol kinase; G3PD1 glycerol 3-phosphate dehydrogenase; TPI triose phosphate isomerase; actp acetyl phosphate; pyr pyruvate; g3p glyceraldehyde 3-phosphate; glc glucose. For clarity only involvement of atp is shown (and not adp, h2o and h).


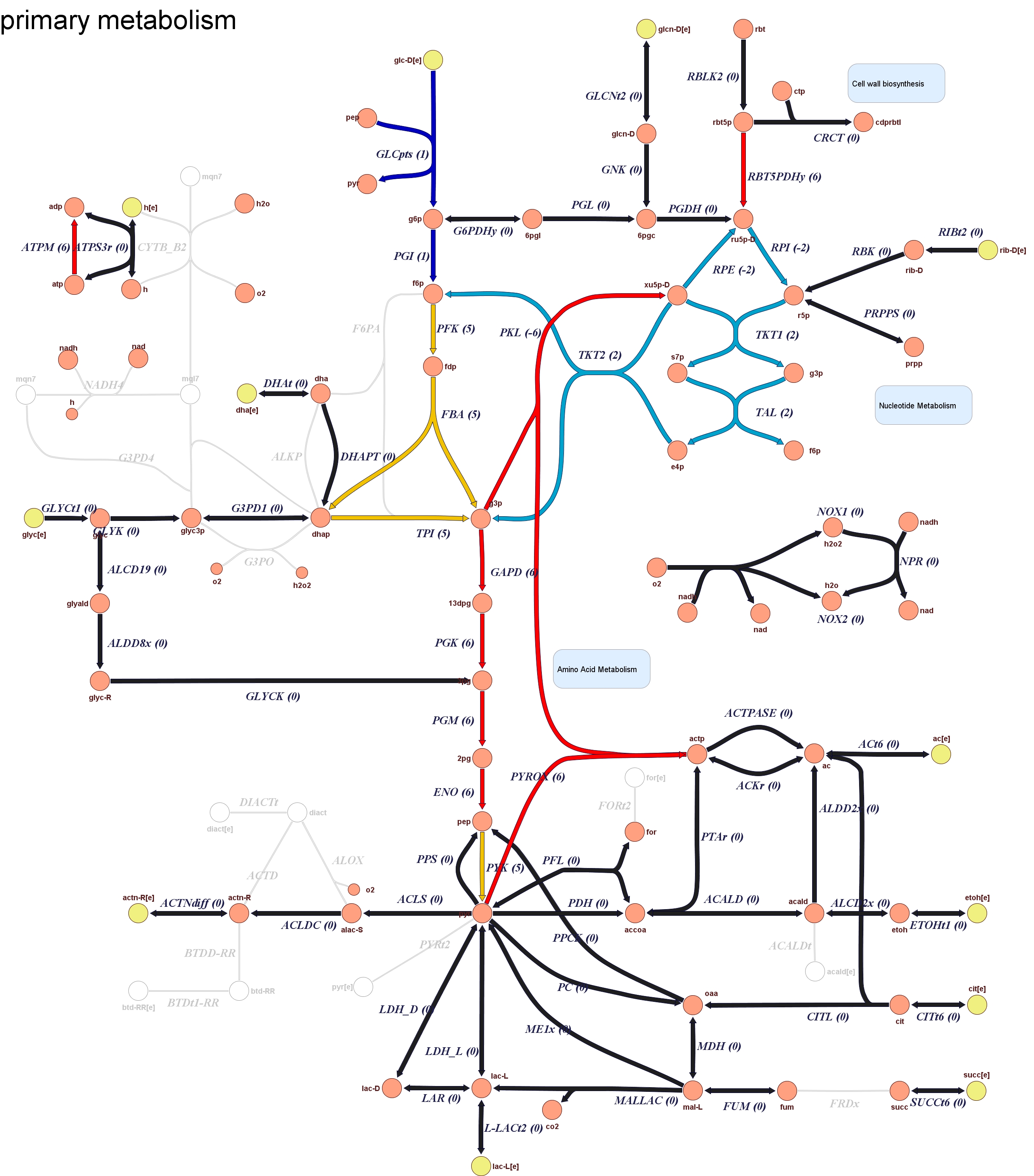


**Figure II-2**. Flux distribution of the phosphoketolase cycle converting 1 mmol h^-1^ gDW^-1^ of glucose into 6 ATP, similar to equation II-2 (but with 2 g3p per glucose instead of 1 g3p per glycerol).
