# Supplementary material for: Understanding the Adaptive Growth Strategy of Lactobacillus plantarum by In Silico Optimisation
Source: PLoS Comput Biol. 2009 Jun 12;5(6):e1000410. doi: 10.1371/journal.pcbi.1000410 (PMC2690837; doi:10.1371/journal.pcbi.1000410)
Supplement: Text S2 — Elementary Flux Mode analysis. This file contains the Metatool input file used for elementary flux mode analysis, and the resulting EFMs. (0.66 MB DOC) [file pcbi.1000410.s004.doc]

# Supporting Text S3: elementary flux mode analysis of glycerol metabolism

In this section, we address the question: what is the minimum set of input fluxes that need to be fixed by experimental observations in order to prevent the system from becoming unbounded? We are specifically interested in primary metabolism, and have set the amino acid flux constraints to the measured values, as explained in the main text. Amino acid fluxes and other constraints can be found in **Supplementary Information I**. We used elementary flux mode analysis [1] to address the question.

Primary metabolism, including respiration and proton-mediated transport, was cut out of the large-scale metabolic network and the proper internal and external metabolites were set as indicated in the Metatool input file given in **Table III-2**. Running this file with Metatool 4.9 [2] resulted in 2669 EFM’s (not shown). Many of these EFM’s have the same overall stoichiometry. Relevant for our analysis are only those overall stoichiometries that result in net ATP production. Analysis of the 2669 EFM’s resulted in 531 unique overall stoichiometries with such net ATP production. These stoichiometries are presented in **Table III-3**. Analysis of these stoichiometries reveals that:

1. there are only anaerobic ATP producing modes for glycerol (yellow background) and citrate (pink background).
2. in aerobic modes, ethanol, glycerol and citrate can be used as single inputs to generate ATP, but at varying oxygen to ATP and substrate to ATP ratio’s. The modes therefore “compete” for the oxygen, and FBA will find the best combination of modes to maximize biomass (ATP) production.

Based on the EFM analysis, it is clear that we will have to constrain the oxygen consumption rate, the glycerol consumption rate, and the citrate consumption rate. The ethanol and acetate flux are then bounded by the other rates. In **Figure 3** of the main text, citrate and glycerol were constraint at their measured levels, while oxygen uptake rate was varied.

**References**

1. Schuster S, Fell DA, Dandekar T (2000) A general definition of metabolic pathways useful for systematic organization and analysis of complex metabolic networks. Nat Biotechnol 18: 326-332.

2. Pfeiffer T, Sanchez-Valdenebro I, Nuno JC, Montero F, Schuster S (1999) METATOOL: for studying metabolic networks. Bioinformatics 15: 251-257.

-ENZREV

PGI FBA TPI GAPD PGK PGM ENO TKT1 TKT2 RPE RPI TAL ACKr PTAr ACALD FUM MDH ADK1 HCO3E

G3PD1 F6PA ALCD2x ATPS3r ACt6 CITt6

-ENZIRREV

ACTNdiff ACLS ACLDC BTDD-RR G6PDH PGL PGDH PFK PPS PYK PPCK PC ME1x ALDD2x MALLAC PPA PYROX NOX1 NOX2 NPR

GLYK G3PO ALKP CYTB_B2 NADH4 CITL PKL LDH_L FRDx CAT SUCCt6 MALt6 LACt6

-METINT

xu5p-D(c) 13dpg(c) 2pg(c) 3pg(c) 6pgc(c) 6pgl(c) acald(c) accoa(c) actp(c) amp(c) coa(c) dhap(c) e4p(c) f6p(c) fdp(c) fum(c) g3p(c) g6p(c) h2o2(c) hco3(c) mal-L(c) oaa(c) pep(c) ppi(c) pyr(c) r5p(c) ru5p-D(c) s7p(c)

h(c) glyc3p(c) mql7(c) mqn7(c) dha(c) nad(c) nadh(c) alac-S(c) actn-R(c) cit(c) lac-L(c) ac(c) succ(c)

-METEXT

etoh(c) o2(c) nadp(c) nadph(c) atp(c) adp(c) h2o(c) h(e) pi(c) co2(c) ac(e) lac-L(e) succ(e) mal-L(e)

glyc(c) cit(e) actn-R(e) btd-RR(c)

-CAT

LACt6 : lac-L(c) + h(c) = lac-L(e) + h(e) .

ACt6 : ac(c) + h(c) = ac(e) + h(e) .

CITt6 : cit(c) + h(c) = cit(e) + h(c) .

SUCCt6 : succ(c) + h(c) = succ(e) + h(e) .

MALt6 : mal-L(c) + h(c) = mal-L(e) + h(c) .

ACLS : h(c) + 2.0 pyr(c) = alac-S(c) + co2(c) .

ACLDC : alac-S(c) + h(c) = actn-R(c) + co2(c) .

BTDD-RR : actn-R(c) + h(c) + nadh(c) = btd-RR(c) + nad(c) .

ACTNdiff : actn-R(c) = actn-R(e) .

ACALD : acald(c) + coa(c) + nad(c) = accoa(c) + h(c) + nadh(c) .

ACKr : ac(c) + atp(c) = actp(c) + adp(c) .

ADK1 : amp(c) + atp(c) = 2.0 adp(c) .

ALCD2x : etoh(c) + nad(c) = acald(c) + h(c) + nadh(c) .

ALDD2x : acald(c) + h2o(c) + nad(c) = ac(c) + 2.0 h(c) + nadh(c) .

ENO : 2pg(c) = h2o(c) + pep(c) .

FBA : fdp(c) = dhap(c) + g3p(c) .

FRDx : fum(c) + h(c) + nadh(c) = nad(c) + succ(c) .

FUM : fum(c) + h2o(c) = mal-L(c) .

G6PDH : g6p(c) + nadp(c) = 6pgl(c) + h(c) + nadph(c) .

GAPD : g3p(c) + nad(c) + pi(c) = 13dpg(c) + h(c) + nadh(c) .

HCO3E : co2(c) + h2o(c) = h(c) + hco3(c) .

LDH_L : h(c) + nadh(c) + pyr(c) = lac-L(c) + nad(c) .

MALLAC : h(c) + mal-L(c) = co2(c) + lac-L(c) .

MDH : mal-L(c) + nad(c) = h(c) + nadh(c) + oaa(c) .

ME1x : mal-L(c) + nad(c) = co2(c) + nadh(c) + pyr(c) .

NOX1 : h(c) + nadh(c) + o2(c) = h2o2(c) + nad(c) .

NOX2 : 2.0 h(c) + 2.0 nadh(c) + o2(c) = 2.0 h2o(c) + 2.0 nad(c) .

NPR : h(c) + h2o2(c) + nadh(c) = 2.0 h2o(c) + nad(c) .

PC : atp(c) + hco3(c) + pyr(c) = adp(c) + h(c) + oaa(c) + pi(c) .

PFK : atp(c) + f6p(c) = adp(c) + fdp(c) + h(c) .

PGDH : 6pgc(c) + nadp(c) = co2(c) + nadph(c) + ru5p-D(c) .

PGI : g6p(c) = f6p(c) .

PGK : 13dpg(c) + adp(c) = 3pg(c) + atp(c) .

PGL : 6pgl(c) + h2o(c) = 6pgc(c) + h(c) .

PGM : 3pg(c) = 2pg(c) .

PKL : pi(c) + xu5p-D(c) = actp(c) + g3p(c) + h2o(c) .

PPA : h2o(c) + ppi(c) = h(c) + 2.0 pi(c) .

PPCK : atp(c) + oaa(c) = adp(c) + co2(c) + pep(c) .

PPS : atp(c) + h2o(c) + pyr(c) = amp(c) + 2.0 h(c) + pep(c) + pi(c) .

PTAr : accoa(c) + pi(c) = actp(c) + coa(c) .

PYK : adp(c) + h(c) + pep(c) = atp(c) + pyr(c) .

PYROX : h(c) + o2(c) + pi(c) + pyr(c) = actp(c) + co2(c) + h2o2(c) .

RPE : ru5p-D(c) = xu5p-D(c) .

RPI : r5p(c) = ru5p-D(c) .

TAL : g3p(c) + s7p(c) = e4p(c) + f6p(c) .

TKT1 : r5p(c) + xu5p-D(c) = g3p(c) + s7p(c) .

TKT2 : e4p(c) + xu5p-D(c) = f6p(c) + g3p(c) .

TPI : dhap(c) = g3p(c) .

GLYK : atp(c) + glyc(c) = adp(c) + glyc3p(c) + h(c) .

CYTB_B2 : 2.0 h(c) + mql7(c) + 0.5 o2(c) = 2.0 h(e) + h2o(c) + mqn7(c) .

G3PD1 : glyc3p(c) + nad(c) = dhap(c) + h(c) + nadh(c) .

G3PO : glyc3p(c) + o2(c) = dhap(c) + h2o2(c) .

ALKP : dhap(c) + h2o(c) = dha(c) + pi(c) .

NADH4 : h(c) + mqn7(c) + nadh(c) = mql7(c) + nad(c) .

ATPS3r : adp(c) + 3.0 h(e) + pi(c) = atp(c) + 2.0 h(c) + h2o(c) .

F6PA : f6p(c) = dha(c) + g3p(c) .

CITL : cit(c) = ac(c) + oaa(c) .

CAT : 2.0 h2o2(c) = 2.0 h2o(c) + o2(c) .

**Table III-2**: Metatool input file, indicating reverisible reactions (ENZREV), irreversible reactions (ENZIRREV), internal metabolites that need to be balanced (METINT), external metabolites that act as source or sink (METEXT), and the reaction stoichiometries (CAT). Abbreviations are explained in **Supplementary Information IV**.

| **EFM** | **Reaction** | **02/ATP** | **ATP/S** |
| --- | --- | --- | --- |
| 2258, 2383 | 4 adp(c) + 10 h(e) + 4 pi(c) + 2 cit(e) = 4 atp(c) + 4 h2o(c) + 4 co2(c) + 2 ac(e) + actn-R(e) | 0 | 2.000 |
| 1712, 1878 | etoh(c) + 9.5 adp(c) + 20.5 h(e) + 9.5 pi(c) + 4 cit(e) = 9.5 atp(c) + 8.5 h2o(c) + 8 co2(c) + 5 ac(e) + 2 btd-RR(c) | 0 | 1.900 |
| 1804, 1879 | 7.5 adp(c) + 16.5 h(e) + 7.5 pi(c) + glyc(c) + 3 cit(e) = 7.5 atp(c) + 7.5 h2o(c) + 7 co2(c) + 3 ac(e) + 2 btd-RR(c) | 0 | 1.875 |
| 19, 82, 582 | etoh(c) + 5.5 adp(c) + 8.5 h(e) + 5.5 pi(c) + 2 cit(e) = 5.5 atp(c) + 4.5 h2o(c) + 2 co2(c) + 3 ac(e) + 2 lac-L(e) | 0 | 1.833 |
| 2291 | 11 adp(c) + 19 h(e) + 11 pi(c) + 2 glyc(c) + 4 cit(e) = 11 atp(c) + 11 h2o(c) + 6 co2(c) + 4 ac(e) + 4 lac-L(e) + actn-R(e) | 0 | 1.833 |
| 1755 | 9 adp(c) + 15 h(e) + 9 pi(c) + 2 glyc(c) + 3 cit(e) = 9 atp(c) + 9 h2o(c) + 5 co2(c) + 3 ac(e) + 3 lac-L(e) + btd-RR(c) | 0 | 1.800 |
| 210, 306, 583 | 3.5 adp(c) + 4.5 h(e) + 3.5 pi(c) + glyc(c) + cit(e) = 3.5 atp(c) + 3.5 h2o(c) + co2(c) + ac(e) + 2 lac-L(e) | 0 | 1.750 |
| 1714, 1884 | etoh(c) + 8 adp(c) + 19 h(e) + 8 pi(c) + 4 cit(e) = 8 atp(c) + 7 h2o(c) + 8 co2(c) + 5 ac(e) + 2 btd-RR(c) | 0 | 1.600 |
| 1713, 1881 | 4.75 adp(c) + 9.25 h(e) + 4.75 pi(c) + glyc(c) + 2 cit(e) = 4.75 atp(c) + 4.75 h2o(c) + 4 co2(c) + 3.5 ac(e) + btd-RR(c) | 0 | 1.583 |
| 2 | etoh(c) + 3 adp(c) + 3 h(e) + 3 pi(c) + cit(e) = 3 atp(c) + 3 h2o(c) + 2 ac(e) + succ(e) | 0 | 1.500 |
| 177 | 4.5 adp(c) + 3.5 h(e) + 4.5 pi(c) + 2 glyc(c) + cit(e) = 4.5 atp(c) + 5.5 h2o(c) + ac(e) + 2 lac-L(e) + succ(e) | 0 | 1.500 |
| 1733 | 5.25 adp(c) + 6.75 h(e) + 5.25 pi(c) + 2 glyc(c) + 1.5 cit(e) = 5.25 atp(c) + 6.75 h2o(c) + 2 co2(c) + 1.5 ac(e) + 1.5 succ(e) + btd-RR(c) | 0 | 1.500 |
| 2271 | 6 adp(c) + 8 h(e) + 6 pi(c) + 2 glyc(c) + 2 cit(e) = 6 atp(c) + 8 h2o(c) + 2 co2(c) + 2 ac(e) + 2 succ(e) + actn-R(e) | 0 | 1.500 |
| 20, 83, 585 | 2.75 adp(c) + 3.25 h(e) + 2.75 pi(c) + glyc(c) + cit(e) = 2.75 atp(c) + 2.75 h2o(c) + co2(c) + 2.5 ac(e) + lac-L(e) | 0 | 1.375 |
| 70, 87, 590 | etoh(c) + 4 adp(c) + 7 h(e) + 4 pi(c) + 2 cit(e) = 4 atp(c) + 3 h2o(c) + 2 co2(c) + 3 ac(e) + 2 lac-L(e) | 0 | 1.333 |
| 154 | etoh(c) + 4 adp(c) + 6 h(e) + 4 pi(c) + 2 cit(e) = 4 atp(c) + 3 h2o(c) + 2 co2(c) + 6 ac(e) | 0 | 1.333 |
| 211, 307 | 5 adp(c) + 5 h(e) + 5 pi(c) + 3 glyc(c) + cit(e) = etoh(c) + 5 atp(c) + 6 h2o(c) + co2(c) + 4 lac-L(e) | 0 | 1.250 |
| 584 | 5 adp(c) + 5 h(e) + 5 pi(c) + 3 glyc(c) + cit(e) = 1 etoh(c) + 5 atp(c) + 6 h2o(c) + co2(c) + 4 lac-L(e) | 0 | 1.250 |
| 178 | 6 adp(c) + 4 h(e) + 6 pi(c) + 4 glyc(c) + cit(e) = etoh(c) + 6 atp(c) + 8 h2o(c) + 4 lac-L(e) + succ(e) | 0 | 1.200 |
| 1756 | 3.5 adp(c) + 6.5 h(e) + 3.5 pi(c) + 2 glyc(c) + cit(e) = 1 etoh(c) + 3.5 atp(c) + 4.5 h2o(c) + 3 co2(c) + lac-L(e) + 1 btd-RR(c) | 0 | 1.167 |
| 1805 | 3 adp(c) + 7 h(e) + 3 pi(c) + 1.66667 glyc(c) + cit(e) = 1 etoh(c) + 3 atp(c) + 4 h2o(c) + 3.66667 co2(c) + 1.33333 btd-RR(c) | 0 | 1.125 |
| 1880 | 3 adp(c) + 7 h(e) + 3 pi(c) + 1.66667 glyc(c) + cit(e) = etoh(c) + 3 atp(c) + 4 h2o(c) + 3.66667 co2(c) + 1.33333 btd-RR(c) | 0 | 1.125 |
| 2292 | 3.66667 adp(c) + 7.66667 h(e) + 3.66667 pi(c) + 2 glyc(c) + 1.33333 cit(e) = 1.33333 etoh(c) + 3.66667 atp(c) + 5 h2o(c) + 3.33333 co2(c) + 1.33333 lac-L(e) + actn-R(e) | 0 | 1.100 |
| 1734 | 4 adp(c) + 6 h(e) + 4 pi(c) + 2.66667 glyc(c) + cit(e) = etoh(c) + 4 atp(c) + 6 h2o(c) + 2.66667 co2(c) + succ(e) + 1.33333 btd-RR(c) | 0 | 1.091 |
| 7 | 3 adp(c) + 1 h(e) + 3 pi(c) + 2 glyc(c) + cit(e) = 3 atp(c) + 4 h2o(c) + 4 ac(e) + succ(e) | 0 | 1.000 |
| 155 | 2 adp(c) + 2 h(e) + 2 pi(c) + glyc(c) + cit(e) = 2 atp(c) + 2 h2o(c) + co2(c) + 4 ac(e) | 0 | 1.000 |
| 175 | 2 adp(c) + h(e) + 2 pi(c) + glyc(c) + cit(e) = 2 atp(c) + 2 h2o(c) + ac(e) + lac-L(e) + mal-L(e) | 0 | 1.000 |
| 437 | 2 adp(c) + 3 h(e) + 2 pi(c) + glyc(c) + cit(e) = 2 atp(c) + 2 h2o(c) + co2(c) + ac(e) + 2 lac-L(e) | 0 | 1.000 |
| 2028, 2182 | 2 nadp(c) + 3 adp(c) + 6 h(e) + 3 pi(c) + glyc(c) + 2 cit(e) = 2 nadph(c) + 3 atp(c) + 2 h2o(c) + 5 co2(c) + 3 ac(e) + 1 btd-RR(c) | 0 | 1.000 |
| 2272 | 3 adp(c) + 5 h(e) + 3 pi(c) + 2 glyc(c) + cit(e) = etoh(c) + 3 atp(c) + 5 h2o(c) + 2 co2(c) + succ(e) + actn-R(e) | 0 | 1.000 |
| 2334, 2384 | 2 adp(c) + 6 h(e) + 2 pi(c) + glyc(c) + cit(e) = etoh(c) + 2 atp(c) + 3 h2o(c) + 3 co2(c) + actn-R(e) | 0 | 1.000 |
| 1731 | 4.5 adp(c) + 4.5 h(e) + 4.5 pi(c) + 2 glyc(c) + 3 cit(e) = 4.5 atp(c) + 4.5 h2o(c) + 2 co2(c) + 3 ac(e) + 3 mal-L(e) + btd-RR(c) | 0 | 0.900 |
| 176 | 3.5 adp(c) + 1.5 h(e) + 3.5 pi(c) + 3 glyc(c) + cit(e) = 1 etoh(c) + 3.5 atp(c) + 4.5 h2o(c) + 3 lac-L(e) + mal-L(e) | 0 | 0.875 |
| 1 | etoh(c) + 2.5 adp(c) + 1.5 h(e) + 2.5 pi(c) + 2 cit(e) = 2.5 atp(c) + 1.5 h2o(c) + 3 ac(e) + 2 mal-L(e) | 0 | 0.833 |
| 157 | etoh(c) + 2.5 adp(c) + 4.5 h(e) + 2.5 pi(c) + 2 cit(e) = 2.5 atp(c) + 1.5 h2o(c) + 2 co2(c) + 6 ac(e) | 0 | 0.833 |
| 1716, 1888 | 2.5 adp(c) + 7 h(e) + 2.5 pi(c) + glyc(c) + 2 cit(e) = 2.5 atp(c) + 2.5 h2o(c) + 4 co2(c) + 3.5 ac(e) + btd-RR(c) | 0 | 0.833 |
| 2269 | 5 adp(c) + 5 h(e) + 5 pi(c) + 2 glyc(c) + 4 cit(e) = 5 atp(c) + 5 h2o(c) + 2 co2(c) + 4 ac(e) + 4 mal-L(e) + actn-R(e) | 0 | 0.833 |
| 174 | 3 adp(c) + h(e) + 3 pi(c) + 2 ac(e) + 4 glyc(c) = 2 etoh(c) + 3 atp(c) + 5 h2o(c) + 4 lac-L(e) | 0 | 0.750 |
| 24 | etoh(c) + 1.5 adp(c) + 1.5 h(e) + 1.5 pi(c) + cit(e) = 1.5 atp(c) + 1.5 h2o(c) + 2 ac(e) + succ(e) | 0 | 0.750 |
| 1732 | 2 adp(c) + 3 h(e) + 2 pi(c) + 2 glyc(c) + cit(e) = etoh(c) + 2 atp(c) + 3 h2o(c) + 2 co2(c) + mal-L(e) + btd-RR(c) | 0 | 0.667 |
| 6 | 5 adp(c) + 5 pi(c) + 4 glyc(c) + 4 cit(e) = 5 atp(c) + 5 h2o(c) + h(e) + 10 ac(e) + 4 mal-L(e) | 0 | 0.625 |
| 2270 | 1.66667 adp(c) + 3 h(e) + 1.66667 pi(c) + 2 glyc(c) + 1.33333 cit(e) = 1.33333 etoh(c) + 1.66667 atp(c) + 3 h2o(c) + 2 co2(c) + 1.33333 mal-L(e) + actn-R(e) | 0 | 0.500 |
| 180 | 3 adp(c) + 3 pi(c) + 6 glyc(c) = 3 etoh(c) + 3 atp(c) + 6 h2o(c) + h(e) + 4 lac-L(e) | 0 | 0.500 |
| 1080, 1157, 1504 | 2 nadp(c) + 1 adp(c) + 1 pi(c) + glyc(c) + 1 cit(e) = 2 nadph(c) + 1 atp(c) + 2 co2(c) + 2 ac(e) + 1 lac-L(e) | 0 | 0.500 |
| 2053, 2214 | 4 nadp(c) + 3 adp(c) + 9 h(e) + 3 pi(c) + 2 glyc(c) + 4 cit(e) = 4 nadph(c) + 3 atp(c) + h2o(c) + 10 co2(c) + 6 ac(e) + 2 btd-RR(c) | 0 | 0.500 |
| 1314 | 2 nadp(c) + 2 adp(c) + 2 pi(c) + 2.66667 ac(e) + 5.33333 glyc(c) = 2.66667 etoh(c) + 2 nadph(c) + 2 atp(c) + 3.66667 h2o(c) + 2.33333 h(e) + co2(c) + 5 lac-L(e) | 0 | 0.375 |
| 315 | 3 adp(c) + 3 pi(c) + co2(c) + 4 ac(e) + 8 glyc(c) = 4 etoh(c) + 3 atp(c) + 7 h2o(c) + 3 h(e) + 7 lac-L(e) + mal-L(e) | 0 | 0.375 |
| 1730 | adp(c) + 3 h(e) + pi(c) + 2 ac(e) + 2.66667 glyc(c) = 2 etoh(c) + atp(c) + 3 h2o(c) + 2.66667 co2(c) + 1.33333 btd-RR(c) | 0 | 0.375 |
| 22 | etoh(c) + adp(c) + pi(c) + 2 cit(e) = atp(c) + 3 ac(e) + 2 mal-L(e) | 0 | 0.333 |
| 316 | adp(c) + pi(c) + co2(c) + ac(e) + 3 glyc(c) = etoh(c) + atp(c) + 3 h2o(c) + 2 h(e) + 2 lac-L(e) + succ(e) | 0 | 0.333 |
| 719 | 1 etoh(c) + adp(c) + 3 h(e) + 1 pi(c) + 2 cit(e) = 1 atp(c) + 2 co2(c) + 6 ac(e) | 0 | 0.333 |
| 1316 | 2 nadp(c) + 2 adp(c) + 2 pi(c) + 2 ac(e) + 6 glyc(c) = 3 etoh(c) + 2 nadph(c) + 2 atp(c) + 4 h2o(c) + 3 h(e) + co2(c) + 5 lac-L(e) | 0 | 0.333 |
| 322 | 1.66667 adp(c) + 1.66667 pi(c) + co2(c) + 4 glyc(c) + 1.33333 cit(e) = 1.33333 etoh(c) + 1.66667 atp(c) + 3 h2o(c) + 3 h(e) + 3 lac-L(e) + 2.33333 mal-L(e) | 0 | 0.313 |
| 1319 | 2.25 nadp(c) + 1.25 adp(c) + 1.25 pi(c) + 3 glyc(c) + cit(e) = 1 etoh(c) + 2.25 nadph(c) + 1.25 atp(c) + 1.125 h2o(c) + 2.625 h(e) + 1.125 co2(c) + 2.625 lac-L(e) + mal-L(e) | 0 | 0.313 |
| 1321 | 4.5 nadp(c) + 1.5 adp(c) + 1.5 pi(c) + 4 glyc(c) + cit(e) = etoh(c) + 4.5 nadph(c) + 1.5 atp(c) + 1.25 h2o(c) + 4.25 h(e) + 2.25 co2(c) + 3.25 lac-L(e) + succ(e) | 0 | 0.300 |
| 1324 | 3 nadp(c) + 2 adp(c) + 2 pi(c) + 6 glyc(c) + cit(e) = 2.5 etoh(c) + 3 nadph(c) + 2 atp(c) + 3 h2o(c) + 4.5 h(e) + 1.5 co2(c) + 4.5 lac-L(e) + mal-L(e) | 0 | 0.286 |
| 324 | 3 adp(c) + 3 pi(c) + 6 co2(c) + 10 glyc(c) + cit(e) = etoh(c) + 3 atp(c) + 11 h2o(c) + 11 h(e) + 4 lac-L(e) + 7 succ(e) | 0 | 0.273 |
| 1327 | 6 nadp(c) + 3 adp(c) + 3 pi(c) + 10 glyc(c) + cit(e) = 4 etoh(c) + 6 nadph(c) + 3 atp(c) + 5 h2o(c) + 8 h(e) + 3 co2(c) + 7 lac-L(e) + succ(e) | 0 | 0.273 |
| 72, 89, 594 | adp(c) + 2 h(e) + pi(c) + 2 glyc(c) + 2 cit(e) = atp(c) + h2o(c) + 2 co2(c) + 5 ac(e) + 2 lac-L(e) | 0 | 0.250 |
| 321 | adp(c) + pi(c) + co2(c) + 2 glyc(c) + 2 cit(e) = atp(c) + h2o(c) + 3 h(e) + 2 ac(e) + lac-L(e) + 3 mal-L(e) | 0 | 0.250 |
| 323 | 2 adp(c) + 2 pi(c) + 5 co2(c) + 7 glyc(c) + cit(e) = 2 atp(c) + 8 h2o(c) + 9 h(e) + ac(e) + 2 lac-L(e) + 6 succ(e) | 0 | 0.250 |
| 331 | 3 adp(c) + 3 pi(c) + co2(c) + 12 glyc(c) = 6 etoh(c) + 3 atp(c) + 9 h2o(c) + 7 h(e) + 7 lac-L(e) + mal-L(e) | 0 | 0.250 |
| 332 | adp(c) + pi(c) + co2(c) + 4 glyc(c) = 1.5 etoh(c) + atp(c) + 3.5 h2o(c) + 3 h(e) + 2 lac-L(e) + succ(e) | 0 | 0.250 |
| 720 | adp(c) + 1 h(e) + 1 pi(c) + 2 glyc(c) + 2 cit(e) = 1 atp(c) + 1 h2o(c) + 2 co2(c) + 8 ac(e) | 0 | 0.250 |
| 1317 | 2 nadp(c) + 2 adp(c) + 2 pi(c) + 8 glyc(c) = 4 etoh(c) + 2 nadph(c) + 2 atp(c) + 5 h2o(c) + 5 h(e) + co2(c) + 5 lac-L(e) | 0 | 0.250 |
| 1318 | 6 nadp(c) + 2 adp(c) + h2o(c) + 2 pi(c) + 4 glyc(c) + 4 cit(e) = 6 nadph(c) + 2 atp(c) + 7 h(e) + 3 co2(c) + 4 ac(e) + 3 lac-L(e) + 4 mal-L(e) | 0 | 0.250 |
| 1320 | 30 nadp(c) + 6 adp(c) + h2o(c) + 6 pi(c) + 16 glyc(c) + 8 cit(e) = 30 nadph(c) + 6 atp(c) + 27 h(e) + 15 co2(c) + 8 ac(e) + 11 lac-L(e) + 8 succ(e) | 0 | 0.250 |
| 1322 | 2 nadp(c) + adp(c) + pi(c) + 3 glyc(c) + cit(e) = 1 etoh(c) + 2 nadph(c) + atp(c) + h2o(c) + 3 h(e) + 1 co2(c) + ac(e) + 2 lac-L(e) + mal-L(e) | 0 | 0.250 |
| 1325 | 5 nadp(c) + 2 adp(c) + 2 pi(c) + 7 glyc(c) + cit(e) = 2.5 etoh(c) + 5 nadph(c) + 2 atp(c) + 3 h2o(c) + 6.5 h(e) + 2.5 co2(c) + ac(e) + 4.5 lac-L(e) + succ(e) | 0 | 0.250 |
| 1736 | adp(c) + 1 h(e) + 1 pi(c) + 4.66667 glyc(c) = 3 etoh(c) + atp(c) + 4 h2o(c) + 2.66667 co2(c) + 1.33333 btd-RR(c) | 0 | 0.214 |
| 1818 | 1.66667 adp(c) + 1.66667 pi(c) + 2 co2(c) + 7.33333 glyc(c) + cit(e) = etoh(c) + 1.66667 atp(c) + 8.33333 h2o(c) + 5.66667 h(e) + 5.66667 succ(e) + 1.33333 btd-RR(c) | 0 | 0.200 |
| 1406, 1476, 1509 | 6 nadp(c) + 2 adp(c) + 2 pi(c) + 9 glyc(c) + cit(e) = 4 etoh(c) + 6 nadph(c) + 2 atp(c) + 3 h2o(c) + 7 h(e) + 4 co2(c) + 7 lac-L(e) | 0 | 0.200 |
| 1813 | 3 adp(c) + 11 h(e) + 3 pi(c) + 11 ac(e) + 15 glyc(c) = 11 etoh(c) + 3 atp(c) + 14 h2o(c) + 13 co2(c) + mal-L(e) + 7 btd-RR(c) | 0 | 0.200 |
| 1814 | adp(c) + 2 h(e) + pi(c) + 3 ac(e) + 5 glyc(c) = 3 etoh(c) + atp(c) + 5 h2o(c) + 3 co2(c) + succ(e) + 2 btd-RR(c) | 0 | 0.200 |
| 1815 | 4 adp(c) + 4 pi(c) + co2(c) + 9 glyc(c) + 11 cit(e) = 4 atp(c) + 4 h2o(c) + 11 h(e) + 11 ac(e) + 16 mal-L(e) + 2 btd-RR(c) | 0 | 0.200 |
| 1816 | adp(c) + pi(c) + 3.42857 glyc(c) + 1.57143 cit(e) = 1.57143 etoh(c) + atp(c) + 2.57143 h2o(c) + 1.71429 co2(c) + 2.42857 mal-L(e) + 1.28571 btd-RR(c) | 0 | 0.200 |
| 1817 | 2 adp(c) + 2 pi(c) + 4.5 co2(c) + 8.5 glyc(c) + 1.5 cit(e) = 2 atp(c) + 10 h2o(c) + 9.5 h(e) + 1.5 ac(e) + 8 succ(e) + btd-RR(c) | 0 | 0.200 |
| 2079 | 1 nadp(c) + adp(c) + 3.83333 h(e) + 1 pi(c) + 3.83333 ac(e) + 5.16667 glyc(c) = 3.83333 etoh(c) + 1 nadph(c) + atp(c) + 4.33333 h2o(c) + 5.5 co2(c) + 2.5 btd-RR(c) | 0 | 0.194 |
| 2084 | 2.76923 nadp(c) + 1 adp(c) + pi(c) + 3.69231 glyc(c) + 1.76923 cit(e) = 1.76923 etoh(c) + 2.76923 nadph(c) + 1 atp(c) + 1.38462 h2o(c) + 4.61538 co2(c) + 1.76923 mal-L(e) + 1.61538 btd-RR(c) | 0 | 0.183 |
| 2081 | 1 nadp(c) + adp(c) + 3.5 h(e) + pi(c) + 3.5 ac(e) + 5.5 glyc(c) = 4 etoh(c) + 1 nadph(c) + atp(c) + 4.5 h2o(c) + 5.5 co2(c) + 2.5 btd-RR(c) | 0 | 0.182 |
| 2083 | 5 nadp(c) + 1.16667 adp(c) + 1.33333 h2o(c) + 1.16667 pi(c) + 2.83333 glyc(c) + 3.83333 cit(e) = 5 nadph(c) + 1.16667 atp(c) + 3.83333 h(e) + 4.5 co2(c) + 3.83333 ac(e) + 3.83333 mal-L(e) + btd-RR(c) | 0 | 0.175 |
| 2086 | 5.6 nadp(c) + adp(c) + pi(c) + 4.4 glyc(c) + 1.53333 cit(e) = 1.53333 etoh(c) + 5.6 nadph(c) + atp(c) + 1.26667 h2o(c) + 1.53333 h(e) + 6.26667 co2(c) + 1.53333 succ(e) + 1.73333 btd-RR(c) | 0 | 0.169 |
| 318 | etoh(c) + adp(c) + pi(c) + 1.5 co2(c) + 1.5 glyc(c) + 3.5 cit(e) = atp(c) + 4.5 h(e) + 4.5 ac(e) + 5 mal-L(e) | 0 | 0.167 |
| 320 | etoh(c) + adp(c) + pi(c) + 4 co2(c) + 4 glyc(c) + cit(e) = atp(c) + 5 h2o(c) + 7 h(e) + 2 ac(e) + 5 succ(e) | 0 | 0.167 |
| 1265 | 2 etoh(c) + 8 nadp(c) + 1 adp(c) + 5 h2o(c) + 1 pi(c) + 4 cit(e) = 8 nadph(c) + 1 atp(c) + h(e) + 8 co2(c) + 10 ac(e) | 0 | 0.167 |
| 1323 | 4 nadp(c) + adp(c) + 1 h2o(c) + pi(c) + 3 glyc(c) + 3 cit(e) = 4 nadph(c) + atp(c) + 6 h(e) + 2 co2(c) + 5 ac(e) + lac-L(e) + 3 mal-L(e) | 0 | 0.167 |
| 2338 | 2 adp(c) + 2 pi(c) + 1 co2(c) + 5 glyc(c) + 7 cit(e) = 2 atp(c) + 2 h2o(c) + 7 h(e) + 7 ac(e) + 10 mal-L(e) + actn-R(e) | 0 | 0.167 |
| 2340 | 2 adp(c) + 2 pi(c) + 6 co2(c) + 10 glyc(c) + 2 cit(e) = 2 atp(c) + 12 h2o(c) + 12 h(e) + 2 ac(e) + 10 succ(e) + actn-R(e) | 0 | 0.167 |
| 2089 | 2.4 nadp(c) + 1 adp(c) + pi(c) + 4.8 glyc(c) + 1.4 cit(e) = 2.6 etoh(c) + 2.4 nadph(c) + 1 atp(c) + 2.4 h2o(c) + 4.8 co2(c) + 1.4 mal-L(e) + 1.8 btd-RR(c) | 0 | 0.161 |
| 2085 | 11.1429 nadp(c) + 1.28571 adp(c) + h2o(c) + 1.28571 pi(c) + 5 glyc(c) + 3.28571 cit(e) = 11.1429 nadph(c) + 1.28571 atp(c) + 6.57143 h(e) + 8.71429 co2(c) + 3.28571 ac(e) + 3.28571 succ(e) + 1.57143 btd-RR(c) | 0 | 0.155 |
| 2087 | 3.33333 nadp(c) + 1 adp(c) + pi(c) + 4.33333 glyc(c) + 2.33333 cit(e) = 1.66667 etoh(c) + 3.33333 nadph(c) + 1 atp(c) + 1 h2o(c) + 2.33333 h(e) + 4.33333 co2(c) + 2.33333 ac(e) + 2.33333 mal-L(e) + 1.33333 btd-RR(c) | 0 | 0.150 |
| 333 | 1 adp(c) + 1 pi(c) + 1.5 co2(c) + 3.5 glyc(c) + 3.5 cit(e) = 1 atp(c) + h2o(c) + 6.5 h(e) + 6.5 ac(e) + 5 mal-L(e) | 0 | 0.143 |
| 334 | adp(c) + pi(c) + 4 co2(c) + 6 glyc(c) + 1 cit(e) = atp(c) + 6 h2o(c) + 9 h(e) + 4 ac(e) + 5 succ(e) | 0 | 0.143 |
| 1405, 1475, 1507 | 5 nadp(c) + adp(c) + pi(c) + 6 glyc(c) + 1 cit(e) = 2.5 etoh(c) + 5 nadph(c) + atp(c) + h2o(c) + 5.5 h(e) + 3.5 co2(c) + 1 ac(e) + 4.5 lac-L(e) | 0 | 0.143 |
| 2092 | 4 nadp(c) + 1 adp(c) + 1 pi(c) + 6 glyc(c) + cit(e) = 3 etoh(c) + 4 nadph(c) + 1 atp(c) + 3 h2o(c) + h(e) + 6 co2(c) + succ(e) + 2 btd-RR(c) | 0 | 0.143 |
| 2341 | adp(c) + pi(c) + 2 co2(c) + 6 glyc(c) + cit(e) = etoh(c) + atp(c) + 7 h2o(c) + 5 h(e) + 5 succ(e) + actn-R(e) | 0 | 0.143 |
| 2088 | 10 nadp(c) + 2 adp(c) + 3 h2o(c) + 2 pi(c) + 7 glyc(c) + 8 cit(e) = 10 nadph(c) + 2 atp(c) + 13 h(e) + 7 co2(c) + 13 ac(e) + 8 mal-L(e) + btd-RR(c) | 0 | 0.133 |
| 2090 | 5.2 nadp(c) + adp(c) + pi(c) + 6.2 glyc(c) + 1.4 cit(e) = 2.6 etoh(c) + 5.2 nadph(c) + atp(c) + 2.4 h2o(c) + 2.8 h(e) + 6.2 co2(c) + 1.4 ac(e) + 1.4 succ(e) + 1.8 btd-RR(c) | 0 | 0.132 |
| 439 | adp(c) + 1 h(e) + pi(c) + 6 glyc(c) + 2 cit(e) = 2 etoh(c) + atp(c) + 3 h2o(c) + 2 co2(c) + 8 lac-L(e) | 0 | 0.125 |
| 1015 | 2 etoh(c) + 6 nadp(c) + adp(c) + 4 h2o(c) + pi(c) + 1 glyc(c) + 5 cit(e) = 6 nadph(c) + atp(c) + 7 h(e) + 3 co2(c) + 7 ac(e) + 5 mal-L(e) | 0 | 0.125 |
| 1404, 1474, 1503 | 9 nadp(c) + adp(c) + 1.5 h2o(c) + pi(c) + 6 glyc(c) + 2 cit(e) = 2 etoh(c) + 9 nadph(c) + atp(c) + 6.5 h(e) + 6.5 co2(c) + 6.5 lac-L(e) | 0 | 0.125 |
| 1822 | adp(c) + 1 pi(c) + 8 glyc(c) = 4.5 etoh(c) + atp(c) + 6.5 h2o(c) + 1 h(e) + 3 co2(c) + 1 succ(e) + 2 btd-RR(c) | 0 | 0.125 |
| 2519 | 6 nadp(c) + 1 adp(c) + 2 h2o(c) + pi(c) + 3 glyc(c) + 5 cit(e) = 6 nadph(c) + 1 atp(c) + 5 h(e) + 5 co2(c) + 5 ac(e) + 5 mal-L(e) + 1 actn-R(e) | 0 | 0.125 |
| 1821 | 3 adp(c) + 3 pi(c) + 26 glyc(c) = 16.5 etoh(c) + 3 atp(c) + 19.5 h2o(c) + 13 co2(c) + mal-L(e) + 7 btd-RR(c) | 0 | 0.115 |
| 1021 | 1 etoh(c) + 6 nadp(c) + adp(c) + 3 h2o(c) + 1 pi(c) + 3 glyc(c) + 5 cit(e) = 6 nadph(c) + atp(c) + 9 h(e) + 3 co2(c) + 9 ac(e) + 5 mal-L(e) | 0 | 0.111 |
| 1326 | 10 nadp(c) + adp(c) + 1 h2o(c) + 1 pi(c) + 6 glyc(c) + 3 cit(e) = 10 nadph(c) + atp(c) + 12 h(e) + 5 co2(c) + 8 ac(e) + 1 lac-L(e) + 3 succ(e) | 0 | 0.111 |
| 2082 | 1 nadp(c) + adp(c) + 1 pi(c) + 9 glyc(c) = 5.75 etoh(c) + 1 nadph(c) + atp(c) + 6.25 h2o(c) + 5.5 co2(c) + 2.5 btd-RR(c) | 0 | 0.111 |
| 2524 | 12 nadp(c) + 2 adp(c) + 4 h2o(c) + 2 pi(c) + 8 glyc(c) + 10 cit(e) = 12 nadph(c) + 2 atp(c) + 16 h(e) + 8 co2(c) + 16 ac(e) + 10 mal-L(e) + actn-R(e) | 0 | 0.111 |
| 2339 | adp(c) + pi(c) + 6 glyc(c) + 3.5 cit(e) = 3.5 etoh(c) + atp(c) + 4.5 h2o(c) + 3 co2(c) + 5 mal-L(e) + 2.25 actn-R(e) | 0 | 0.105 |
| 1022 | 6 nadp(c) + adp(c) + 2 h2o(c) + pi(c) + 5 glyc(c) + 5 cit(e) = 6 nadph(c) + atp(c) + 11 h(e) + 3 co2(c) + 11 ac(e) + 5 mal-L(e) | 0 | 0.100 |
| 2521 | 16 nadp(c) + adp(c) + 2 h2o(c) + pi(c) + 6.33333 glyc(c) + 5 cit(e) = 16 nadph(c) + atp(c) + 10 h(e) + 11.6667 co2(c) + 5 ac(e) + 5 succ(e) + 1.83333 actn-R(e) | 0 | 0.088 |
| 1018 | 3.66667 etoh(c) + 16 nadp(c) + adp(c) + 5.66667 h2o(c) + pi(c) + 2.66667 glyc(c) + 5 cit(e) = 16 nadph(c) + atp(c) + 13.6667 h(e) + 8 co2(c) + 8.66667 ac(e) + 5 succ(e) | 0 | 0.088 |
| 2091 | 26 nadp(c) + 2 adp(c) + 3 h2o(c) + 2 pi(c) + 15 glyc(c) + 8 cit(e) = 26 nadph(c) + 2 atp(c) + 29 h(e) + 15 co2(c) + 21 ac(e) + 8 succ(e) + btd-RR(c) | 0 | 0.087 |
| 2523 | 6 nadp(c) + adp(c) + pi(c) + 7 glyc(c) + 5 cit(e) = 3 etoh(c) + 6 nadph(c) + atp(c) + 1 h2o(c) + 5 h(e) + 7 co2(c) + 5 ac(e) + 5 mal-L(e) + 2 actn-R(e) | 0 | 0.083 |
| 2520 | 6 nadp(c) + 1 adp(c) + pi(c) + 8 glyc(c) + 5 cit(e) = 5 etoh(c) + 6 nadph(c) + 1 atp(c) + 3 h2o(c) + 10 co2(c) + 5 mal-L(e) + 3.5 actn-R(e) | 0 | 0.077 |
| 1024 | etoh(c) + 16 nadp(c) + adp(c) + 3 h2o(c) + pi(c) + 8 glyc(c) + 5 cit(e) = 16 nadph(c) + atp(c) + 19 h(e) + 8 co2(c) + 14 ac(e) + 5 succ(e) | 0 | 0.071 |
| 2527 | 32 nadp(c) + 2 adp(c) + 4 h2o(c) + 2 pi(c) + 18 glyc(c) + 10 cit(e) = 32 nadph(c) + 2 atp(c) + 36 h(e) + 18 co2(c) + 26 ac(e) + 10 succ(e) + actn-R(e) | 0 | 0.071 |
| 1025 | 16 nadp(c) + adp(c) + 2 h2o(c) + pi(c) + 10 glyc(c) + 5 cit(e) = 16 nadph(c) + atp(c) + 21 h(e) + 8 co2(c) + 16 ac(e) + 5 succ(e) | 0 | 0.067 |
| 2522 | 16 nadp(c) + adp(c) + pi(c) + 11.3333 glyc(c) + 5 cit(e) = 5 etoh(c) + 16 nadph(c) + atp(c) + 3 h2o(c) + 5 h(e) + 16.6667 co2(c) + 5 succ(e) + 4.33333 actn-R(e) | 0 | 0.061 |
| 2525 | 6 nadp(c) + 1 adp(c) + pi(c) + 12 glyc(c) + 5 cit(e) = 8 etoh(c) + 6 nadph(c) + 1 atp(c) + 6 h2o(c) + 12 co2(c) + 5 mal-L(e) + 4.5 actn-R(e) | 0 | 0.059 |
| 2526 | 16 nadp(c) + adp(c) + pi(c) + 17 glyc(c) + 5 cit(e) = 8 etoh(c) + 16 nadph(c) + atp(c) + 6 h2o(c) + 10 h(e) + 17 co2(c) + 5 ac(e) + 5 succ(e) + 4.5 actn-R(e) | 0 | 0.045 |
| 2137 | 15 nadp(c) + 1 adp(c) + pi(c) + 19.5 glyc(c) + 3.5 cit(e) = 11 etoh(c) + 15 nadph(c) + 1 atp(c) + 4.5 h2o(c) + 3.5 h(e) + 23 co2(c) + 3.5 lac-L(e) + 6 btd-RR(c) | 0 | 0.043 |
| 2528 | 16 nadp(c) + 1 adp(c) + pi(c) + 22 glyc(c) + 5 cit(e) = 13 etoh(c) + 16 nadph(c) + 1 atp(c) + 11 h2o(c) + 5 h(e) + 22 co2(c) + 5 succ(e) + 7 actn-R(e) | 0 | 0.037 |
| 212 | o2(c) + 14 adp(c) + 20 h(e) + 14 pi(c) + 2 glyc(c) + 4 cit(e) = 14 atp(c) + 14 h2o(c) + 6 co2(c) + 6 ac(e) + 4 lac-L(e) | 0.071428571 | 2.333 |
| 299 | o2(c) + 11 adp(c) + 17 h(e) + 11 pi(c) + 2 glyc(c) + 4 cit(e) = 11 atp(c) + 11 h2o(c) + 6 co2(c) + 6 ac(e) + 4 lac-L(e) | 0.090909091 | 1.833 |
| 189 | o2(c) + 9 adp(c) + 9 h(e) + 9 pi(c) + 2 glyc(c) + 2 cit(e) = 9 atp(c) + 11 h2o(c) + 2 co2(c) + 4 ac(e) + 2 succ(e) | 0.111111111 | 2.250 |
| 188 | o2(c) + 8 adp(c) + 6 h(e) + 8 pi(c) + 2 glyc(c) + 4 cit(e) = 8 atp(c) + 8 h2o(c) + 2 co2(c) + 6 ac(e) + 4 mal-L(e) | 0.125 | 1.333 |
| 84, 586 | o2(c) + 7 adp(c) + 11 h(e) + 7 pi(c) + 2 cit(e) = 7 atp(c) + 7 h2o(c) + 4 co2(c) + 4 ac(e) | 0.142857143 | 3.500 |
| 1806, 1882 | o2(c) + 7 adp(c) + 13 h(e) + 7 pi(c) + 2 glyc(c) + 2 cit(e) = 7 atp(c) + 9 h2o(c) + 6 co2(c) + 2 ac(e) + 2 btd-RR(c) | 0.142857143 | 1.750 |
| 2293 | o2(c) + 7 adp(c) + 11 h(e) + 7 pi(c) + 2 glyc(c) + 2 cit(e) = 7 atp(c) + 9 h2o(c) + 4 co2(c) + 2 ac(e) + 2 lac-L(e) + actn-R(e) | 0.142857143 | 1.750 |
| 239 | o2(c) + 6 adp(c) + 6 h(e) + 6 pi(c) + 2 glyc(c) + 2 cit(e) = 6 atp(c) + 8 h2o(c) + 2 co2(c) + 4 ac(e) + 2 succ(e) | 0.166666667 | 1.500 |
| 179 | o2(c) + 5 adp(c) + 3 h(e) + 5 pi(c) + 2 glyc(c) = 5 atp(c) + 7 h2o(c) + 2 lac-L(e) | 0.2 | 2.500 |
| 213, 214 | o2(c) + 5 adp(c) + 6 h(e) + 5 pi(c) + glyc(c) + cit(e) = 5 atp(c) + 6 h2o(c) + 2 co2(c) + 2 ac(e) + lac-L(e) | 0.2 | 2.500 |
| 1757 | 1 o2(c) + 5 adp(c) + 7 h(e) + 5 pi(c) + 2 glyc(c) + cit(e) = 5 atp(c) + 7 h2o(c) + 3 co2(c) + ac(e) + lac-L(e) + 1 btd-RR(c) | 0.2 | 1.667 |
| 237 | o2(c) + 5 adp(c) + 3 h(e) + 5 pi(c) + 2 glyc(c) + 4 cit(e) = 5 atp(c) + 5 h2o(c) + 2 co2(c) + 6 ac(e) + 4 mal-L(e) | 0.2 | 0.833 |
| 2279 | o2(c) + 4.5 adp(c) + 5.5 h(e) + 4.5 pi(c) + 2 glyc(c) + cit(e) = 4.5 atp(c) + 7.5 h2o(c) + 2 co2(c) + ac(e) + succ(e) + actn-R(e) | 0.222222222 | 1.500 |
| 85, 587 | etoh(c) + 2 o2(c) + 8.5 adp(c) + 11.5 h(e) + 8.5 pi(c) + 2 cit(e) = 8.5 atp(c) + 9.5 h2o(c) + 4 co2(c) + 5 ac(e) | 0.235294118 | 2.833 |
| 86, 589 | o2(c) + 4.25 adp(c) + 4.75 h(e) + 4.25 pi(c) + glyc(c) + cit(e) = 4.25 atp(c) + 5.25 h2o(c) + 2 co2(c) + 3.5 ac(e) | 0.235294118 | 2.125 |
| 1735 | 1.5 o2(c) + 6 adp(c) + 6 h(e) + 6 pi(c) + 2 glyc(c) = 6 atp(c) + 9 h2o(c) + 2 co2(c) + btd-RR(c) | 0.25 | 3.000 |
| 99, 604 | o2(c) + 4 adp(c) + 8 h(e) + 4 pi(c) + 2 cit(e) = 4 atp(c) + 4 h2o(c) + 4 co2(c) + 4 ac(e) | 0.25 | 2.000 |
| 2277 | o2(c) + 4 adp(c) + 4 h(e) + 4 pi(c) + 2 glyc(c) + 2 cit(e) = 4 atp(c) + 6 h2o(c) + 2 co2(c) + 2 ac(e) + 2 mal-L(e) + actn-R(e) | 0.25 | 1.000 |
| 393 | o2(c) + 4 adp(c) + 4 pi(c) + 2 co2(c) + 6 glyc(c) + 8 cit(e) = 4 atp(c) + 4 h2o(c) + 10 h(e) + 10 ac(e) + 12 mal-L(e) | 0.25 | 0.286 |
| 395 | o2(c) + 4 adp(c) + 4 pi(c) + 8 co2(c) + 12 glyc(c) + 2 cit(e) = 4 atp(c) + 16 h2o(c) + 16 h(e) + 4 ac(e) + 12 succ(e) | 0.25 | 0.286 |
| 1357 | o2(c) + 16 nadp(c) + 4 adp(c) + 4 h2o(c) + 4 pi(c) + 10 glyc(c) + 12 cit(e) = 16 nadph(c) + 4 atp(c) + 22 h(e) + 10 co2(c) + 22 ac(e) + 12 mal-L(e) | 0.25 | 0.182 |
| 1358 | o2(c) + 40 nadp(c) + 4 adp(c) + 4 h2o(c) + 4 pi(c) + 22 glyc(c) + 12 cit(e) = 40 nadph(c) + 4 atp(c) + 46 h(e) + 22 co2(c) + 34 ac(e) + 12 succ(e) | 0.25 | 0.118 |
| 194, 199 | 2 o2(c) + 7.5 adp(c) + 6.5 h(e) + 7.5 pi(c) + 2 glyc(c) + cit(e) = 7.5 atp(c) + 10.5 h2o(c) + 2 co2(c) + 3 ac(e) + succ(e) | 0.266666667 | 2.500 |
| 1741 | 2 o2(c) + 7.5 adp(c) + 8.5 h(e) + 7.5 pi(c) + 4 glyc(c) + cit(e) = 7.5 atp(c) + 12.5 h2o(c) + 4 co2(c) + ac(e) + succ(e) + 2 btd-RR(c) | 0.266666667 | 1.500 |
| 3 | etoh(c) + o2(c) + 3.5 adp(c) + 2.5 h(e) + 3.5 pi(c) = 3.5 atp(c) + 4.5 h2o(c) + ac(e) | 0.285714286 | 3.500 |
| 2273 | 2 o2(c) + 7 adp(c) + 7 h(e) + 7 pi(c) + 2 glyc(c) = 7 atp(c) + 11 h2o(c) + 2 co2(c) + actn-R(e) | 0.285714286 | 3.500 |
| 104, 609 | etoh(c) + 2 o2(c) + 7 adp(c) + 10 h(e) + 7 pi(c) + 2 cit(e) = 7 atp(c) + 8 h2o(c) + 4 co2(c) + 5 ac(e) | 0.285714286 | 2.333 |
| 8 | 2 o2(c) + 7 adp(c) + h(e) + 7 pi(c) + 4 glyc(c) = 7 atp(c) + 11 h2o(c) + 6 ac(e) | 0.285714286 | 1.750 |
| 193, 198 | o2(c) + 3.5 adp(c) + 2.5 h(e) + 3.5 pi(c) + glyc(c) + cit(e) = 3.5 atp(c) + 4.5 h2o(c) + co2(c) + 2 ac(e) + mal-L(e) | 0.285714286 | 1.750 |
| 301, 304 | o2(c) + 3.5 adp(c) + 4.5 h(e) + 3.5 pi(c) + glyc(c) + cit(e) = 3.5 atp(c) + 4.5 h2o(c) + 2 co2(c) + 2 ac(e) + lac-L(e) | 0.285714286 | 1.750 |
| 1739 | 1 o2(c) + 3.5 adp(c) + 3.5 h(e) + 3.5 pi(c) + 2 glyc(c) + cit(e) = 3.5 atp(c) + 5.5 h2o(c) + 2 co2(c) + ac(e) + mal-L(e) + 1 btd-RR(c) | 0.285714286 | 1.167 |
| 2335, 2385 | o2(c) + 3.5 adp(c) + 7.5 h(e) + 3.5 pi(c) + 2 glyc(c) + cit(e) = etoh(c) + 3.5 atp(c) + 6.5 h2o(c) + 4 co2(c) + 1.5 actn-R(e) | 0.285714286 | 1.167 |
| 190 | 1.5 o2(c) + 5 adp(c) + 4 h(e) + 5 pi(c) + glyc(c) = 5 atp(c) + 7 h2o(c) + co2(c) + ac(e) | 0.3 | 5.000 |
| 2294 | 1.5 o2(c) + 5 adp(c) + 8 h(e) + 5 pi(c) + 3 glyc(c) + cit(e) = etoh(c) + 5 atp(c) + 9 h2o(c) + 4 co2(c) + lac-L(e) + 1.5 actn-R(e) | 0.3 | 1.250 |
| 308, 588 | 2 o2(c) + 6.5 adp(c) + 7.5 h(e) + 6.5 pi(c) + glyc(c) + cit(e) = 6.5 atp(c) + 8.5 h2o(c) + 3 co2(c) + 3 ac(e) | 0.307692308 | 3.250 |
| 1807, 1883 | 2.5 o2(c) + 8 adp(c) + 12 h(e) + 8 pi(c) + 5 glyc(c) + cit(e) = 1 etoh(c) + 8 atp(c) + 14 h2o(c) + 7 co2(c) + 3 btd-RR(c) | 0.3125 | 1.333 |
| 1758 | 3 o2(c) + 9.5 adp(c) + 12.5 h(e) + 9.5 pi(c) + 6 glyc(c) + cit(e) = 1 etoh(c) + 9.5 atp(c) + 16.5 h2o(c) + 7 co2(c) + lac-L(e) + 3 btd-RR(c) | 0.315789474 | 1.357 |
| 181, 182, 184, 185, 186 | o2(c) + 3 adp(c) + h(e) + 3 pi(c) + 2 glyc(c) = 3 atp(c) + 5 h2o(c) + 2 lac-L(e) | 0.333333333 | 1.500 |
| 187 | o2(c) + 3 adp(c) + 3 h(e) + 3 pi(c) + 2 glyc(c) = 2 etoh(c) + 3 atp(c) + 5 h2o(c) + 2 co2(c) | 0.333333333 | 1.500 |
| 197 | o2(c) + 3 adp(c) + 2 h(e) + 3 pi(c) + 2 glyc(c) = etoh(c) + 3 atp(c) + 5 h2o(c) + co2(c) + lac-L(e) | 0.333333333 | 1.500 |
| 1742 | 4 o2(c) + 12 adp(c) + 14 h(e) + 12 pi(c) + 8 glyc(c) + cit(e) = etoh(c) + 12 atp(c) + 22 h2o(c) + 8 co2(c) + succ(e) + 4 btd-RR(c) | 0.333333333 | 1.333 |
| 2280 | 2 o2(c) + 6 adp(c) + 8 h(e) + 6 pi(c) + 4 glyc(c) + cit(e) = etoh(c) + 6 atp(c) + 12 h2o(c) + 4 co2(c) + succ(e) + 2 actn-R(e) | 0.333333333 | 1.200 |
| 1352 | o2(c) + 8 nadp(c) + 2.66667 adp(c) + 1.33333 h2o(c) + 2.66667 pi(c) + 3.33333 glyc(c) + 5.33333 cit(e) = 8 nadph(c) + 2.66667 atp(c) + 7.33333 h(e) + 6 co2(c) + 7.33333 ac(e) + 5.33333 mal-L(e) | 0.374999531 | 0.308 |
| 1034 | 4.66667 etoh(c) + o2(c) + 12 nadp(c) + 2.66667 adp(c) + 6 h2o(c) + 2.66667 pi(c) + 2 glyc(c) + 9.33333 cit(e) = 12 nadph(c) + 2.66667 atp(c) + 14 h(e) + 6 co2(c) + 14 ac(e) + 9.33333 mal-L(e) | 0.374999531 | 0.167 |
| 195, 200 | 1.5 o2(c) + 4 adp(c) + 3 h(e) + 4 pi(c) + glyc(c) = 4 atp(c) + 6 h2o(c) + co2(c) + ac(e) | 0.375 | 4.000 |
| 1743 | 1.5 o2(c) + 4 adp(c) + 4 h(e) + 4 pi(c) + 2 glyc(c) = 4 atp(c) + 7 h2o(c) + 2 co2(c) + btd-RR(c) | 0.375 | 2.000 |
| 1740 | 3 o2(c) + 8 adp(c) + 9 h(e) + 8 pi(c) + 6 glyc(c) + cit(e) = 1 etoh(c) + 8 atp(c) + 15 h2o(c) + 6 co2(c) + mal-L(e) + 3 btd-RR(c) | 0.375 | 1.143 |
| 2281 | 2 o2(c) + 5 adp(c) + 5 h(e) + 5 pi(c) + 2 glyc(c) = 5 atp(c) + 9 h2o(c) + 2 co2(c) + actn-R(e) | 0.4 | 2.500 |
| 1754 | 3 o2(c) + 7.5 adp(c) + 7.5 h(e) + 7.5 pi(c) + 5 glyc(c) = 3 etoh(c) + 7.5 atp(c) + 13.5 h2o(c) + 5 co2(c) + btd-RR(c) | 0.4 | 1.500 |
| 1188, 1545 | 1 o2(c) + 2 nadp(c) + 2.5 adp(c) + 1.5 h(e) + 2.5 pi(c) + glyc(c) + 1 cit(e) = 2 nadph(c) + 2.5 atp(c) + 2.5 h2o(c) + 3 co2(c) + 3 ac(e) | 0.4 | 1.250 |
| 241 | 1.5 o2(c) + 3.5 adp(c) + 2.5 h(e) + 3.5 pi(c) + glyc(c) = 3.5 atp(c) + 5.5 h2o(c) + co2(c) + ac(e) | 0.428571429 | 3.500 |
| 2278 | 1.5 o2(c) + 3.5 adp(c) + 4.5 h(e) + 3.5 pi(c) + 3 glyc(c) + cit(e) = 1 etoh(c) + 3.5 atp(c) + 7.5 h2o(c) + 3 co2(c) + mal-L(e) + 1.5 actn-R(e) | 0.428571429 | 0.875 |
| 192, 196 | 2 o2(c) + 4.5 adp(c) + 3.5 h(e) + 4.5 pi(c) + 2 glyc(c) = etoh(c) + 4.5 atp(c) + 7.5 h2o(c) + 2 co2(c) + ac(e) | 0.444444444 | 2.250 |
| 183 | etoh(c) + 2 o2(c) + 4.5 adp(c) + 1.5 h(e) + 4.5 pi(c) + 2 glyc(c) = 4.5 atp(c) + 7.5 h2o(c) + ac(e) + 2 lac-L(e) | 0.444444444 | 1.500 |
| 250, 264 | 2 o2(c) + 4.5 adp(c) + 3.5 h(e) + 4.5 pi(c) + 2 glyc(c) + cit(e) = 4.5 atp(c) + 7.5 h2o(c) + 2 co2(c) + 3 ac(e) + succ(e) | 0.444444444 | 1.500 |
| 1753 | 2 o2(c) + 4.5 adp(c) + 4.5 h(e) + 4.5 pi(c) + 3 glyc(c) = etoh(c) + 4.5 atp(c) + 8.5 h2o(c) + 3 co2(c) + 1 btd-RR(c) | 0.444444444 | 1.500 |
| 2290 | 4 o2(c) + 9 adp(c) + 9 h(e) + 9 pi(c) + 6 glyc(c) = 4 etoh(c) + 9 atp(c) + 17 h2o(c) + 6 co2(c) + actn-R(e) | 0.444444444 | 1.500 |
| 1738 | 2 o2(c) + 4.5 adp(c) + 5.5 h(e) + 4.5 pi(c) + ac(e) + 4 glyc(c) = etoh(c) + 4.5 atp(c) + 9.5 h2o(c) + 4 co2(c) + 2 btd-RR(c) | 0.444444444 | 1.125 |
| 1355 | 2.75 o2(c) + 30 nadp(c) + 6 adp(c) + h2o(c) + 6 pi(c) + 10.5 glyc(c) + 8 cit(e) = 30 nadph(c) + 6 atp(c) + 21.5 h(e) + 20.5 co2(c) + 13.5 ac(e) + 8 succ(e) | 0.458333333 | 0.324 |
| 1744 | 2.33333 o2(c) + 5 adp(c) + 5 h(e) + 5 pi(c) + 4.66667 glyc(c) = etoh(c) + 5 atp(c) + 10.6667 h2o(c) + 4 co2(c) + 2 btd-RR(c) | 0.466666 | 1.071 |
| 191, 201, 203, 204, 205, 206, 207, 208, 209 | 1.5 o2(c) + 3 adp(c) + 2 h(e) + 3 pi(c) + glyc(c) = 3 atp(c) + 5 h2o(c) + co2(c) + ac(e) | 0.5 | 3.000 |
| 26 | etoh(c) + o2(c) + 2 adp(c) + h(e) + 2 pi(c) = 2 atp(c) + 3 h2o(c) + ac(e) | 0.5 | 2.000 |
| 1737, 1745, 1747, 1748, 1749, 1750, 1751, 1752 | 1.5 o2(c) + 3 adp(c) + 3 h(e) + 3 pi(c) + 2 glyc(c) = 3 atp(c) + 6 h2o(c) + 2 co2(c) + btd-RR(c) | 0.5 | 1.500 |
| 2289 | 3 o2(c) + 6 adp(c) + 6 h(e) + 6 pi(c) + 4 glyc(c) = 2 etoh(c) + 6 atp(c) + 12 h2o(c) + 4 co2(c) + actn-R(e) | 0.5 | 1.500 |
| 103, 608 | etoh(c) + 2 o2(c) + 4 adp(c) + 7 h(e) + 4 pi(c) + 2 cit(e) = 4 atp(c) + 5 h2o(c) + 4 co2(c) + 5 ac(e) | 0.5 | 1.333 |
| 248, 262 | o2(c) + 2 adp(c) + h(e) + 2 pi(c) + glyc(c) + cit(e) = 2 atp(c) + 3 h2o(c) + co2(c) + 2 ac(e) + mal-L(e) | 0.5 | 1.000 |
| 444 | o2(c) + 2 adp(c) + 2 pi(c) + 2 glyc(c) = 2 atp(c) + 4 h2o(c) + 2 lac-L(e) | 0.5 | 1.000 |
| 342, 357, 367, 377, 386 | o2(c) + 2 adp(c) + 2 pi(c) + 2 co2(c) + 4 glyc(c) = 2 atp(c) + 6 h2o(c) + 4 h(e) + 2 lac-L(e) + 2 succ(e) | 0.5 | 0.500 |
| 391 | o2(c) + 2 adp(c) + 2 pi(c) + 4 glyc(c) = 2 etoh(c) + 2 atp(c) + 6 h2o(c) + 2 h(e) + 2 succ(e) | 0.5 | 0.500 |
| 392 | o2(c) + 2 adp(c) + 2 pi(c) + 3 glyc(c) + cit(e) = 2 etoh(c) + 2 atp(c) + 4 h2o(c) + h(e) + co2(c) + ac(e) + 2 mal-L(e) | 0.5 | 0.500 |
| 414 | o2(c) + 2 adp(c) + 2 pi(c) + co2(c) + 4 glyc(c) = etoh(c) + 2 atp(c) + 6 h2o(c) + 3 h(e) + lac-L(e) + 2 succ(e) | 0.5 | 0.500 |
| 1331, 1335, 1339, 1342, 1345 | o2(c) + 2 nadp(c) + 2 adp(c) + 2 pi(c) + 4 glyc(c) = 1 etoh(c) + 2 nadph(c) + 2 atp(c) + 4 h2o(c) + 3 h(e) + 1 co2(c) + 3 lac-L(e) | 0.5 | 0.500 |
| 1350 | o2(c) + 2 nadp(c) + 2 adp(c) + 2 pi(c) + 4 glyc(c) = 3 etoh(c) + 2 nadph(c) + 2 atp(c) + 4 h2o(c) + 1 h(e) + 3 co2(c) + 1 lac-L(e) | 0.5 | 0.500 |
| 1377 | o2(c) + 2 nadp(c) + 2 adp(c) + 2 pi(c) + 4 glyc(c) = 2 etoh(c) + 2 nadph(c) + 2 atp(c) + 4 h2o(c) + 2 h(e) + 2 co2(c) + 2 lac-L(e) | 0.5 | 0.500 |
| 415 | 2 o2(c) + 4 adp(c) + 4 pi(c) + co2(c) + 5 glyc(c) + 5 cit(e) = 4 atp(c) + 6 h2o(c) + 7 h(e) + 7 ac(e) + 8 mal-L(e) | 0.5 | 0.400 |
| 416 | 2 o2(c) + 4 adp(c) + 4 pi(c) + 5 co2(c) + 9 glyc(c) + cit(e) = 4 atp(c) + 14 h2o(c) + 11 h(e) + 3 ac(e) + 8 succ(e) | 0.5 | 0.400 |
| 329 | o2(c) + 2 adp(c) + 2 pi(c) + 6 co2(c) + 8 glyc(c) = 2 atp(c) + 10 h2o(c) + 12 h(e) + 2 lac-L(e) + 6 succ(e) | 0.5 | 0.250 |
| 344 | 2 etoh(c) + o2(c) + 2 adp(c) + 2 pi(c) + 2 co2(c) + 2 glyc(c) + 4 cit(e) = 2 atp(c) + 2 h2o(c) + 6 h(e) + 6 ac(e) + 6 mal-L(e) | 0.5 | 0.250 |
| 1329 | o2(c) + 6 nadp(c) + 2 adp(c) + 2 pi(c) + 8 glyc(c) = 3 etoh(c) + 6 nadph(c) + 2 atp(c) + 4 h2o(c) + 9 h(e) + 3 co2(c) + 5 lac-L(e) | 0.5 | 0.250 |
| 1380 | o2(c) + 6 nadp(c) + 2 adp(c) + 2 pi(c) + 4 glyc(c) + 4 cit(e) = 6 nadph(c) + 2 atp(c) + 8 h(e) + 4 co2(c) + 8 ac(e) + 4 mal-L(e) | 0.5 | 0.250 |
| 491 | o2(c) + 2 adp(c) + 2 pi(c) + 1 co2(c) + 5 glyc(c) + 7 cit(e) = 2 atp(c) + 2 h2o(c) + 9 h(e) + 9 ac(e) + 10 mal-L(e) | 0.5 | 0.167 |
| 492 | o2(c) + 2 adp(c) + 2 pi(c) + 6 co2(c) + 10 glyc(c) + 2 cit(e) = 2 atp(c) + 12 h2o(c) + 14 h(e) + 4 ac(e) + 10 succ(e) | 0.5 | 0.167 |
| 1381 | o2(c) + 14 nadp(c) + 2 adp(c) + 2 pi(c) + 8 glyc(c) + 4 cit(e) = 14 nadph(c) + 2 atp(c) + 16 h(e) + 8 co2(c) + 12 ac(e) + 4 succ(e) | 0.5 | 0.167 |
| 1178, 1533 | o2(c) + 20 nadp(c) + 2 adp(c) + 8 h2o(c) + 2 pi(c) + 10 glyc(c) + 12 cit(e) = 20 nadph(c) + 2 atp(c) + 24 h(e) + 14 co2(c) + 24 ac(e) + 10 mal-L(e) | 0.5 | 0.091 |
| 1179, 1534 | o2(c) + 40 nadp(c) + 2 adp(c) + 8 h2o(c) + 2 pi(c) + 20 glyc(c) + 12 cit(e) = 40 nadph(c) + 2 atp(c) + 44 h(e) + 24 co2(c) + 34 ac(e) + 10 succ(e) | 0.5 | 0.063 |
| 202 | etoh(c) + 4 o2(c) + 7.5 adp(c) + 4.5 h(e) + 7.5 pi(c) + 2 glyc(c) = 7.5 atp(c) + 12.5 h2o(c) + 2 co2(c) + 3 ac(e) | 0.533333333 | 2.500 |
| 1746 | etoh(c) + 4 o2(c) + 7.5 adp(c) + 6.5 h(e) + 7.5 pi(c) + 4 glyc(c) = 7.5 atp(c) + 14.5 h2o(c) + 4 co2(c) + ac(e) + 2 btd-RR(c) | 0.533333333 | 1.500 |
| 394 | 4.5 o2(c) + 8 adp(c) + 8 pi(c) + 12 glyc(c) + cit(e) = 10 etoh(c) + 8 atp(c) + 18 h2o(c) + 6 co2(c) + 4 mal-L(e) | 0.5625 | 0.615 |
| 311, 611 | 2 o2(c) + 3.5 adp(c) + 4.5 h(e) + 3.5 pi(c) + glyc(c) + cit(e) = 3.5 atp(c) + 5.5 h2o(c) + 3 co2(c) + 3 ac(e) | 0.571428571 | 1.750 |
| 390 | 3.5 o2(c) + 6 adp(c) + h(e) + 6 pi(c) + ac(e) + 9 glyc(c) = 8 etoh(c) + 6 atp(c) + 14 h2o(c) + 5 co2(c) + 2 mal-L(e) | 0.583333333 | 0.667 |
| 399 | 1.75 o2(c) + 3 adp(c) + 3 pi(c) + 5 glyc(c) = 4.25 etoh(c) + 3 atp(c) + 7.25 h2o(c) + 2.5 co2(c) + mal-L(e) | 0.583333333 | 0.600 |
| 2130 | 3.5 o2(c) + 6 nadp(c) + 6 adp(c) + 6 pi(c) + 12 glyc(c) = 10 etoh(c) + 6 nadph(c) + 6 atp(c) + 13 h2o(c) + 12 co2(c) + btd-RR(c) | 0.583333333 | 0.500 |
| 1048 | 2.66667 etoh(c) + o2(c) + 6 nadp(c) + 1.66667 adp(c) + 2 h2o(c) + 1.66667 pi(c) + glyc(c) + 4.33333 cit(e) = 6 nadph(c) + 1.66667 atp(c) + 7 h(e) + 3 co2(c) + 7 ac(e) + 4.33333 mal-L(e) | 0.5999988 | 0.208 |
| 252, 266 | 1.5 o2(c) + 2.5 adp(c) + 1.5 h(e) + 2.5 pi(c) + glyc(c) = 2.5 atp(c) + 4.5 h2o(c) + co2(c) + ac(e) | 0.6 | 2.500 |
| 403 | 3 o2(c) + 5 adp(c) + 5 pi(c) + 4 glyc(c) = 5 atp(c) + 11 h2o(c) + h(e) + 2 ac(e) + 2 succ(e) | 0.6 | 1.250 |
| 1825 | 3 o2(c) + 5 adp(c) + h(e) + 5 pi(c) + 6 glyc(c) = 5 atp(c) + 13 h2o(c) + 2 co2(c) + 2 succ(e) + 2 btd-RR(c) | 0.6 | 0.833 |
| 1351 | 3 o2(c) + 6 nadp(c) + 5 adp(c) + 5 pi(c) + 7 glyc(c) + cit(e) = 6 etoh(c) + 6 nadph(c) + 5 atp(c) + 8 h2o(c) + h(e) + 9 co2(c) + ac(e) + mal-L(e) | 0.6 | 0.625 |
| 1482 | 3 o2(c) + 2 nadp(c) + 5 adp(c) + 5 pi(c) + 8 glyc(c) = 7 etoh(c) + 2 nadph(c) + 5 atp(c) + 11 h2o(c) + 6 co2(c) + mal-L(e) | 0.6 | 0.625 |
| 1847 | 3 o2(c) + 5 adp(c) + 5 pi(c) + 10 glyc(c) = 3 etoh(c) + 5 atp(c) + 16 h2o(c) + 5 h(e) + 5 succ(e) + btd-RR(c) | 0.6 | 0.500 |
| 1354 | 5.5 o2(c) + 12 nadp(c) + 9 adp(c) + 9 pi(c) + 13 glyc(c) + cit(e) = 11 etoh(c) + 12 nadph(c) + 9 atp(c) + 15 h2o(c) + 2 h(e) + 17 co2(c) + ac(e) + succ(e) | 0.611111111 | 0.643 |
| 1353 | 10.5 o2(c) + 18 nadp(c) + 17 adp(c) + 17 pi(c) + 24 glyc(c) + cit(e) = 22 etoh(c) + 18 nadph(c) + 17 atp(c) + 30 h2o(c) + 30 co2(c) + mal-L(e) | 0.617647059 | 0.680 |
| 1356 | 13 o2(c) + 24 nadp(c) + 21 adp(c) + 21 pi(c) + 30 glyc(c) + cit(e) = 27 etoh(c) + 24 nadph(c) + 21 atp(c) + 37 h2o(c) + h(e) + 38 co2(c) + succ(e) | 0.619047619 | 0.677 |
| 1347 | 7.5 o2(c) + 12 nadp(c) + 12 adp(c) + h(e) + 12 pi(c) + ac(e) + 17 glyc(c) = 16 etoh(c) + 12 nadph(c) + 12 atp(c) + 22 h2o(c) + 21 co2(c) | 0.625 | 0.706 |
| 1349 | o2(c) + 1.6 nadp(c) + 1.6 adp(c) + 1.6 pi(c) + 2.4 glyc(c) = 2.2 etoh(c) + 1.6 nadph(c) + 1.6 atp(c) + 3 h2o(c) + 2.8 co2(c) | 0.625 | 0.667 |
| 1348 | 2.5 o2(c) + 4 nadp(c) + 4 adp(c) + 4 pi(c) + 7 glyc(c) = 6 etoh(c) + 4 nadph(c) + 4 atp(c) + 8 h2o(c) + h(e) + 7 co2(c) + ac(e) | 0.625 | 0.571 |
| 2575 | 5 o2(c) + 8 nadp(c) + 8 adp(c) + 8 pi(c) + 16 glyc(c) = 14 etoh(c) + 8 nadph(c) + 8 atp(c) + 18 h2o(c) + 16 co2(c) + actn-R(e) | 0.625 | 0.500 |
| 1409 | 9 o2(c) + 18 nadp(c) + 14 adp(c) + 14 pi(c) + 21 glyc(c) + cit(e) = 19 etoh(c) + 18 nadph(c) + 14 atp(c) + 24 h2o(c) + h(e) + 28 co2(c) + lac-L(e) | 0.642857143 | 0.636 |
| 1478, 1527 | 13 o2(c) + 24 nadp(c) + 20 adp(c) + 20 pi(c) + 29 glyc(c) + cit(e) = 27 etoh(c) + 24 nadph(c) + 20 atp(c) + 35 h2o(c) + 39 co2(c) | 0.65 | 0.667 |
| 1330, 1333, 1338, 1341, 1344 | 1.33333 o2(c) + 2 nadp(c) + 2 adp(c) + 2 pi(c) + 2.66667 glyc(c) = 2 nadph(c) + 2 atp(c) + 3.66667 h2o(c) + 2.33333 h(e) + co2(c) + 2.33333 lac-L(e) | 0.666665 | 0.750 |
| 1375 | 1.33333 o2(c) + 2 nadp(c) + 2 adp(c) + 2 pi(c) + 2.66667 glyc(c) = 1.33333 etoh(c) + 2 nadph(c) + 2 atp(c) + 3.66667 h2o(c) + h(e) + 2.33333 co2(c) + lac-L(e) | 0.666665 | 0.750 |
| 2282 | 1.33333 o2(c) + 2 adp(c) + 2 h(e) + 2 pi(c) + 2.66667 glyc(c) = etoh(c) + 2 atp(c) + 5.66667 h2o(c) + 2 co2(c) + actn-R(e) | 0.666665 | 0.750 |
| 271 | etoh(c) + 4 o2(c) + 6 adp(c) + 3 h(e) + 6 pi(c) + 2 glyc(c) = 6 atp(c) + 11 h2o(c) + 2 co2(c) + 3 ac(e) | 0.666666667 | 2.000 |
| 4, 10, 13, 15, 17 | 2 etoh(c) + 2 o2(c) + 3 adp(c) + h(e) + 3 pi(c) = 3 atp(c) + 5 h2o(c) + 2 ac(e) | 0.666666667 | 1.500 |
| 244, 259 | 2 o2(c) + 3 adp(c) + 2 h(e) + 3 pi(c) + 2 glyc(c) = etoh(c) + 3 atp(c) + 6 h2o(c) + 2 co2(c) + ac(e) | 0.666666667 | 1.500 |
| 2275, 2283, 2284, 2285, 2286, 2287, 2288 | 2 o2(c) + 3 adp(c) + 3 h(e) + 3 pi(c) + 2 glyc(c) = 3 atp(c) + 7 h2o(c) + 2 co2(c) + actn-R(e) | 0.666666667 | 1.500 |
| 1780 | etoh(c) + 4 o2(c) + 6 adp(c) + 5 h(e) + 6 pi(c) + 4 glyc(c) = 6 atp(c) + 13 h2o(c) + 4 co2(c) + ac(e) + 2 btd-RR(c) | 0.666666667 | 1.200 |
| 12 | 1 etoh(c) + 2 o2(c) + 3 adp(c) + 3 pi(c) + 2 glyc(c) = 3 atp(c) + 6 h2o(c) + h(e) + 4 ac(e) | 0.666666667 | 1.000 |
| 225 | etoh(c) + 2 o2(c) + 3 adp(c) + 3 pi(c) + 2 glyc(c) = 3 atp(c) + 6 h2o(c) + ac(e) + 2 lac-L(e) | 0.666666667 | 1.000 |
| 9, 11, 14, 16, 18 | o2(c) + 1.5 adp(c) + 1.5 pi(c) + 2 glyc(c) = 1.5 atp(c) + 3.5 h2o(c) + 1.5 h(e) + 3 ac(e) | 0.666666667 | 0.750 |
| 341, 355, 366, 376, 385 | 2 o2(c) + 3 adp(c) + 3 pi(c) + co2(c) + 4 glyc(c) = 3 atp(c) + 7 h2o(c) + 3 h(e) + 3 lac-L(e) + mal-L(e) | 0.666666667 | 0.750 |
| 400, 411, 423, 429 | 2 o2(c) + 3 adp(c) + 3 pi(c) + 4 glyc(c) = 3 etoh(c) + 3 atp(c) + 7 h2o(c) + 2 co2(c) + mal-L(e) | 0.666666667 | 0.750 |
| 413 | 2 o2(c) + 3 adp(c) + 3 pi(c) + 4 glyc(c) = 2 etoh(c) + 3 atp(c) + 7 h2o(c) + h(e) + co2(c) + lac-L(e) + mal-L(e) | 0.666666667 | 0.750 |
| 462 | 2 o2(c) + 3 adp(c) + h(e) + 3 pi(c) + 4 glyc(c) = 2 etoh(c) + 3 atp(c) + 7 h2o(c) + 2 co2(c) + 2 lac-L(e) | 0.666666667 | 0.750 |
| 1361, 1374, 1389, 1396 | o2(c) + 1.5 nadp(c) + 1.5 adp(c) + 1.5 pi(c) + 2 glyc(c) = 1.75 etoh(c) + 1.5 nadph(c) + 1.5 atp(c) + 2.75 h2o(c) + 2.5 co2(c) | 0.666666667 | 0.750 |
| 2276 | o2(c) + 1.5 adp(c) + 2.5 h(e) + 1.5 pi(c) + ac(e) + 2 glyc(c) = etoh(c) + 1.5 atp(c) + 4.5 h2o(c) + 2 co2(c) + actn-R(e) | 0.666666667 | 0.750 |
| 412 | 2 o2(c) + 3 adp(c) + 3 pi(c) + 1 co2(c) + 5 glyc(c) = etoh(c) + 3 atp(c) + 9 h2o(c) + 4 h(e) + ac(e) + 3 succ(e) | 0.666666667 | 0.600 |
| 1378 | o2(c) + 3 nadp(c) + 1.5 adp(c) + 1.5 pi(c) + 1.5 glyc(c) + 1.5 cit(e) = 3 nadph(c) + 1.5 atp(c) + h2o(c) + 2.5 h(e) + 2.5 co2(c) + 2.5 ac(e) + 1.5 mal-L(e) | 0.666666667 | 0.500 |
| 2365 | 4 o2(c) + 6 adp(c) + 6 pi(c) + 12 glyc(c) = 4 etoh(c) + 6 atp(c) + 20 h2o(c) + 6 h(e) + 6 succ(e) + actn-R(e) | 0.666666667 | 0.500 |
| 1035 | 3.5 etoh(c) + o2(c) + 12 nadp(c) + 1.5 adp(c) + 2.5 h2o(c) + 1.5 pi(c) + 2 glyc(c) + 3.5 cit(e) = 12 nadph(c) + 1.5 atp(c) + 10.5 h(e) + 6 co2(c) + 7 ac(e) + 3.5 succ(e) | 0.666666667 | 0.167 |
| 1477, 1525 | 5.5 o2(c) + 12 nadp(c) + 8 adp(c) + 8 pi(c) + 12 glyc(c) + cit(e) = 11 etoh(c) + 12 nadph(c) + 8 atp(c) + 13 h2o(c) + h(e) + 18 co2(c) + ac(e) | 0.6875 | 0.615 |
| 2133 | 1.75 o2(c) + 2.5 nadp(c) + 2.5 adp(c) + 2.5 pi(c) + 5 glyc(c) = 3 etoh(c) + 2.5 nadph(c) + 2.5 atp(c) + 6 h2o(c) + 5 co2(c) + btd-RR(c) | 0.7 | 0.500 |
| 2097 | 2.5 o2(c) + 2 nadp(c) + 3.5 adp(c) + 1.5 h(e) + 3.5 pi(c) + 5 glyc(c) = etoh(c) + 2 nadph(c) + 3.5 atp(c) + 8.5 h2o(c) + 5 co2(c) + 2 btd-RR(c) | 0.714285714 | 0.700 |
| 1379 | 1.22222 o2(c) + 4.66667 nadp(c) + 1.66667 adp(c) + 1.66667 pi(c) + 2 glyc(c) + cit(e) = 4.66667 nadph(c) + 1.66667 atp(c) + 1.55556 h2o(c) + 3.22222 h(e) + 3.55556 co2(c) + 2.22222 ac(e) + succ(e) | 0.733330533 | 0.556 |
| 1846 | 5.5 o2(c) + 7.5 adp(c) + 7.5 pi(c) + 10 glyc(c) = 5.5 etoh(c) + 7.5 atp(c) + 18.5 h2o(c) + 5 co2(c) + 2.5 mal-L(e) + btd-RR(c) | 0.733333333 | 0.750 |
| 404, 422, 425, 428, 431, 434 | 1.5 o2(c) + 2 adp(c) + 2 pi(c) + co2(c) + 3 glyc(c) = 2 atp(c) + 6 h2o(c) + 3 h(e) + ac(e) + 2 succ(e) | 0.75 | 0.667 |
| 407 | 1.5 o2(c) + 2 adp(c) + 2 pi(c) + 2 glyc(c) + cit(e) = 2 atp(c) + 4 h2o(c) + 2 h(e) + 2 ac(e) + 2 mal-L(e) | 0.75 | 0.667 |
| 1332, 1336, 1340, 1343, 1346 | 1.5 o2(c) + 2 nadp(c) + 2 adp(c) + 2 pi(c) + 3 glyc(c) = 2 nadph(c) + 2 atp(c) + 4 h2o(c) + 3 h(e) + co2(c) + 1 ac(e) + 2 lac-L(e) | 0.75 | 0.667 |
| 1359 | 1.5 o2(c) + 3 nadp(c) + 2 adp(c) + 2 pi(c) + 3 glyc(c) = 2.5 etoh(c) + 3 nadph(c) + 2 atp(c) + 3.5 h2o(c) + h(e) + 4 co2(c) | 0.75 | 0.667 |
| 343 | etoh(c) + 1.5 o2(c) + 2 adp(c) + 2 pi(c) + 2 co2(c) + 3 glyc(c) = 2 atp(c) + 6 h2o(c) + 4 h(e) + ac(e) + lac-L(e) + 2 succ(e) | 0.75 | 0.500 |
| 1424 | 3 o2(c) + 4 nadp(c) + 4 adp(c) + 4 pi(c) + 8 glyc(c) = 7 etoh(c) + 4 nadph(c) + 4 atp(c) + 9 h2o(c) + h(e) + 8 co2(c) + ac(e) | 0.75 | 0.500 |
| 1826, 1836, 1838, 1841, 1844 | 1.5 o2(c) + 2 adp(c) + 2 pi(c) + 4 glyc(c) = 2 atp(c) + 7 h2o(c) + 2 h(e) + 2 succ(e) + btd-RR(c) | 0.75 | 0.500 |
| 1829 | 1.5 o2(c) + 2 adp(c) + 2 pi(c) + 3 glyc(c) + cit(e) = 2 atp(c) + 5 h2o(c) + h(e) + 1 co2(c) + ac(e) + 2 mal-L(e) + 1 btd-RR(c) | 0.75 | 0.500 |
| 418 | 1.5 o2(c) + 2 adp(c) + 2 pi(c) + 3 co2(c) + 5 glyc(c) = 2 atp(c) + 8 h2o(c) + 7 h(e) + ac(e) + 4 succ(e) | 0.75 | 0.400 |
| 1407 | 1.5 o2(c) + 6 nadp(c) + 2 adp(c) + 2 pi(c) + 4 glyc(c) + cit(e) = 3 etoh(c) + 6 nadph(c) + 2 atp(c) + 2 h2o(c) + 2 h(e) + 7 co2(c) + ac(e) + lac-L(e) | 0.75 | 0.400 |
| 1494 | 1.5 o2(c) + 2 nadp(c) + 2 adp(c) + 2 pi(c) + 5 glyc(c) = 4 etoh(c) + 2 nadph(c) + 2 atp(c) + 5 h2o(c) + h(e) + 4 co2(c) + lac-L(e) | 0.75 | 0.400 |
| 398 | 1.5 o2(c) + 2 adp(c) + 2 pi(c) + 5 co2(c) + 7 glyc(c) = 2 atp(c) + 10 h2o(c) + 11 h(e) + ac(e) + 6 succ(e) | 0.75 | 0.286 |
| 1820 | 1.5 o2(c) + 2 adp(c) + 2 pi(c) + 6 co2(c) + 10 glyc(c) = 2 atp(c) + 13 h2o(c) + 14 h(e) + 8 succ(e) + btd-RR(c) | 0.75 | 0.200 |
| 358 | 1.33333 etoh(c) + o2(c) + 1.33333 adp(c) + 1.33333 pi(c) + co2(c) + glyc(c) + 1.66667 cit(e) = 1.33333 atp(c) + 2 h2o(c) + 3 h(e) + 3 ac(e) + 2.66667 mal-L(e) | 0.750001875 | 0.333 |
| 1036 | 1.33333 etoh(c) + o2(c) + 4 nadp(c) + 1.33333 adp(c) + 1.33333 pi(c) + 2 glyc(c) + 2.66667 cit(e) = 4 nadph(c) + 1.33333 atp(c) + 6 h(e) + 2 co2(c) + 6 ac(e) + 2.66667 mal-L(e) | 0.750001875 | 0.222 |
| 2132 | 3.83333 o2(c) + 5 nadp(c) + 5 adp(c) + 5 pi(c) + 6.66667 glyc(c) = 3.83333 etoh(c) + 5 nadph(c) + 5 atp(c) + 10.1667 h2o(c) + 8.33333 co2(c) + btd-RR(c) | 0.766666 | 0.750 |
| 1366 | 1.21429 o2(c) + 2.57143 nadp(c) + 1.57143 adp(c) + 1.57143 pi(c) + 1.42857 glyc(c) + cit(e) = 2.57143 nadph(c) + 1.57143 atp(c) + 1.71429 h2o(c) + 2 h(e) + 2.28571 co2(c) + 2 ac(e) + mal-L(e) | 0.772729298 | 0.647 |
| 2102 | 1.21429 o2(c) + 2.57143 nadp(c) + 1.57143 adp(c) + 1.57143 pi(c) + 2.42857 glyc(c) + cit(e) = 2.57143 nadph(c) + 1.57143 atp(c) + 2.71429 h2o(c) + h(e) + 3.28571 co2(c) + ac(e) + mal-L(e) + 1 btd-RR(c) | 0.772729298 | 0.458 |
| 410 | 3.5 o2(c) + 4.5 adp(c) + 4.5 pi(c) + 5 glyc(c) = 2.5 etoh(c) + 4.5 atp(c) + 10.5 h2o(c) + h(e) + 2 co2(c) + ac(e) + 1.5 mal-L(e) | 0.777777778 | 0.900 |
| 2364 | 7 o2(c) + 9 adp(c) + 9 pi(c) + 12 glyc(c) = 7 etoh(c) + 9 atp(c) + 23 h2o(c) + 6 co2(c) + 3 mal-L(e) + actn-R(e) | 0.777777778 | 0.750 |
| 1830 | 4 o2(c) + 5 adp(c) + 5 pi(c) + 8 glyc(c) + cit(e) = 1 etoh(c) + 5 atp(c) + 14 h2o(c) + 4 co2(c) + 3 mal-L(e) + 3 btd-RR(c) | 0.8 | 0.556 |
| 345 | 5 etoh(c) + 4 o2(c) + 5 adp(c) + 5 pi(c) + 8 co2(c) + 8 glyc(c) + cit(e) = 5 atp(c) + 17 h2o(c) + 15 h(e) + 6 ac(e) + 9 succ(e) | 0.8 | 0.357 |
| 2103 | 3.42857 o2(c) + 5.14286 nadp(c) + 4.14286 adp(c) + 4.14286 pi(c) + 6.85714 glyc(c) + cit(e) = 1 etoh(c) + 5.14286 nadph(c) + 4.14286 atp(c) + 9.42857 h2o(c) + 8.57143 co2(c) + mal-L(e) + 3 btd-RR(c) | 0.827585291 | 0.527 |
| 402 | 2.5 o2(c) + 3 adp(c) + 3 pi(c) + 3 glyc(c) = etoh(c) + 3 atp(c) + 7 h2o(c) + h(e) + co2(c) + ac(e) + mal-L(e) | 0.833333333 | 1.000 |
| 1373 | 2.5 o2(c) + 3 nadp(c) + 3 adp(c) + 3 pi(c) + 3 glyc(c) = 1.5 etoh(c) + 3 nadph(c) + 3 atp(c) + 5.5 h2o(c) + h(e) + 4 co2(c) + ac(e) | 0.833333333 | 1.000 |
| 406 | 5 o2(c) + 6 adp(c) + 6 pi(c) + co2(c) + 7 glyc(c) = 6 atp(c) + 16 h2o(c) + 6 h(e) + 3 ac(e) + 3 succ(e) + 1 mal-L(e) | 0.833333333 | 0.857 |
| 1367 | 2.5 o2(c) + 6 nadp(c) + 3 adp(c) + 3 pi(c) + 3 glyc(c) + cit(e) = 6 nadph(c) + 3 atp(c) + 4 h2o(c) + 4 h(e) + 5 co2(c) + 3 ac(e) + succ(e) | 0.833333333 | 0.750 |
| 1376 | 1.25 o2(c) + 1.5 nadp(c) + 1.5 adp(c) + 1.5 pi(c) + 2 glyc(c) = 1 etoh(c) + 1.5 nadph(c) + 1.5 atp(c) + 3 h2o(c) + h(e) + 2 co2(c) + ac(e) | 0.833333333 | 0.750 |
| 1845 | 2.5 o2(c) + 3 adp(c) + 3 pi(c) + 4 glyc(c) = etoh(c) + 3 atp(c) + 8 h2o(c) + 2 co2(c) + 1 mal-L(e) + 1 btd-RR(c) | 0.833333333 | 0.750 |
| 2578 | 5 o2(c) + 6 nadp(c) + 6 adp(c) + 6 pi(c) + 8 glyc(c) = 5 etoh(c) + 6 nadph(c) + 6 atp(c) + 13 h2o(c) + 10 co2(c) + actn-R(e) | 0.833333333 | 0.750 |
| 405 | etoh(c) + 2.5 o2(c) + 3 adp(c) + 3 pi(c) + 2 co2(c) + 4 glyc(c) = 3 atp(c) + 9 h2o(c) + 5 h(e) + 2 ac(e) + 3 succ(e) | 0.833333333 | 0.600 |
| 1824 | 2.5 o2(c) + 3 adp(c) + h(e) + 3 pi(c) + ac(e) + 5 glyc(c) = etoh(c) + 3 atp(c) + 9 h2o(c) + 3 co2(c) + 1 mal-L(e) + 2 btd-RR(c) | 0.833333333 | 0.600 |
| 1828 | 5 o2(c) + 6 adp(c) + 6 pi(c) + 10 glyc(c) = 6 atp(c) + 19 h2o(c) + 3 h(e) + 2 co2(c) + 3 succ(e) + mal-L(e) + 3 btd-RR(c) | 0.833333333 | 0.600 |
| 1827 | 1 etoh(c) + 2.5 o2(c) + 3 adp(c) + 3 pi(c) + 1 co2(c) + 5 glyc(c) = 3 atp(c) + 10 h2o(c) + 4 h(e) + 1 ac(e) + 3 succ(e) + btd-RR(c) | 0.833333333 | 0.500 |
| 2104 | 2.5 o2(c) + 6 nadp(c) + 3 adp(c) + 3 pi(c) + 5 glyc(c) + cit(e) = 6 nadph(c) + 3 atp(c) + 6 h2o(c) + 2 h(e) + 7 co2(c) + ac(e) + succ(e) + 2 btd-RR(c) | 0.833333333 | 0.500 |
| 2579 | 2.5 o2(c) + 3 nadp(c) + 3 adp(c) + 3 pi(c) + 6 glyc(c) = 4 etoh(c) + 3 nadph(c) + 3 atp(c) + 8 h2o(c) + 6 co2(c) + actn-R(e) | 0.833333333 | 0.500 |
| 2347 | 2.5 o2(c) + 3 adp(c) + 3 pi(c) + 5 glyc(c) + 3 cit(e) = 3 atp(c) + 8 h2o(c) + 3 h(e) + co2(c) + 3 ac(e) + 5 mal-L(e) + 1.5 actn-R(e) | 0.833333333 | 0.375 |
| 2168 | 1.83333 o2(c) + nadp(c) + 2.16667 adp(c) + 2.16667 pi(c) + 3.66667 glyc(c) = nadph(c) + 2.16667 atp(c) + 6.5 h2o(c) + 1.16667 h(e) + 1.66667 co2(c) + 1.16667 succ(e) + 1.16667 btd-RR(c) | 0.846151006 | 0.591 |
| 2105 | 4.71429 o2(c) + 8.57143 nadp(c) + 5.57143 adp(c) + 5.57143 pi(c) + 9.42857 glyc(c) + cit(e) = etoh(c) + 8.57143 nadph(c) + 5.57143 atp(c) + 12.7143 h2o(c) + h(e) + 12.2857 co2(c) + succ(e) + 4 btd-RR(c) | 0.846154398 | 0.534 |
| 1483 | 3.66667 o2(c) + 2 nadp(c) + 4.33333 adp(c) + 4.33333 pi(c) + 5 glyc(c) = 2 nadph(c) + 4.33333 atp(c) + 10.6667 h2o(c) + 4.66667 h(e) + co2(c) + 2.33333 ac(e) + 2.33333 succ(e) | 0.846155266 | 0.867 |
| 1363 | 2.21429 o2(c) + 2.57143 nadp(c) + 2.57143 adp(c) + 2.57143 pi(c) + 2.42857 glyc(c) = etoh(c) + 2.57143 nadph(c) + 2.57143 atp(c) + 4.71429 h2o(c) + h(e) + 3.28571 co2(c) + ac(e) | 0.861112299 | 1.059 |
| 2131 | 2.21429 o2(c) + 2.57143 nadp(c) + 2.57143 adp(c) + 2.57143 pi(c) + 3.42857 glyc(c) = etoh(c) + 2.57143 nadph(c) + 2.57143 atp(c) + 5.71429 h2o(c) + 4.28571 co2(c) + 1 btd-RR(c) | 0.861112299 | 0.750 |
| 2096 | 2.21429 o2(c) + 2.57143 nadp(c) + 2.57143 adp(c) + h(e) + 2.57143 pi(c) + ac(e) + 4.42857 glyc(c) = etoh(c) + 2.57143 nadph(c) + 2.57143 atp(c) + 6.71429 h2o(c) + 5.28571 co2(c) + 2 btd-RR(c) | 0.861112299 | 0.581 |
| 1832 | 2.88889 o2(c) + 3.33333 adp(c) + 3.33333 pi(c) + 5.77778 glyc(c) = etoh(c) + 3.33333 atp(c) + 10.1111 h2o(c) + 2.88889 co2(c) + 1.11111 mal-L(e) + 2 btd-RR(c) | 0.866667867 | 0.577 |
| 1484 | 3.5 o2(c) + 2 nadp(c) + 4 adp(c) + 4 pi(c) + 5 glyc(c) = 2 nadph(c) + 4 atp(c) + 10 h2o(c) + 5 h(e) + co2(c) + 3 ac(e) + 2 succ(e) | 0.875 | 0.800 |
| 1368 | 1.75 o2(c) + 3 nadp(c) + 2 adp(c) + 2 pi(c) + 2.5 glyc(c) + cit(e) = 3 nadph(c) + 2 atp(c) + 3 h2o(c) + 3.5 h(e) + 2.5 co2(c) + 3.5 ac(e) + mal-L(e) | 0.875 | 0.571 |
| 2170 | 3.5 o2(c) + 2 nadp(c) + 4 adp(c) + 4 pi(c) + 7 glyc(c) = 2 nadph(c) + 4 atp(c) + 12 h2o(c) + 3 h(e) + 3 co2(c) + ac(e) + 2 succ(e) + 2 btd-RR(c) | 0.875 | 0.571 |
| 2098, 2113, 2118, 2123, 2128 | 1.75 o2(c) + 2 nadp(c) + 2 adp(c) + 2 pi(c) + 4 glyc(c) = etoh(c) + 2 nadph(c) + 2 atp(c) + 5.5 h2o(c) + 4 co2(c) + 1.5 btd-RR(c) | 0.875 | 0.500 |
| 2106 | 1.75 o2(c) + 3 nadp(c) + 2 adp(c) + 2 pi(c) + 3.5 glyc(c) + cit(e) = 3 nadph(c) + 2 atp(c) + 4 h2o(c) + 2.5 h(e) + 3.5 co2(c) + 2.5 ac(e) + mal-L(e) + 1 btd-RR(c) | 0.875 | 0.444 |
| 1334 | etoh(c) + 2 o2(c) + 2.25 nadp(c) + 2.25 adp(c) + 2.25 pi(c) + 2 glyc(c) = 2.25 nadph(c) + 2.25 atp(c) + 4.125 h2o(c) + 2.625 h(e) + 1.125 co2(c) + ac(e) + 1.625 lac-L(e) | 0.888888889 | 0.750 |
| 2169 | 8 o2(c) + 6 nadp(c) + 9 adp(c) + 9 pi(c) + 16 glyc(c) = 3 etoh(c) + 6 nadph(c) + 9 atp(c) + 25 h2o(c) + 14 co2(c) + mal-L(e) + 6 btd-RR(c) | 0.888888889 | 0.563 |
| 356 | 1.33333 etoh(c) + 2.66667 o2(c) + 3 adp(c) + 3 pi(c) + co2(c) + 2.66667 glyc(c) = 3 atp(c) + 7 h2o(c) + 3 h(e) + 1.33333 ac(e) + 1.66667 lac-L(e) + mal-L(e) | 0.88889 | 0.750 |
| 1049 | 5.66667 etoh(c) + 2.66667 o2(c) + 16 nadp(c) + 3 adp(c) + h2o(c) + 3 pi(c) + 2.66667 glyc(c) + 4.33333 cit(e) = 16 nadph(c) + 3 atp(c) + 14.3333 h(e) + 8 co2(c) + 10 ac(e) + 4.33333 succ(e) | 0.88889 | 0.237 |
| 2539 | 1.28571 o2(c) + 3.42857 nadp(c) + 1.42857 adp(c) + 1.42857 pi(c) + 2.57143 glyc(c) + 2 cit(e) = 3.42857 nadph(c) + 1.42857 atp(c) + 2.28571 h2o(c) + 2 h(e) + 3.71429 co2(c) + 2 ac(e) + 2 mal-L(e) + actn-R(e) | 0.8999979 | 0.312 |
| 2101 | 2.57143 o2(c) + 2.85714 nadp(c) + 2.85714 adp(c) + 2.85714 pi(c) + 5.14286 glyc(c) = etoh(c) + 2.85714 nadph(c) + 2.85714 atp(c) + 7.57143 h2o(c) + 5.42857 co2(c) + 2 btd-RR(c) | 0.9000014 | 0.556 |
| 401, 419, 421, 424, 426, 427, 430, 432, 433 | 5.5 o2(c) + 6 adp(c) + 6 pi(c) + 5 glyc(c) = 6 atp(c) + 14 h2o(c) + 3 h(e) + co2(c) + 3 ac(e) + 2 mal-L(e) | 0.916666667 | 1.200 |
| 1365 | 2.75 o2(c) + 3 nadp(c) + 3 adp(c) + 3 pi(c) + 3.5 glyc(c) = etoh(c) + 3 nadph(c) + 3 atp(c) + 6 h2o(c) + 2.5 h(e) + 3.5 co2(c) + 2.5 ac(e) | 0.916666667 | 0.857 |
| 1823, 1833, 1835, 1837, 1839, 1840, 1842, 1843 | 2.75 o2(c) + 3 adp(c) + 3 pi(c) + 4 glyc(c) = 3 atp(c) + 8.5 h2o(c) + 2 co2(c) + mal-L(e) + 1.5 btd-RR(c) | 0.916666667 | 0.750 |
| 2100 | 5.5 o2(c) + 6 nadp(c) + 6 adp(c) + 6 pi(c) + 11 glyc(c) = 2 etoh(c) + 6 nadph(c) + 6 atp(c) + 16 h2o(c) + h(e) + 11 co2(c) + ac(e) + 4 btd-RR(c) | 0.916666667 | 0.545 |
| 2363 | 3.66667 o2(c) + 4 adp(c) + 4 pi(c) + 5.33333 glyc(c) = 2 etoh(c) + 4 atp(c) + 11.3333 h2o(c) + 2.66667 co2(c) + 1.33333 mal-L(e) + actn-R(e) | 0.9166675 | 0.750 |
| 1369 | 3.75 o2(c) + 7 nadp(c) + 4 adp(c) + 4 pi(c) + 5.5 glyc(c) + cit(e) = 7 nadph(c) + 4 atp(c) + 7 h2o(c) + 7.5 h(e) + 5.5 co2(c) + 6.5 ac(e) + succ(e) | 0.9375 | 0.615 |
| 2107 | 3.75 o2(c) + 7 nadp(c) + 4 adp(c) + 4 pi(c) + 7.5 glyc(c) + cit(e) = 7 nadph(c) + 4 atp(c) + 9 h2o(c) + 5.5 h(e) + 7.5 co2(c) + 4.5 ac(e) + succ(e) + 2 btd-RR(c) | 0.9375 | 0.471 |
| 2576 | 3.28571 o2(c) + 3.42857 nadp(c) + 3.42857 adp(c) + 3.42857 pi(c) + 4.57143 glyc(c) = 2 etoh(c) + 3.42857 nadph(c) + 3.42857 atp(c) + 8.28571 h2o(c) + 5.71429 co2(c) + actn-R(e) | 0.958332483 | 0.750 |
| 1362, 1383, 1385, 1390, 1392, 1394, 1397, 1399, 1401 | 1.64286 o2(c) + 1.71429 nadp(c) + 1.71429 adp(c) + 1.71429 pi(c) + 1.28571 glyc(c) = 1.71429 nadph(c) + 1.71429 atp(c) + 3.14286 h2o(c) + h(e) + 1.85714 co2(c) + ac(e) | 0.958332604 | 1.333 |
| 2095, 2110, 2112, 2117, 2120, 2122, 2125, 2127 | 1.64286 o2(c) + 1.71429 nadp(c) + 1.71429 adp(c) + 1.71429 pi(c) + 2.28571 glyc(c) = 1.71429 nadph(c) + 1.71429 atp(c) + 4.14286 h2o(c) + 2.85714 co2(c) + btd-RR(c) | 0.958332604 | 0.750 |
| 2139 | 3.64286 o2(c) + 7.71429 nadp(c) + 3.71429 adp(c) + 3.71429 pi(c) + 7.28571 glyc(c) + cit(e) = 1 etoh(c) + 7.71429 nadph(c) + 3.71429 atp(c) + 8.14286 h2o(c) + h(e) + 10.8571 co2(c) + lac-L(e) + 3 btd-RR(c) | 0.980768868 | 0.448 |
| 245, 270, 274, 279, 282, 285, 288, 291, 294 | 3 o2(c) + 3 adp(c) + h(e) + 3 pi(c) + 2 glyc(c) = 3 atp(c) + 7 h2o(c) + 2 co2(c) + 2 ac(e) | 1 | 1.500 |
| 420 | etoh(c) + 3 o2(c) + 3 adp(c) + 3 pi(c) + 2 glyc(c) = 3 atp(c) + 7 h2o(c) + 2 h(e) + 2 ac(e) + mal-L(e) | 1 | 1.000 |
| 1364, 1386, 1388, 1391, 1393, 1395, 1398, 1400, 1402 | 1 o2(c) + 1 nadp(c) + 1 adp(c) + 1 pi(c) + 1 glyc(c) = 1 nadph(c) + 1 atp(c) + 2 h2o(c) + 1 h(e) + co2(c) + 1 ac(e) | 1 | 1.000 |
| 501 | 3 o2(c) + 3 adp(c) + 3 pi(c) + 4 glyc(c) = 2 etoh(c) + 3 atp(c) + 8 h2o(c) + h(e) + 2 co2(c) + ac(e) + mal-L(e) | 1 | 0.750 |
| 1834 | 1 etoh(c) + 3 o2(c) + 3 adp(c) + 3 pi(c) + 3 glyc(c) = 3 atp(c) + 8 h2o(c) + 1 h(e) + co2(c) + 1 ac(e) + mal-L(e) + btd-RR(c) | 1 | 0.750 |
| 1337 | 1 etoh(c) + 2 o2(c) + 2 nadp(c) + 2 adp(c) + 2 pi(c) + 2 glyc(c) = 2 nadph(c) + 2 atp(c) + 4 h2o(c) + 3 h(e) + 1 co2(c) + 2 ac(e) + lac-L(e) | 1 | 0.667 |
| 2099, 2114, 2116, 2119, 2121, 2124, 2126, 2129 | 2 o2(c) + 2 nadp(c) + 2 adp(c) + 2 pi(c) + 3 glyc(c) = 2 nadph(c) + 2 atp(c) + 5 h2o(c) + 1 h(e) + 3 co2(c) + 1 ac(e) + btd-RR(c) | 1 | 0.667 |
| 498 | etoh(c) + 4 o2(c) + 4 adp(c) + 4 pi(c) + 2 co2(c) + 6 glyc(c) = 4 atp(c) + 13 h2o(c) + 7 h(e) + 3 ac(e) + 4 succ(e) | 1 | 0.571 |
| 340, 354, 365, 371, 375, 381, 384 | etoh(c) + o2(c) + adp(c) + pi(c) + co2(c) + glyc(c) = atp(c) + 3 h2o(c) + 2 h(e) + ac(e) + succ(e) | 1 | 0.500 |
| 396 | o2(c) + adp(c) + pi(c) + 2 glyc(c) = etoh(c) + atp(c) + 3 h2o(c) + 2 h(e) + mal-L(e) | 1 | 0.500 |
| 464 | 3 o2(c) + 3 adp(c) + 3 pi(c) + 6 glyc(c) = 3 etoh(c) + 3 atp(c) + 9 h2o(c) + 2 h(e) + 2 co2(c) + 2 lac-L(e) + mal-L(e) | 1 | 0.500 |
| 502 | 2 o2(c) + 2 adp(c) + 2 pi(c) + 4 glyc(c) = etoh(c) + 2 atp(c) + 7 h2o(c) + 3 h(e) + ac(e) + 2 succ(e) | 1 | 0.500 |
| 1416, 1419, 1421, 1422, 1423 | 2 o2(c) + 2 nadp(c) + 2 adp(c) + 2 pi(c) + 4 glyc(c) = 2 nadph(c) + 2 atp(c) + 5 h2o(c) + 4 h(e) + co2(c) + 1 ac(e) + 3 lac-L(e) | 1 | 0.500 |
| 2144 | 8 o2(c) + 8 nadp(c) + 8 adp(c) + 8 pi(c) + 16 glyc(c) = 3 etoh(c) + 8 nadph(c) + 8 atp(c) + 23 h2o(c) + h(e) + 16 co2(c) + ac(e) + 6 btd-RR(c) | 1 | 0.500 |
| 2171 | 5 o2(c) + 2 nadp(c) + 5 adp(c) + 5 pi(c) + 10 glyc(c) = 2 nadph(c) + 5 atp(c) + 17 h2o(c) + 4 h(e) + 4 co2(c) + ac(e) + 3 succ(e) + 3 btd-RR(c) | 1 | 0.500 |
| 2346, 2354, 2356, 2359, 2362 | 2 o2(c) + 2 adp(c) + 2 pi(c) + 4 glyc(c) = 2 atp(c) + 8 h2o(c) + 2 h(e) + 2 succ(e) + actn-R(e) | 1 | 0.500 |
| 2538 | 2 o2(c) + 2 nadp(c) + 2 adp(c) + 2 pi(c) + 4 glyc(c) = 1 etoh(c) + 2 nadph(c) + 2 atp(c) + 6 h2o(c) + h(e) + 3 co2(c) + lac-L(e) + 1 actn-R(e) | 1 | 0.500 |
| 2669 | 4 o2(c) + 4 nadp(c) + 4 adp(c) + 4 pi(c) + 8 glyc(c) = 2 etoh(c) + 4 nadph(c) + 4 atp(c) + 12 h2o(c) + 8 co2(c) + actn-R(e) + 2 btd-RR(c) | 1 | 0.500 |
| 1850 | etoh(c) + 4 o2(c) + 4 adp(c) + 4 pi(c) + 8 glyc(c) = 4 atp(c) + 15 h2o(c) + 5 h(e) + ac(e) + 4 succ(e) + 2 btd-RR(c) | 1 | 0.444 |
| 350 | etoh(c) + 2 o2(c) + 2 adp(c) + 2 pi(c) + 2 co2(c) + 4 glyc(c) = 2 atp(c) + 7 h2o(c) + 6 h(e) + 4 ac(e) + 2 succ(e) | 1 | 0.400 |
| 475 | etoh(c) + 2 o2(c) + 2 adp(c) + 2 pi(c) + 2 co2(c) + 4 glyc(c) = 2 atp(c) + 7 h2o(c) + 5 h(e) + ac(e) + 2 lac-L(e) + 2 succ(e) | 1 | 0.400 |
| 2348 | 2 o2(c) + 2 adp(c) + 2 pi(c) + 4 glyc(c) + cit(e) = 1 etoh(c) + 2 atp(c) + 7 h2o(c) + 2 co2(c) + 2 mal-L(e) + 1.5 actn-R(e) | 1 | 0.400 |
| 347 | etoh(c) + 1 o2(c) + adp(c) + pi(c) + 1.66667 co2(c) + 1.66667 glyc(c) = atp(c) + 3.66667 h2o(c) + 3.33333 h(e) + ac(e) + 1.66667 succ(e) | 1 | 0.375 |
| 349, 362, 369, 379, 388 | 1 o2(c) + adp(c) + 1 pi(c) + 1 co2(c) + 3 glyc(c) = atp(c) + 4 h2o(c) + 4 h(e) + 3 ac(e) + 1 succ(e) | 1 | 0.333 |
| 465 | o2(c) + adp(c) + pi(c) + 3 glyc(c) = etoh(c) + atp(c) + 4 h2o(c) + 2 h(e) + lac-L(e) + succ(e) | 1 | 0.333 |
| 1050 | 1 etoh(c) + 1 o2(c) + 2 nadp(c) + 1 adp(c) + 1 pi(c) + 1 glyc(c) + cit(e) = 2 nadph(c) + 1 atp(c) + 1 h2o(c) + 3 h(e) + 1 co2(c) + 3 ac(e) + mal-L(e) | 1 | 0.333 |
| 2544 | 2 o2(c) + 4 nadp(c) + 2 adp(c) + 2 pi(c) + 4 glyc(c) + 2 cit(e) = 4 nadph(c) + 2 atp(c) + 4 h2o(c) + 4 h(e) + 4 co2(c) + 4 ac(e) + 2 mal-L(e) + actn-R(e) | 1 | 0.333 |
| 503 | o2(c) + adp(c) + pi(c) + 2 glyc(c) + 2 cit(e) = atp(c) + 2 h2o(c) + 3 h(e) + 3 ac(e) + 3 mal-L(e) | 1 | 0.250 |
| 504 | 2 o2(c) + 2 adp(c) + 2 pi(c) + 3 co2(c) + 7 glyc(c) + cit(e) = 2 atp(c) + 10 h2o(c) + 9 h(e) + 3 ac(e) + 6 succ(e) | 1 | 0.250 |
| 1037 | etoh(c) + 1 o2(c) + 4 nadp(c) + adp(c) + pi(c) + 2 glyc(c) + cit(e) = 4 nadph(c) + atp(c) + h2o(c) + 5 h(e) + 2 co2(c) + 4 ac(e) + succ(e) | 1 | 0.250 |
| 327 | etoh(c) + o2(c) + adp(c) + pi(c) + 5 co2(c) + 5 glyc(c) = atp(c) + 7 h2o(c) + 10 h(e) + ac(e) + 5 succ(e) | 1 | 0.167 |
| 2343 | 2 o2(c) + 2 adp(c) + 2 pi(c) + 8 co2(c) + 12 glyc(c) = 2 atp(c) + 16 h2o(c) + 18 h(e) + 10 succ(e) + actn-R(e) | 1 | 0.167 |
| 336 | o2(c) + 1 adp(c) + 1 pi(c) + 5 co2(c) + 7 glyc(c) = 1 atp(c) + 8 h2o(c) + 12 h(e) + 3 ac(e) + 5 succ(e) | 1 | 0.143 |
| 1191, 1549 | 1 o2(c) + 8 nadp(c) + adp(c) + 2 h2o(c) + 1 pi(c) + 4 glyc(c) + 4 cit(e) = 8 nadph(c) + atp(c) + 9 h(e) + 6 co2(c) + 9 ac(e) + 3 mal-L(e) | 1 | 0.125 |
| 1425 | 1 o2(c) + 6 nadp(c) + 1 adp(c) + 2 h2o(c) + pi(c) + 3 glyc(c) + 5 cit(e) = 6 nadph(c) + 1 atp(c) + 7 h(e) + 5 co2(c) + 7 ac(e) + 5 mal-L(e) | 1 | 0.125 |
| 1192, 1550 | 1 o2(c) + 14 nadp(c) + 1 adp(c) + 2 h2o(c) + pi(c) + 7 glyc(c) + 4 cit(e) = 14 nadph(c) + 1 atp(c) + 15 h(e) + 9 co2(c) + 12 ac(e) + 3 succ(e) | 1 | 0.091 |
| 1384 | etoh(c) + 3.44444 o2(c) + 3.33333 nadp(c) + 3.33333 adp(c) + 3.33333 pi(c) + 2 glyc(c) = 3.33333 nadph(c) + 3.33333 atp(c) + 6.11111 h2o(c) + 2.44444 h(e) + 3.11111 co2(c) + 2.44444 ac(e) | 1.033333033 | 1.111 |
| 2111 | etoh(c) + 3.44444 o2(c) + 3.33333 nadp(c) + 3.33333 adp(c) + 3.33333 pi(c) + 3.44444 glyc(c) = 3.33333 nadph(c) + 3.33333 atp(c) + 7.55556 h2o(c) + h(e) + 4.55556 co2(c) + ac(e) + 1.44444 btd-RR(c) | 1.033333033 | 0.750 |
| 2541 | 1.35714 o2(c) + 4.28571 nadp(c) + 1.28571 adp(c) + 1.28571 pi(c) + 2.71429 glyc(c) + cit(e) = 4.28571 nadph(c) + 1.28571 atp(c) + 2.85714 h2o(c) + 2 h(e) + 4.14286 co2(c) + ac(e) + succ(e) + actn-R(e) | 1.055556852 | 0.346 |
| 2165, 2194 | 3.07143 o2(c) + 6.85714 nadp(c) + 2.85714 adp(c) + 2.85714 pi(c) + 6.14286 glyc(c) + cit(e) = 1 etoh(c) + 6.85714 nadph(c) + 2.85714 atp(c) + 6.57143 h2o(c) + 10.4286 co2(c) + 3 btd-RR(c) | 1.075001575 | 0.400 |
| 408 | 6.5 o2(c) + 6 adp(c) + 6 pi(c) + co2(c) + 7 glyc(c) = 6 atp(c) + 16 h2o(c) + 9 h(e) + 3 ac(e) + 4 mal-L(e) | 1.083333333 | 0.857 |
| 1831 | 3.25 o2(c) + 3 adp(c) + 3 pi(c) + 5 glyc(c) = 3 atp(c) + 9.5 h2o(c) + 3 h(e) + co2(c) + 2 mal-L(e) + 1.5 btd-RR(c) | 1.083333333 | 0.600 |
| 2540 | 1.71429 o2(c) + 2.57143 nadp(c) + 1.57143 adp(c) + 1.57143 pi(c) + 3.42857 glyc(c) + cit(e) = 1 etoh(c) + 2.57143 nadph(c) + 1.57143 atp(c) + 4.71429 h2o(c) + 4.28571 co2(c) + mal-L(e) + 1.5 actn-R(e) | 1.090910826 | 0.355 |
| 1387 | etoh(c) + 2.75 o2(c) + 2.5 nadp(c) + 2.5 adp(c) + 2.5 pi(c) + 2 glyc(c) = 2.5 nadph(c) + 2.5 atp(c) + 5 h2o(c) + 3 h(e) + 2 co2(c) + 3 ac(e) | 1.1 | 0.833 |
| 2115 | 1.33333 etoh(c) + 3.66667 o2(c) + 3.33333 nadp(c) + 3.33333 adp(c) + 3.33333 pi(c) + 3.66667 glyc(c) = 3.33333 nadph(c) + 3.33333 atp(c) + 7.66667 h2o(c) + 3 h(e) + 3.66667 co2(c) + 3 ac(e) + btd-RR(c) | 1.1000021 | 0.667 |
| 2577 | 1.8 o2(c) + 1.6 nadp(c) + 1.6 adp(c) + 1.6 pi(c) + 3.2 glyc(c) = 1.2 etoh(c) + 1.6 nadph(c) + 1.6 atp(c) + 5.2 h2o(c) + 3.2 co2(c) + actn-R(e) | 1.125 | 0.500 |
| 2547 | 2.25 o2(c) + 5 nadp(c) + 2 adp(c) + 2 pi(c) + 4.5 glyc(c) + cit(e) = 5 nadph(c) + 2 atp(c) + 5 h2o(c) + 4.5 h(e) + 4.5 co2(c) + 3.5 ac(e) + succ(e) + actn-R(e) | 1.125 | 0.364 |
| 2542 | 2.42857 o2(c) + 5.14286 nadp(c) + 2.14286 adp(c) + 2.14286 pi(c) + 4.85714 glyc(c) + cit(e) = etoh(c) + 5.14286 nadph(c) + 2.14286 atp(c) + 6.42857 h2o(c) + h(e) + 6.57143 co2(c) + succ(e) + 2 actn-R(e) | 1.133331156 | 0.366 |
| 1051 | 2.33333 etoh(c) + 2.66667 o2(c) + 5.33333 nadp(c) + 2.33333 adp(c) + 2.33333 pi(c) + 2.66667 glyc(c) + cit(e) = 5.33333 nadph(c) + 2.33333 atp(c) + 3.66667 h2o(c) + 7 h(e) + 2.66667 co2(c) + 6 ac(e) + succ(e) | 1.142860204 | 0.389 |
| 1441 | 2.33333 o2(c) + 2 nadp(c) + 2 adp(c) + 2 pi(c) + 2.66667 glyc(c) = 1.33333 etoh(c) + 2 nadph(c) + 2 atp(c) + 4.66667 h2o(c) + h(e) + 3.33333 co2(c) + ac(e) | 1.166665 | 0.750 |
| 338, 353, 364, 370, 372, 374, 380, 383, 389 | 3 etoh(c) + 3.5 o2(c) + 3 adp(c) + 3 pi(c) + co2(c) + glyc(c) = 3 atp(c) + 7 h2o(c) + 3 h(e) + 3 ac(e) + mal-L(e) | 1.166666667 | 0.750 |
| 495 | 3.5 o2(c) + 3 adp(c) + 3 pi(c) + 4 glyc(c) = 1.5 etoh(c) + 3 atp(c) + 8.5 h2o(c) + 1.5 h(e) + 2 co2(c) + 1.5 ac(e) + mal-L(e) | 1.166666667 | 0.750 |
| 2344, 2351, 2353, 2355, 2357, 2358, 2360, 2361 | 3.5 o2(c) + 3 adp(c) + 3 pi(c) + 4 glyc(c) = 3 atp(c) + 10 h2o(c) + 2 co2(c) + mal-L(e) + 1.5 actn-R(e) | 1.166666667 | 0.750 |
| 2352 | etoh(c) + 7 o2(c) + 6 adp(c) + 6 pi(c) + 7 glyc(c) = 6 atp(c) + 19 h2o(c) + h(e) + 3 co2(c) + ac(e) + 2 mal-L(e) + 2.5 actn-R(e) | 1.166666667 | 0.750 |
| 361 | 1.75 etoh(c) + 3.5 o2(c) + 3 adp(c) + 3 pi(c) + co2(c) + 3.5 glyc(c) = 3 atp(c) + 8.25 h2o(c) + 5.5 h(e) + 5.5 ac(e) + mal-L(e) | 1.166666667 | 0.571 |
| 348, 360, 368, 378, 387 | 3.5 o2(c) + 3 adp(c) + 3 pi(c) + co2(c) + 7 glyc(c) = 3 atp(c) + 10 h2o(c) + 9 h(e) + 9 ac(e) + mal-L(e) | 1.166666667 | 0.429 |
| 2345 | 3.5 o2(c) + 3 adp(c) + 3 h(e) + 3 pi(c) + 3 ac(e) + 7 glyc(c) = 3 etoh(c) + 3 atp(c) + 13 h2o(c) + 5 co2(c) + mal-L(e) + 3 actn-R(e) | 1.166666667 | 0.429 |
| 2350 | 3.5 o2(c) + 3 adp(c) + 3 pi(c) + 7 glyc(c) = 2.25 etoh(c) + 3 atp(c) + 12.25 h2o(c) + 3.5 co2(c) + mal-L(e) + 2.25 actn-R(e) | 1.166666667 | 0.429 |
| 2543 | 3.5 o2(c) + 4 nadp(c) + 3 adp(c) + 3 pi(c) + 7 glyc(c) + cit(e) = 2 etoh(c) + 4 nadph(c) + 3 atp(c) + 10 h2o(c) + h(e) + 7 co2(c) + ac(e) + mal-L(e) + 2.5 actn-R(e) | 1.166666667 | 0.375 |
| 1495 | 1.16667 o2(c) + 1 nadp(c) + adp(c) + 1 pi(c) + 2.33333 glyc(c) = 1.16667 etoh(c) + 1 nadph(c) + atp(c) + 2.83333 h2o(c) + 1 h(e) + 1.66667 co2(c) + 1 lac-L(e) | 1.16667 | 0.429 |
| 2545 | 6 o2(c) + 6 nadp(c) + 5 adp(c) + 5 pi(c) + 12 glyc(c) + cit(e) = 4 etoh(c) + 6 nadph(c) + 5 atp(c) + 18 h2o(c) + 12 co2(c) + mal-L(e) + 4.5 actn-R(e) | 1.2 | 0.385 |
| 2546 | 8.5 o2(c) + 10 nadp(c) + 7 adp(c) + 7 pi(c) + 17 glyc(c) + cit(e) = 5 etoh(c) + 10 nadph(c) + 7 atp(c) + 25 h2o(c) + 2 h(e) + 17 co2(c) + ac(e) + succ(e) + 6 actn-R(e) | 1.214285714 | 0.389 |
| 2548 | 11 o2(c) + 12 nadp(c) + 9 adp(c) + 9 pi(c) + 22 glyc(c) + cit(e) = 7 etoh(c) + 12 nadph(c) + 9 atp(c) + 33 h2o(c) + h(e) + 22 co2(c) + succ(e) + 8 actn-R(e) | 1.222222222 | 0.391 |
| 2537 | 1.42857 o2(c) + 1.14286 nadp(c) + 1.14286 adp(c) + 1.14286 pi(c) + 2.85714 glyc(c) = etoh(c) + 1.14286 nadph(c) + 1.14286 atp(c) + 4.42857 h2o(c) + 2.57143 co2(c) + actn-R(e) | 1.249995625 | 0.400 |
| 2138 | 1.42857 o2(c) + 5.14286 nadp(c) + 1.14286 adp(c) + 1.14286 pi(c) + 2.85714 glyc(c) + cit(e) = 5.14286 nadph(c) + 1.14286 atp(c) + 1.42857 h2o(c) + 2 h(e) + 5.57143 co2(c) + ac(e) + lac-L(e) + 1 btd-RR(c) | 1.249995625 | 0.296 |
| 1410 | 3.33333 o2(c) + 12 nadp(c) + 2.66667 adp(c) + 2.66667 pi(c) + 4.33333 glyc(c) + 2.33333 cit(e) = 12 nadph(c) + 2.66667 atp(c) + h2o(c) + 7 h(e) + 10.6667 co2(c) + 4.66667 ac(e) + 2.33333 lac-L(e) | 1.249997188 | 0.400 |
| 1370 | 1.78571 o2(c) + 3.42857 nadp(c) + 1.42857 adp(c) + 1.42857 pi(c) + 1.57143 glyc(c) = 3.42857 nadph(c) + 1.42857 atp(c) + 2.28571 h2o(c) + 3 h(e) + 2.71429 co2(c) + ac(e) | 1.24999825 | 0.909 |
| 2108 | 1.78571 o2(c) + 3.42857 nadp(c) + 1.42857 adp(c) + 1.42857 pi(c) + 2.57143 glyc(c) = 3.42857 nadph(c) + 1.42857 atp(c) + 3.28571 h2o(c) + 2 h(e) + 3.71429 co2(c) + btd-RR(c) | 1.24999825 | 0.556 |
| 1431 | 2.14286 o2(c) + 1.71429 nadp(c) + 1.71429 adp(c) + 1.71429 pi(c) + 2.28571 glyc(c) = etoh(c) + 1.71429 nadph(c) + 1.71429 atp(c) + 4.14286 h2o(c) + h(e) + 2.85714 co2(c) + ac(e) | 1.249998542 | 0.750 |
| 2531, 2552, 2554, 2560, 2563, 2566, 2569, 2572 | 2.14286 o2(c) + 1.71429 nadp(c) + 1.71429 adp(c) + 1.71429 pi(c) + 2.28571 glyc(c) = 1.71429 nadph(c) + 1.71429 atp(c) + 5.14286 h2o(c) + 2.85714 co2(c) + actn-R(e) | 1.249998542 | 0.750 |
| 1030, 1042, 1055, 1059, 1061, 1064, 1068, 1071, 1075 | 7 etoh(c) + 7.5 o2(c) + 6 nadp(c) + 6 adp(c) + 6 pi(c) + glyc(c) = 6 nadph(c) + 6 atp(c) + 11 h2o(c) + 7 h(e) + 3 co2(c) + 7 ac(e) | 1.25 | 0.750 |
| 2553 | etoh(c) + 15 o2(c) + 12 nadp(c) + 12 adp(c) + 12 pi(c) + 15 glyc(c) = 12 nadph(c) + 12 atp(c) + 35 h2o(c) + h(e) + 19 co2(c) + ac(e) + 6.5 actn-R(e) | 1.25 | 0.750 |
| 1032, 1045, 1057, 1060, 1062, 1066, 1069, 1073, 1076 | 2 etoh(c) + 2.5 o2(c) + 2 nadp(c) + 2 adp(c) + 2 pi(c) + 1 glyc(c) = 2 nadph(c) + 2 atp(c) + 4 h2o(c) + 3 h(e) + co2(c) + 3 ac(e) | 1.25 | 0.667 |
| 1371 | 1.25 o2(c) + 2 nadp(c) + adp(c) + pi(c) + 1.5 glyc(c) = 2 nadph(c) + atp(c) + 2 h2o(c) + 2.5 h(e) + 1.5 co2(c) + 1.5 ac(e) | 1.25 | 0.667 |
| 2535, 2556, 2559, 2562, 2565, 2568, 2571, 2574 | 2.5 o2(c) + 2 nadp(c) + 2 adp(c) + 2 pi(c) + 3 glyc(c) = 2 nadph(c) + 2 atp(c) + 6 h2o(c) + 1 h(e) + 3 co2(c) + 1 ac(e) + actn-R(e) | 1.25 | 0.667 |
| 2557 | etoh(c) + 5 o2(c) + 4 nadp(c) + 4 adp(c) + 4 pi(c) + 5 glyc(c) = 4 nadph(c) + 4 atp(c) + 11 h2o(c) + 3 h(e) + 5 co2(c) + 3 ac(e) + 1.5 actn-R(e) | 1.25 | 0.667 |
| 1047 | 1.25 etoh(c) + 2.5 o2(c) + 2 nadp(c) + 2 adp(c) + 2 pi(c) + 2.5 glyc(c) = 2 nadph(c) + 2 atp(c) + 4.75 h2o(c) + 4.5 h(e) + co2(c) + 4.5 ac(e) | 1.25 | 0.533 |
| 2109 | 2.5 o2(c) + 4 nadp(c) + 2 adp(c) + 2 pi(c) + 4 glyc(c) = 4 nadph(c) + 2 atp(c) + 5 h2o(c) + 4 h(e) + 4 co2(c) + 2 ac(e) + btd-RR(c) | 1.25 | 0.500 |
| 2534, 2555, 2558, 2561, 2564, 2567, 2570, 2573 | 2.5 o2(c) + 2 nadp(c) + 2 adp(c) + 2 pi(c) + 4 glyc(c) = etoh(c) + 2 nadph(c) + 2 atp(c) + 7 h2o(c) + 4 co2(c) + 1.5 actn-R(e) | 1.25 | 0.500 |
| 1033, 1046, 1058, 1067, 1074 | 2.5 o2(c) + 2 nadp(c) + 2 adp(c) + 2 pi(c) + 5 glyc(c) = 2 nadph(c) + 2 atp(c) + 6 h2o(c) + 7 h(e) + co2(c) + 7 ac(e) | 1.25 | 0.400 |
| 1411 | 2.5 o2(c) + 6 nadp(c) + 2 adp(c) + 2 pi(c) + 4 glyc(c) + cit(e) = 6 nadph(c) + 2 atp(c) + 3 h2o(c) + 6 h(e) + 5 co2(c) + 5 ac(e) + lac-L(e) | 1.25 | 0.400 |
| 2146, 2172, 2236, 2244, 2254 | 2.5 o2(c) + 2 nadp(c) + 2 adp(c) + 2 pi(c) + 5 glyc(c) = etoh(c) + 2 nadph(c) + 2 atp(c) + 7 h2o(c) + 5 co2(c) + 2 btd-RR(c) | 1.25 | 0.400 |
| 2532 | 1.25 o2(c) + 1 nadp(c) + adp(c) + 1.16667 h(e) + pi(c) + 1.16667 ac(e) + 2.5 glyc(c) = 1.16667 etoh(c) + 1 nadph(c) + atp(c) + 4.16667 h2o(c) + 2.83333 co2(c) + 1.16667 actn-R(e) | 1.25 | 0.400 |
| 2536 | 2.5 o2(c) + 2 nadp(c) + 2 adp(c) + h(e) + 2 pi(c) + ac(e) + 5 glyc(c) = 2 etoh(c) + 2 nadph(c) + 2 atp(c) + 8 h2o(c) + 5 co2(c) + 2 actn-R(e) | 1.25 | 0.400 |
| 2140 | 2.5 o2(c) + 6 nadp(c) + 2 adp(c) + 2 pi(c) + 5 glyc(c) + cit(e) = 6 nadph(c) + 2 atp(c) + 4 h2o(c) + 5 h(e) + 6 co2(c) + 4 ac(e) + lac-L(e) + 1 btd-RR(c) | 1.25 | 0.333 |
| 2588 | 10.5 o2(c) + 12 nadp(c) + 8 adp(c) + 8 pi(c) + 21 glyc(c) + cit(e) = 7 etoh(c) + 12 nadph(c) + 8 atp(c) + 30 h2o(c) + h(e) + 22 co2(c) + lac-L(e) + 7.5 actn-R(e) | 1.3125 | 0.364 |
| 269 | etoh(c) + 4 o2(c) + 3 adp(c) + 3 pi(c) + 2 glyc(c) = 3 atp(c) + 8 h2o(c) + 2 co2(c) + 3 ac(e) | 1.333333333 | 1.000 |
| 1451 | etoh(c) + 3.55556 o2(c) + 2.66667 nadp(c) + 2.66667 adp(c) + 2.66667 pi(c) + 2 glyc(c) = 2.66667 nadph(c) + 2.66667 atp(c) + 5.88889 h2o(c) + 2.55556 h(e) + 2.88889 co2(c) + 2.55556 ac(e) | 1.333333333 | 0.889 |
| 509 | 2.5 etoh(c) + 8 o2(c) + 6 adp(c) + 6 pi(c) + 5 glyc(c) = 6 atp(c) + 16.5 h2o(c) + 5.5 h(e) + co2(c) + 5.5 ac(e) + 2 mal-L(e) | 1.333333333 | 0.800 |
| 2596 | etoh(c) + 16 o2(c) + 12 nadp(c) + 12 adp(c) + 12 pi(c) + 16 glyc(c) = 12 nadph(c) + 12 atp(c) + 37 h2o(c) + h(e) + 20 co2(c) + ac(e) + 7 actn-R(e) | 1.333333333 | 0.706 |
| 2366 | etoh(c) + 8 o2(c) + 6 adp(c) + 6 pi(c) + 8 glyc(c) = 6 atp(c) + 21 h2o(c) + h(e) + 4 co2(c) + ac(e) + 2 mal-L(e) + 3 actn-R(e) | 1.333333333 | 0.667 |
| 2150 | etoh(c) + 3.55556 o2(c) + 2.66667 nadp(c) + 2.66667 adp(c) + 2.66667 pi(c) + 3.55556 glyc(c) = 2.66667 nadph(c) + 2.66667 atp(c) + 7.44444 h2o(c) + h(e) + 4.44444 co2(c) + ac(e) + 1.55556 btd-RR(c) | 1.333333333 | 0.585 |
| 1853 | 1.25 etoh(c) + 4 o2(c) + 3 adp(c) + 3 pi(c) + 4 glyc(c) = 3 atp(c) + 9.75 h2o(c) + 1.25 h(e) + 2 co2(c) + 1.25 ac(e) + mal-L(e) + 1.5 btd-RR(c) | 1.333333333 | 0.571 |
| 47 | etoh(c) + 2 o2(c) + 1.5 adp(c) + 1.5 pi(c) + 2 glyc(c) = 1.5 atp(c) + 4.5 h2o(c) + 2.5 h(e) + 4 ac(e) | 1.333333333 | 0.500 |
| 455 | etoh(c) + 2 o2(c) + 1.5 adp(c) + 1.5 pi(c) + 2 glyc(c) = 1.5 atp(c) + 4.5 h2o(c) + 1.5 h(e) + ac(e) + 2 lac-L(e) | 1.333333333 | 0.500 |
| 483 | 2 etoh(c) + 4 o2(c) + 3 adp(c) + 3 pi(c) + co2(c) + 4 glyc(c) = 3 atp(c) + 9 h2o(c) + 5 h(e) + 2 ac(e) + 3 lac-L(e) + mal-L(e) | 1.333333333 | 0.500 |
| 499 | 4 o2(c) + 3 adp(c) + 3 pi(c) + 5 glyc(c) + 3 cit(e) = 3 atp(c) + 8 h2o(c) + 6 h(e) + co2(c) + 6 ac(e) + 5 mal-L(e) | 1.333333333 | 0.375 |
| 2586 | 8 o2(c) + 10 nadp(c) + 6 adp(c) + 6 pi(c) + 16 glyc(c) + cit(e) = 5 etoh(c) + 10 nadph(c) + 6 atp(c) + 22 h2o(c) + 2 h(e) + 17 co2(c) + ac(e) + lac-L(e) + 5.5 actn-R(e) | 1.333333333 | 0.353 |
| 1417 | 1.33333 etoh(c) + 2.66667 o2(c) + 2 nadp(c) + 2 adp(c) + 2 pi(c) + 2.66667 glyc(c) = 2 nadph(c) + 2 atp(c) + 5 h2o(c) + 3.66667 h(e) + co2(c) + 1.33333 ac(e) + 2.33333 lac-L(e) | 1.333335 | 0.500 |
| 2145, 2152, 2154, 2155, 2156, 2157, 2158, 2159 | 2.75 o2(c) + 2 nadp(c) + 2 adp(c) + 2 pi(c) + 4 glyc(c) = 2 nadph(c) + 2 atp(c) + 6.5 h2o(c) + h(e) + 4 co2(c) + ac(e) + 1.5 btd-RR(c) | 1.375 | 0.500 |
| 2550 | 2.75 o2(c) + 3 nadp(c) + 2 adp(c) + 2 pi(c) + 5 glyc(c) = 1.5 etoh(c) + 3 nadph(c) + 2 atp(c) + 7.5 h2o(c) + h(e) + 5 co2(c) + 1.75 actn-R(e) | 1.375 | 0.400 |
| 1195, 1553 | 2.33333 etoh(c) + 4.66667 o2(c) + 7.33333 nadp(c) + 3.33333 adp(c) + 3.33333 pi(c) + 3.66667 glyc(c) + cit(e) = 7.33333 nadph(c) + 3.33333 atp(c) + 5.66667 h2o(c) + 8 h(e) + 5.66667 co2(c) + 8 ac(e) | 1.4000024 | 0.476 |
| 2612, 2633 | 8.5 o2(c) + 10 nadp(c) + 6 adp(c) + 6 pi(c) + 17 glyc(c) + cit(e) = 6 etoh(c) + 10 nadph(c) + 6 atp(c) + 24 h2o(c) + 19 co2(c) + 6.5 actn-R(e) | 1.416666667 | 0.333 |
| 1455 | etoh(c) + 3 o2(c) + 2 nadp(c) + 2 adp(c) + 2 pi(c) + 2 glyc(c) = 2 nadph(c) + 2 atp(c) + 5 h2o(c) + 3 h(e) + 2 co2(c) + 3 ac(e) | 1.5 | 0.667 |
| 2599 | etoh(c) + 6 o2(c) + 4 nadp(c) + 4 adp(c) + 4 pi(c) + 6 glyc(c) = 4 nadph(c) + 4 atp(c) + 13 h2o(c) + 3 h(e) + 6 co2(c) + 3 ac(e) + 2 actn-R(e) | 1.5 | 0.571 |
| 359 | etoh(c) + 1.5 o2(c) + adp(c) + pi(c) + co2(c) + glyc(c) = atp(c) + 3 h2o(c) + 3 h(e) + ac(e) + mal-L(e) | 1.5 | 0.500 |
| 497, 512, 515, 518, 520, 523, 544, 660, 845, 986 | 1.5 o2(c) + adp(c) + pi(c) + 2 glyc(c) = atp(c) + 4 h2o(c) + 2 h(e) + ac(e) + succ(e) | 1.5 | 0.500 |
| 1442 | 1.5 o2(c) + 1 nadp(c) + adp(c) + pi(c) + 2 glyc(c) = 1 etoh(c) + 1 nadph(c) + atp(c) + 3 h2o(c) + 1 h(e) + 2 co2(c) + 1 ac(e) | 1.5 | 0.500 |
| 2153 | 1 etoh(c) + 3 o2(c) + 2 nadp(c) + 2 adp(c) + 2 pi(c) + 3 glyc(c) = 2 nadph(c) + 2 atp(c) + 6 h2o(c) + 2 h(e) + 3 co2(c) + 2 ac(e) + btd-RR(c) | 1.5 | 0.500 |
| 2349 | 3 o2(c) + 2 adp(c) + 2 pi(c) + 4 glyc(c) = 2 atp(c) + 8 h2o(c) + 4 h(e) + 2 mal-L(e) + actn-R(e) | 1.5 | 0.500 |
| 1420 | 1.5 etoh(c) + 3 o2(c) + 2 nadp(c) + 2 adp(c) + 2 pi(c) + 3 glyc(c) = 2 nadph(c) + 2 atp(c) + 5.5 h2o(c) + 4.5 h(e) + co2(c) + 2.5 ac(e) + 2 lac-L(e) | 1.5 | 0.444 |
| 1090, 1167, 1517 | 2 etoh(c) + 3 o2(c) + 6 nadp(c) + 2 adp(c) + 2 pi(c) + 3 glyc(c) + cit(e) = 6 nadph(c) + 2 atp(c) + 3 h2o(c) + 7 h(e) + 4 co2(c) + 6 ac(e) + lac-L(e) | 1.5 | 0.333 |
| 1851, 1862, 1910, 2013 | 1.5 o2(c) + adp(c) + pi(c) + 3 glyc(c) = atp(c) + 5 h2o(c) + h(e) + 1 co2(c) + succ(e) + 1 btd-RR(c) | 1.5 | 0.333 |
| 1966 | 1.5 o2(c) + adp(c) + 1 pi(c) + 3 glyc(c) = 1 atp(c) + 5 h2o(c) + 1 h(e) + 1 co2(c) + 1 succ(e) + 1 btd-RR(c) | 1.5 | 0.333 |
| 2611, 2632 | 6 o2(c) + 8 nadp(c) + 4 adp(c) + 4 pi(c) + 12 glyc(c) + cit(e) = 4 etoh(c) + 8 nadph(c) + 4 atp(c) + 16 h2o(c) + h(e) + 14 co2(c) + ac(e) + 4.5 actn-R(e) | 1.5 | 0.308 |
| 480 | etoh(c) + 3 o2(c) + 2 adp(c) + 2 pi(c) + 2 co2(c) + 6 glyc(c) = 2 atp(c) + 9 h2o(c) + 9 h(e) + 7 ac(e) + 2 succ(e) | 1.5 | 0.286 |
| 506 | 1.5 o2(c) + 1 adp(c) + pi(c) + 2 co2(c) + 4 glyc(c) = 1 atp(c) + 6 h2o(c) + 6 h(e) + 1 ac(e) + 3 succ(e) | 1.5 | 0.250 |
| 1443 | 1.5 o2(c) + 3 nadp(c) + adp(c) + pi(c) + 2 glyc(c) + 2 cit(e) = 3 nadph(c) + atp(c) + h2o(c) + 3.5 h(e) + 3 co2(c) + 3.5 ac(e) + 2 mal-L(e) | 1.5 | 0.250 |
| 1496 | 1.5 o2(c) + 1 nadp(c) + adp(c) + pi(c) + 4 glyc(c) = 2 etoh(c) + 1 nadph(c) + atp(c) + 4 h2o(c) + 2 h(e) + 2 co2(c) + 2 lac-L(e) | 1.5 | 0.250 |
| 2147 | 1.5 o2(c) + 2 nadp(c) + 1 adp(c) + 1 pi(c) + 3 glyc(c) + cit(e) = 2 nadph(c) + 1 atp(c) + 3 h2o(c) + 2 h(e) + 3 co2(c) + 2 ac(e) + mal-L(e) + 1 btd-RR(c) | 1.5 | 0.250 |
| 494 | 1.5 o2(c) + 1 adp(c) + pi(c) + 4 co2(c) + 6 glyc(c) = 1 atp(c) + 8 h2o(c) + 10 h(e) + 1 ac(e) + 5 succ(e) | 1.5 | 0.167 |
| 1382 | 1.9 o2(c) + 4.8 nadp(c) + 1.2 adp(c) + 1.2 pi(c) + 1.8 glyc(c) = 4.8 nadph(c) + 1.2 atp(c) + 1.6 h2o(c) + 4.6 h(e) + 3.4 co2(c) + ac(e) | 1.583333333 | 0.667 |
| 1435 | 1.6 o2(c) + 2.4 nadp(c) + adp(c) + pi(c) + 1.8 glyc(c) + 1.4 cit(e) = 2.4 nadph(c) + atp(c) + 1.6 h2o(c) + 2.8 h(e) + 2.6 co2(c) + 2.8 ac(e) + 1.4 mal-L(e) | 1.6 | 0.313 |
| 1053 | etoh(c) + 1.625 o2(c) + 2.5 nadp(c) + adp(c) + pi(c) + 1.25 glyc(c) = 2.5 nadph(c) + atp(c) + 2 h2o(c) + 3.75 h(e) + 1.25 co2(c) + 2.25 ac(e) | 1.625 | 0.444 |
| 496, 508, 510, 514, 516, 517, 519, 521, 522 | 5 o2(c) + 3 adp(c) + 3 pi(c) + 4 glyc(c) = 3 atp(c) + 10 h2o(c) + 3 h(e) + 2 co2(c) + 3 ac(e) + mal-L(e) | 1.666666667 | 0.750 |
| 2585 | 1.92857 o2(c) + 5.14286 nadp(c) + 1.14286 adp(c) + 1.14286 pi(c) + 3.85714 glyc(c) + cit(e) = etoh(c) + 5.14286 nadph(c) + 1.14286 atp(c) + 3.42857 h2o(c) + h(e) + 6.57143 co2(c) + lac-L(e) + 1.5 actn-R(e) | 1.687494531 | 0.235 |
| 2041, 2201 | 3.33333 etoh(c) + 5.66667 o2(c) + 11.3333 nadp(c) + 3.33333 adp(c) + 3.33333 pi(c) + 5.66667 glyc(c) + 2 cit(e) = 11.3333 nadph(c) + 3.33333 atp(c) + 5.66667 h2o(c) + 11 h(e) + 9.66667 co2(c) + 11 ac(e) + btd-RR(c) | 1.7000027 | 0.303 |
| 1193, 1551 | 3 etoh(c) + 6 o2(c) + 16.5 nadp(c) + 3.5 adp(c) + 3.5 pi(c) + 2.75 glyc(c) + 3.25 cit(e) = 16.5 nadph(c) + 3.5 atp(c) + h2o(c) + 9.5 h(e) + 14.75 co2(c) + 9.5 ac(e) | 1.714285714 | 0.389 |
| 2592, 2598, 2600, 2601, 2602, 2603, 2604, 2605 | 3.5 o2(c) + 2 nadp(c) + 2 adp(c) + 2 pi(c) + 4 glyc(c) = 2 nadph(c) + 2 atp(c) + 8 h2o(c) + h(e) + 4 co2(c) + ac(e) + 1.5 actn-R(e) | 1.75 | 0.500 |
| 417 | 3.5 o2(c) + 2 adp(c) + 2 pi(c) + 3 co2(c) + 5 glyc(c) = 2 atp(c) + 8 h2o(c) + 11 h(e) + ac(e) + 4 mal-L(e) | 1.75 | 0.400 |
| 1268, 1275, 1277, 1278, 1279 | 4 etoh(c) + 3.5 o2(c) + 6 nadp(c) + 2 adp(c) + 2 pi(c) + cit(e) = 6 nadph(c) + 2 atp(c) + 2 h2o(c) + 5 h(e) + 4 co2(c) + 5 ac(e) | 1.75 | 0.400 |
| 2551 | 3.5 o2(c) + 6 nadp(c) + 2 adp(c) + 2 pi(c) + 5 glyc(c) = 6 nadph(c) + 2 atp(c) + 6 h2o(c) + 7 h(e) + 5 co2(c) + 3 ac(e) + actn-R(e) | 1.75 | 0.400 |
| 1182, 1197, 1200, 1537, 1555, 1558 | 1.75 o2(c) + 5 nadp(c) + adp(c) + 1 pi(c) + 2.5 glyc(c) + 1 cit(e) = 5 nadph(c) + atp(c) + 1 h2o(c) + 4.5 h(e) + 4.5 co2(c) + 4.5 ac(e) | 1.75 | 0.286 |
| 1194, 1198, 1201, 1552, 1556, 1559 | 1.75 o2(c) + 5 nadp(c) + adp(c) + pi(c) + 2.5 glyc(c) + 1 cit(e) = 5 nadph(c) + atp(c) + 1 h2o(c) + 4.5 h(e) + 4.5 co2(c) + 4.5 ac(e) | 1.75 | 0.286 |
| 2166, 2197 | 1.75 o2(c) + 5 nadp(c) + 1 adp(c) + 1 pi(c) + 3.5 glyc(c) + cit(e) = 5 nadph(c) + 1 atp(c) + 2 h2o(c) + 3.5 h(e) + 5.5 co2(c) + 3.5 ac(e) + 1 btd-RR(c) | 1.75 | 0.222 |
| 2587 | 3.5 o2(c) + 10 nadp(c) + 2 adp(c) + 2 pi(c) + 7 glyc(c) + 2 cit(e) = 10 nadph(c) + 2 atp(c) + 4 h2o(c) + 9 h(e) + 9 co2(c) + 7 ac(e) + 2 lac-L(e) + actn-R(e) | 1.75 | 0.222 |
| 2094 | 1.75 o2(c) + 8 nadp(c) + adp(c) + pi(c) + 9 glyc(c) = 4 etoh(c) + 8 nadph(c) + atp(c) + 4.5 h2o(c) + 7 h(e) + 9 co2(c) + 2.5 btd-RR(c) | 1.75 | 0.111 |
| 1433 | 2.33333 o2(c) + 1.33333 nadp(c) + 1.33333 adp(c) + 1.33333 pi(c) + 2.66667 glyc(c) = etoh(c) + 1.33333 nadph(c) + 1.33333 atp(c) + 4.33333 h2o(c) + 1.66667 h(e) + 2.66667 co2(c) + 1.66667 ac(e) | 1.750001875 | 0.500 |
| 1432, 1450, 1452, 1457, 1459, 1461, 1463, 1465, 1467 | 1.83333 o2(c) + 1 nadp(c) + adp(c) + pi(c) + 1.33333 glyc(c) = 1 nadph(c) + atp(c) + 3 h2o(c) + 1.16667 h(e) + 1.66667 co2(c) + 1.16667 ac(e) | 1.83333 | 0.750 |
| 346 | etoh(c) + 1.83333 o2(c) + adp(c) + pi(c) + 1.66667 co2(c) + 1.66667 glyc(c) = atp(c) + 3.66667 h2o(c) + 5 h(e) + ac(e) + 1.66667 mal-L(e) | 1.83333 | 0.375 |
| 1444 | 1.83333 o2(c) + 5 nadp(c) + adp(c) + pi(c) + 2.66667 glyc(c) + 1.33333 cit(e) = 5 nadph(c) + atp(c) + 1.66667 h2o(c) + 4.5 h(e) + 4.33333 co2(c) + 3.16667 ac(e) + 1.33333 succ(e) | 1.83333 | 0.250 |
| 1426 | 1.83333 o2(c) + 16 nadp(c) + adp(c) + 2 h2o(c) + pi(c) + 6.33333 glyc(c) + 5 cit(e) = 16 nadph(c) + atp(c) + 13.6667 h(e) + 11.6667 co2(c) + 8.66667 ac(e) + 5 succ(e) | 1.83333 | 0.088 |
| 1479, 1543 | 2.75 o2(c) + 10.5 nadp(c) + 1.5 adp(c) + h2o(c) + 1.5 pi(c) + 2.25 glyc(c) + 2.25 cit(e) = 10.5 nadph(c) + 1.5 atp(c) + 5 h(e) + 10.25 co2(c) + 5 ac(e) | 1.833333333 | 0.333 |
| 1436 | 2.35714 o2(c) + 4.28571 nadp(c) + 1.28571 adp(c) + 1.28571 pi(c) + 2.71429 glyc(c) + cit(e) = 4.28571 nadph(c) + 1.28571 atp(c) + 2.85714 h2o(c) + 4 h(e) + 4.14286 co2(c) + 3 ac(e) + succ(e) | 1.833337222 | 0.346 |
| 2148 | 3.16667 o2(c) + 4.66667 nadp(c) + 1.66667 adp(c) + 1.66667 pi(c) + 6.33333 glyc(c) + cit(e) = 4.66667 nadph(c) + 1.66667 atp(c) + 6.66667 h2o(c) + 4.33333 h(e) + 6.33333 co2(c) + 3.33333 ac(e) + succ(e) + 2 btd-RR(c) | 1.8999982 | 0.227 |
| 2489, 2637 | 2.66667 etoh(c) + 5.33333 o2(c) + 10.6667 nadp(c) + 2.66667 adp(c) + 2.66667 pi(c) + 5.33333 glyc(c) + 2 cit(e) = 10.6667 nadph(c) + 2.66667 atp(c) + 5.33333 h2o(c) + 10 h(e) + 9.33333 co2(c) + 10 ac(e) + actn-R(e) | 1.99999625 | 0.267 |
| 500 | 2 o2(c) + adp(c) + pi(c) + 2 glyc(c) = atp(c) + 4 h2o(c) + 3 h(e) + ac(e) + mal-L(e) | 2 | 0.500 |
| 2597 | etoh(c) + 8 o2(c) + 4 nadp(c) + 4 adp(c) + 4 pi(c) + 8 glyc(c) = 4 nadph(c) + 4 atp(c) + 17 h2o(c) + 3 h(e) + 8 co2(c) + 3 ac(e) + 3 actn-R(e) | 2 | 0.444 |
| 2151 | etoh(c) + 3.2 o2(c) + 1.6 nadp(c) + 1.6 adp(c) + 1.6 pi(c) + 3.2 glyc(c) = 1.6 nadph(c) + 1.6 atp(c) + 6.2 h2o(c) + 1.8 h(e) + 3.2 co2(c) + 1.8 ac(e) + 1.2 btd-RR(c) | 2 | 0.381 |
| 513 | 2.5 etoh(c) + 4 o2(c) + 2 adp(c) + 2 pi(c) + co2(c) + 3 glyc(c) = 2 atp(c) + 8.5 h2o(c) + 5.5 h(e) + 3.5 ac(e) + 2 succ(e) | 2 | 0.364 |
| 456 | 3 etoh(c) + 6 o2(c) + 3 adp(c) + 3 pi(c) + co2(c) + 6 glyc(c) = 3 atp(c) + 12 h2o(c) + 8 h(e) + 3 ac(e) + 5 lac-L(e) + 1 mal-L(e) | 2 | 0.333 |
| 1418, 1493 | 2 etoh(c) + 4 o2(c) + 2 nadp(c) + 2 adp(c) + 2 pi(c) + 4 glyc(c) = 2 nadph(c) + 2 atp(c) + 7 h2o(c) + 6 h(e) + co2(c) + 3 ac(e) + 3 lac-L(e) | 2 | 0.333 |
| 2367 | 2 etoh(c) + 4 o2(c) + 2 adp(c) + 2 pi(c) + 4 glyc(c) = 2 atp(c) + 10 h2o(c) + 4 h(e) + 2 ac(e) + 2 succ(e) + actn-R(e) | 2 | 0.333 |
| 1854 | 2.5 etoh(c) + 4 o2(c) + 2 adp(c) + 2 pi(c) + 4 glyc(c) = 2 atp(c) + 9.5 h2o(c) + 4.5 h(e) + 2.5 ac(e) + 2 succ(e) + btd-RR(c) | 2 | 0.308 |
| 484 | 1.5 etoh(c) + 2 o2(c) + adp(c) + pi(c) + co2(c) + 2 glyc(c) = atp(c) + 4.5 h2o(c) + 3.5 h(e) + 1.5 ac(e) + lac-L(e) + succ(e) | 2 | 0.286 |
| 106, 613 | 2 o2(c) + adp(c) + 2 h(e) + pi(c) + 2 glyc(c) + 2 cit(e) = atp(c) + 3 h2o(c) + 4 co2(c) + 7 ac(e) | 2 | 0.250 |
| 328 | 2 o2(c) + adp(c) + pi(c) + 3 co2(c) + 4 glyc(c) = atp(c) + 5 h2o(c) + 9 h(e) + 1 lac-L(e) + 3 mal-L(e) | 2 | 0.250 |
| 1328 | 4 o2(c) + 18 nadp(c) + 2 adp(c) + 2 pi(c) + 8 glyc(c) = 18 nadph(c) + 2 atp(c) + h2o(c) + 21 h(e) + 9 co2(c) + 5 lac-L(e) | 2 | 0.250 |
| 1852 | 2 o2(c) + 1 adp(c) + pi(c) + 4 glyc(c) = 1 atp(c) + 6 h2o(c) + 2.5 h(e) + 1 co2(c) + 1.5 ac(e) + 1 succ(e) + 1 btd-RR(c) | 2 | 0.250 |
| 339 | 2 etoh(c) + 2 o2(c) + adp(c) + pi(c) + 4 co2(c) + 4 glyc(c) = atp(c) + 7 h2o(c) + 9 h(e) + 2 ac(e) + 4 succ(e) | 2 | 0.167 |
| 2549 | 2.42857 o2(c) + 5.14286 nadp(c) + 1.14286 adp(c) + 1.14286 pi(c) + 2.85714 glyc(c) = 5.14286 nadph(c) + 1.14286 atp(c) + 3.42857 h2o(c) + 4 h(e) + 4.57143 co2(c) + actn-R(e) | 2.124993438 | 0.400 |
| 1639, 1647, 1649, 1650, 1651 | 5 etoh(c) + 4.5 o2(c) + 6 nadp(c) + 2 adp(c) + 2 pi(c) + cit(e) = 6 nadph(c) + 2 atp(c) + 3 h2o(c) + 6 h(e) + 4 co2(c) + 6 ac(e) | 2.25 | 0.333 |
| 397 | 4.5 o2(c) + 2 adp(c) + 2 pi(c) + 5 co2(c) + 7 glyc(c) = 2 atp(c) + 10 h2o(c) + 17 h(e) + ac(e) + 6 mal-L(e) | 2.25 | 0.286 |
| 1039 | 1 etoh(c) + 2.25 o2(c) + 5 nadp(c) + adp(c) + 1 pi(c) + 2.5 glyc(c) = 5 nadph(c) + atp(c) + 2 h2o(c) + 7.5 h(e) + 2.5 co2(c) + 3.5 ac(e) | 2.25 | 0.286 |
| 1087, 1164, 1514 | etoh(c) + 2.25 o2(c) + 9 nadp(c) + 1 adp(c) + pi(c) + 4.5 glyc(c) + 2 cit(e) = 9 nadph(c) + 1 atp(c) + 9.5 h(e) + 6.5 co2(c) + 7.5 ac(e) + 2 lac-L(e) | 2.25 | 0.133 |
| 507 | 2 etoh(c) + 7 o2(c) + 3 adp(c) + 3 pi(c) + 4 glyc(c) = 3 atp(c) + 12 h2o(c) + 5 h(e) + 2 co2(c) + 5 ac(e) + mal-L(e) | 2.333333333 | 0.500 |
| 489 | 3.5 etoh(c) + 7 o2(c) + 3 adp(c) + 3 pi(c) + co2(c) + 7 glyc(c) = 3 atp(c) + 13.5 h2o(c) + 12.5 h(e) + 12.5 ac(e) + mal-L(e) | 2.333333333 | 0.286 |
| 1492 | 2.33333 etoh(c) + 4.66667 o2(c) + 2 nadp(c) + 2 adp(c) + 2 pi(c) + 4.66667 glyc(c) = 2 nadph(c) + 2 atp(c) + 8 h2o(c) + 6.66667 h(e) + co2(c) + 2.33333 ac(e) + 4.33333 lac-L(e) | 2.333335 | 0.286 |
| 1434, 1454, 1456, 1458, 1460, 1462, 1464, 1466, 1468 | 2.5 o2(c) + 1 nadp(c) + adp(c) + pi(c) + 2 glyc(c) = 1 nadph(c) + atp(c) + 4 h2o(c) + 2 h(e) + 2 co2(c) + 2 ac(e) | 2.5 | 0.500 |
| 1449 | etoh(c) + 3.75 o2(c) + 1.5 nadp(c) + 1.5 adp(c) + 1.5 pi(c) + 2 glyc(c) = 1.5 nadph(c) + 1.5 atp(c) + 5.5 h2o(c) + 2.75 h(e) + 2.5 co2(c) + 2.75 ac(e) | 2.5 | 0.500 |
| 1122 | 2.5 etoh(c) + 5 o2(c) + 2 nadp(c) + 2 adp(c) + 2 pi(c) + 5 glyc(c) = 2 nadph(c) + 2 atp(c) + 8.5 h2o(c) + 9.5 h(e) + co2(c) + 9.5 ac(e) | 2.5 | 0.267 |
| 1185, 1541 | etoh(c) + 7.5 o2(c) + 33 nadp(c) + 3 adp(c) + 7 h2o(c) + 3 pi(c) + 5.5 glyc(c) + 7.5 cit(e) = 33 nadph(c) + 3 atp(c) + 16 h(e) + 31.5 co2(c) + 16 ac(e) | 2.5 | 0.214 |
| 1190, 1547 | 2.5 o2(c) + 11 nadp(c) + 1 adp(c) + 2 h2o(c) + pi(c) + 2.5 glyc(c) + 2.5 cit(e) = 11 nadph(c) + 1 atp(c) + 6 h(e) + 10.5 co2(c) + 6 ac(e) | 2.5 | 0.200 |
| 1189, 1546 | 5 o2(c) + 22 nadp(c) + 2 adp(c) + h2o(c) + 2 pi(c) + 11 glyc(c) + 5 cit(e) = 3 etoh(c) + 22 nadph(c) + 2 atp(c) + 18 h(e) + 21 co2(c) + 18 ac(e) | 2.5 | 0.125 |
| 2593 | 2.5 o2(c) + 4 nadp(c) + adp(c) + 1 pi(c) + 5 glyc(c) + 3 cit(e) = 4 nadph(c) + atp(c) + 4 h2o(c) + 5 h(e) + 5 co2(c) + 5 ac(e) + 3 mal-L(e) + 1.5 actn-R(e) | 2.5 | 0.125 |
| 1360 | 5.25 o2(c) + 18 nadp(c) + 2 adp(c) + 2 pi(c) + 5.5 glyc(c) = 18 nadph(c) + 2 atp(c) + h2o(c) + 18.5 h(e) + 11.5 co2(c) + 2.5 ac(e) | 2.625 | 0.364 |
| 1819 | 5.5 o2(c) + 2 adp(c) + 2 pi(c) + 6 co2(c) + 10 glyc(c) = 2 atp(c) + 13 h2o(c) + 22 h(e) + 8 mal-L(e) + btd-RR(c) | 2.75 | 0.200 |
| 1052 | 3 etoh(c) + 3.83333 o2(c) + 10 nadp(c) + 1.33333 adp(c) + 1.33333 pi(c) + 1.66667 glyc(c) = 10 nadph(c) + 1.33333 atp(c) + h2o(c) + 11.6667 h(e) + 5 co2(c) + 3 ac(e) | 2.875004688 | 0.286 |
| 1439 | 3 o2(c) + 4.5 nadp(c) + adp(c) + pi(c) + 2.5 glyc(c) = 4.5 nadph(c) + atp(c) + 3 h2o(c) + 5.25 h(e) + 4 co2(c) + 1.75 ac(e) | 3 | 0.400 |
| 511 | 1.5 etoh(c) + 3 o2(c) + 1 adp(c) + pi(c) + 2 glyc(c) = 1 atp(c) + 5.5 h2o(c) + 3.5 h(e) + 2.5 ac(e) + 1 succ(e) | 3 | 0.286 |
| 505 | 3 o2(c) + 1 adp(c) + pi(c) + 2 co2(c) + 4 glyc(c) = 1 atp(c) + 6 h2o(c) + 9 h(e) + 1 ac(e) + 3 mal-L(e) | 3 | 0.250 |
| 490 | 2 etoh(c) + 3 o2(c) + adp(c) + 1 pi(c) + 1 co2(c) + 3 glyc(c) = atp(c) + 6 h2o(c) + 6 h(e) + 5 ac(e) + 1 succ(e) | 3 | 0.200 |
| 1181, 1187, 1196, 1199, 1536, 1544, 1554, 1557 | 3 o2(c) + 15 nadp(c) + adp(c) + 4 h2o(c) + pi(c) + 2.5 glyc(c) + 3.5 cit(e) = 15 nadph(c) + atp(c) + 7 h(e) + 14.5 co2(c) + 7 ac(e) | 3 | 0.167 |
| 2533 | 3 o2(c) + 4 nadp(c) + adp(c) + pi(c) + 6 glyc(c) = 2 etoh(c) + 4 nadph(c) + atp(c) + 7 h2o(c) + 3 h(e) + 6 co2(c) + 2 actn-R(e) | 3 | 0.167 |
| 2164, 2193 | 6 o2(c) + 30 nadp(c) + 2 adp(c) + h2o(c) + 2 pi(c) + 12 glyc(c) + 7 cit(e) = 30 nadph(c) + 2 atp(c) + 7 h(e) + 36 co2(c) + 7 ac(e) + 7 btd-RR(c) | 3 | 0.105 |
| 2584 | 6 o2(c) + 30 nadp(c) + 2 adp(c) + h2o(c) + 2 pi(c) + 12 glyc(c) + 7 cit(e) = 30 nadph(c) + 2 atp(c) + 14 h(e) + 29 co2(c) + 7 ac(e) + 7 lac-L(e) + 3.5 actn-R(e) | 3 | 0.105 |
| 1174, 1528 | 3 o2(c) + 30 nadp(c) + adp(c) + 14 h2o(c) + pi(c) + 5 glyc(c) + 11 cit(e) = 30 nadph(c) + atp(c) + 17 h(e) + 27 co2(c) + 17 ac(e) + 5 mal-L(e) | 3 | 0.063 |
| 2149 | 3.25 o2(c) + 4 nadp(c) + 1 adp(c) + pi(c) + 5 glyc(c) = 4 nadph(c) + 1 atp(c) + 5.5 h2o(c) + 5 h(e) + 5 co2(c) + 2 ac(e) + 1.5 btd-RR(c) | 3.25 | 0.200 |
| 1453 | 1 etoh(c) + 3.5 o2(c) + 1 nadp(c) + 1 adp(c) + pi(c) + 2 glyc(c) = 1 nadph(c) + 1 atp(c) + 5 h2o(c) + 3 h(e) + 2 co2(c) + 3 ac(e) | 3.5 | 0.333 |
| 1248, 1616 | 1.75 etoh(c) + 3.5 o2(c) + 5 nadp(c) + 1 adp(c) + pi(c) + 2.5 glyc(c) + cit(e) = 5 nadph(c) + 1 atp(c) + 2.75 h2o(c) + 6.25 h(e) + 4.5 co2(c) + 6.25 ac(e) | 3.5 | 0.190 |
| 326 | etoh(c) + 3.5 o2(c) + adp(c) + pi(c) + 5 co2(c) + 5 glyc(c) = atp(c) + 7 h2o(c) + 15 h(e) + ac(e) + 5 mal-L(e) | 3.5 | 0.167 |
| 2342 | 7 o2(c) + 2 adp(c) + 2 pi(c) + 8 co2(c) + 12 glyc(c) = 2 atp(c) + 16 h2o(c) + 28 h(e) + 10 mal-L(e) + actn-R(e) | 3.5 | 0.167 |
| 1235, 1601 | etoh(c) + 7 o2(c) + 30 nadp(c) + 2 adp(c) + 7 h2o(c) + 2 pi(c) + 5 glyc(c) + 7 cit(e) = 30 nadph(c) + 2 atp(c) + 15 h(e) + 29 co2(c) + 15 ac(e) | 3.5 | 0.154 |
| 335 | 3.5 o2(c) + adp(c) + pi(c) + 5 co2(c) + 7 glyc(c) = atp(c) + 8 h2o(c) + 17 h(e) + 3 ac(e) + 5 mal-L(e) | 3.5 | 0.143 |
| 493 | 4 o2(c) + 1 adp(c) + pi(c) + 4 co2(c) + 6 glyc(c) = 1 atp(c) + 8 h2o(c) + 15 h(e) + 1 ac(e) + 5 mal-L(e) | 4 | 0.167 |
| 1437 | 4 o2(c) + 4 nadp(c) + adp(c) + pi(c) + 5 glyc(c) + 3 cit(e) = 4 nadph(c) + atp(c) + 4 h2o(c) + 8 h(e) + 5 co2(c) + 8 ac(e) + 3 mal-L(e) | 4 | 0.125 |
| 2037, 2196 | etoh(c) + 4.25 o2(c) + 17 nadp(c) + 1 adp(c) + pi(c) + 8.5 glyc(c) + 4 cit(e) = 17 nadph(c) + 1 atp(c) + 13.5 h(e) + 16.5 co2(c) + 13.5 ac(e) + 2 btd-RR(c) | 4.25 | 0.074 |
| 1447 | 4.5 o2(c) + 9 nadp(c) + adp(c) + 1 pi(c) + 4 glyc(c) = 9 nadph(c) + atp(c) + 3 h2o(c) + 10.5 h(e) + 7 co2(c) + 2.5 ac(e) | 4.5 | 0.250 |
| 1088, 1165, 1515 | 5.5 etoh(c) + 4.5 o2(c) + 27 nadp(c) + adp(c) + 9 h2o(c) + pi(c) + 4.5 glyc(c) + 6.5 cit(e) = 27 nadph(c) + atp(c) + 18.5 h(e) + 20 co2(c) + 12 ac(e) + 6.5 lac-L(e) | 4.5 | 0.061 |
| 1038 | 7 etoh(c) + 9.5 o2(c) + 30 nadp(c) + 2 adp(c) + h2o(c) + 2 pi(c) + 5 glyc(c) = 30 nadph(c) + 2 atp(c) + 35 h(e) + 15 co2(c) + 7 ac(e) | 4.75 | 0.167 |
| 1470 | 9.5 o2(c) + 30 nadp(c) + 2 adp(c) + h2o(c) + 2 pi(c) + 12 glyc(c) + 7 cit(e) = 30 nadph(c) + 2 atp(c) + 21 h(e) + 29 co2(c) + 14 ac(e) + 7 lac-L(e) | 4.75 | 0.105 |
| 2610 | 4.75 o2(c) + 15 nadp(c) + adp(c) + pi(c) + 9.5 glyc(c) + 3.5 cit(e) = 3.5 etoh(c) + 15 nadph(c) + atp(c) + 6.5 h2o(c) + 21.5 co2(c) + 5.25 actn-R(e) | 4.75 | 0.077 |
| 2630 | 4.75 o2(c) + 15 nadp(c) + 1 adp(c) + pi(c) + 9.5 glyc(c) + 3.5 cit(e) = 3.5 etoh(c) + 15 nadph(c) + 1 atp(c) + 6.5 h2o(c) + 21.5 co2(c) + 5.25 actn-R(e) | 4.75 | 0.077 |
| 2594 | 5.5 o2(c) + 10 nadp(c) + 1 adp(c) + pi(c) + 11 glyc(c) + 3 cit(e) = 10 nadph(c) + 1 atp(c) + 10 h2o(c) + 11 h(e) + 11 co2(c) + 8 ac(e) + 3 succ(e) + 3 actn-R(e) | 5.5 | 0.071 |
| 1175, 1529 | 5.5 o2(c) + 60 nadp(c) + adp(c) + 24 h2o(c) + pi(c) + 10 glyc(c) + 16 cit(e) = 60 nadph(c) + atp(c) + 32 h(e) + 52 co2(c) + 27 ac(e) + 5 succ(e) | 5.5 | 0.038 |
| 2093 | 11.5 o2(c) + 48 nadp(c) + 2 adp(c) + 2 pi(c) + 18 glyc(c) = 48 nadph(c) + 2 atp(c) + h2o(c) + 46 h(e) + 34 co2(c) + 5 btd-RR(c) | 5.75 | 0.111 |
| 1245, 1613 | 3 etoh(c) + 6 o2(c) + 15 nadp(c) + adp(c) + 1 h2o(c) + pi(c) + 2.5 glyc(c) + 3.5 cit(e) = 15 nadph(c) + atp(c) + 10 h(e) + 14.5 co2(c) + 10 ac(e) | 6 | 0.111 |
| 1438 | 8.5 o2(c) + 10 nadp(c) + adp(c) + pi(c) + 11 glyc(c) + 3 cit(e) = 10 nadph(c) + atp(c) + 10 h2o(c) + 17 h(e) + 11 co2(c) + 14 ac(e) + 3 succ(e) | 8.5 | 0.071 |

**Table III-3**. All 531 unique overall stoichiometries of EFMs that generate ATP from any combination of the 5 inputs: glycerol, oxygen, acetate, ethanol or citrate. EFMs are sorted first by oxygen needed per ATP, then by substrate needed per ATP (all substrates counted equally). EFMs in yellow background are anaerobic modes wih glycerol al sole substrate. Pink background: anaerobic mode with citrate as sole substrate. Green background indicates EFMs that were found to be optimal for ATP yield under the optimization problem defined in Methods in the main text.
